# Development of a gender score in a representative German population sample and its association with diverse social positions

**DOI:** 10.3389/fepid.2022.914819

**Published:** 2022-08-24

**Authors:** Lisa Wandschneider, Odile Sauzet, Oliver Razum, Céline Miani

**Affiliations:** ^1^Department of Epidemiology and International Public Health, School of Public Health, Bielefeld University, Bielefeld, Germany; ^2^Center for Statistics, Bielefeld University, Bielefeld, Germany; ^3^Research Institute Social Cohesion (RISC), Bielefeld University, Bielefeld, Germany

**Keywords:** gender analysis, measurement, secondary data analysis, intersectionality, relational theory, social epidemiology

## Abstract

**Background:**

Gender as a relational concept is rarely considered in epidemiology. However, an in-depth reflection on gender conceptualisation and operationalisation can advance gender analysis in quantitative health research, allowing for more valid evidence to support public health interventions. We constructed a context-specific gender score to assess how its discriminatory power differed in sub-groups defined by social positions relevant to intersectional analyses, i.e., sex/gender, race, class, age and sexual attraction.

**Methods:**

We created a gender score with the help of multivariable logistic regression models and conditional probabilities based on gendered social practices and expressed on a masculinity-femininity continuum, using data of the German Socioeconomic Panel. With density plots, we exploratively compared distributions of gendered social practices and their variation across social groups.

**Results:**

We included 13 gender-related variables to define a gender score in our sample (*n* = 20,767). Variables on family and household structures presented with the highest weight for the gender score. When comparing social groups, we saw that young individuals, those without children, not living with a partner or currently living in a same-sex/gender partnership, showed more overlap between feminine/masculine social practices among females and males.

**Conclusions:**

The distribution of gendered social practices differs among social groups, which empirically backs up the theoretical notion of gender being a context-specific construct. Economic participation and household structures remain essential drivers of heterogeneity in practices among women and men in most social positions. The gender score can be used in epidemiology to support concerted efforts to overcome these gender (in)equalities—which are important determinants of health inequalities.

## Introduction

There is a shift in how epidemiologists and quantitative public health researchers approach gender analysis, with more and more studies examining gender as a social construct describing the expectations, norms, roles, responsibilities and power relations that fall upon individuals based on their presenting gender (1, 2). Gender analysis seeks to explore the implications of gender in access to resources and care, differing needs, experiences and health outcomes in the health care system, and how policies and programs address these (3). A multitude of primary data collection instruments assessing the multiple dimensions of gender is readily available for epidemiological research (4, 5). In addition, routinely collected data from registries, national statistics, health monitoring and population surveys contain a wide range of variables describing gendered behaviours, roles and (in)equalities and are therefore frequently used in gender analysis in health research (6, 7).

In secondary data analysis, the approach of gender-related variables has gained increasing attention over the past decade (8–12). As a survey-based approach, it uses variables not specifically collected to capture gender. Yet, these provide, to some extent, reflections or expressions of gendered performance, norms and relations. They pertain to different aspects of life, from caregiving activities, to the distribution of labour and economic resources, and the processes of social support and discrimination (11). These variables allow to identify norms, behaviours and relational characteristics that differentiate between women and men (9, 13–15), girls and boys (16, 17) and most recently also between gender-diverse people (11). Depending on the exact operationalisation, they also enable to generate population-specific gender constructs and try to overcome the limitations of categorical and binary variables (12, 18).

Yet, these approaches rarely explore the relational and intersectional aspects of gender—neither on a theory-informed level nor in the concrete operationalisation of such characteristics. In this analysis, we will use intersectionality as an analytical perspective to assess how gendered practices are performed in different social positions. By now, intersectionality is considered to be the preferred approach for the multidisciplinary analysis of the complex interplay between social positions and power relations (19). Intersectionality theory originated from critical race theory and gender analysis (19, 20) arguing that socially ascribed attributes, so called “social positions”, such as (but not limited to) race, sex/gender, sexual attraction, socioeconomic status and disability intersect at the individual level. Simultaneously, these social positions at the individual level reflect and are formed by interlocking systems of oppression and privilege at the macro level (e.g., as racism, classism and sexism) (21).

In this light, we theorise gender as a relational process, based on a relational theory of gender (22), following the current state of art of theoretical gender concepts in health research (23). The relational theory of gender builds on social constructivism and emphasises the relations between and among individuals that shape gender as a social process. It also insists on analysing the social practices that are modifying and interacting with this process. Within this notion, Raewyn Connell developed a multilevel understanding of gender “embracing at the same time economic relations, power relations, affective relations and symbolic relations; and operating simultaneously at intrapersonal, interpersonal, institutional and society-wide levels” (24). This understanding is rooted in feminist sociology and goes beyond conceptualising gender exclusively as an identity or trait characteristic—as it has been the case in most of the gender analyses in health research. Thus, gender is constructed by micro-, meso- and macro factors; such as personal interaction, institutional power relations (e.g., sexism or trans-/homophobia) or economic structures (e.g., the gender pay gap). Thereby, behaviours of individuals can create or perpetuate societal gender norms at the population level through daily and recurrent actions (“doing gender”) (25).

Building on the gender-related variables approach, we explicitly conceptualised gender based on the relational theory of gender by Connell (24, 26). By developing a population-specific gender measure of the gendered social practices and by comparing it across different sub-populations defined by social position, we extended Pelletier's (12) approach. We conduct an explorative examination of gender being a context-specific construct which could be relevant when analysing gendered health inequalities. To define the sub-populations, we used proxies of the social positions most frequently assessed in intersectional analyses, i.e., sex/gender, race, class, age and sexual attraction. Doing so, we aimed to answer the following questions:

(1) Which variables described gendered social practices in a representative German population sample?
(2) How did a gender score perform among different social groups?

Given the assumption that power and disadvantages associated with social positions depend on time and context, our analyses were rather explorative and can be understood as a first step to assess which intersections with gender were relevant in the German context.

## Materials and methods

### Data

We analysed data from the German Socio-Economic Panel (SOEP). The SOEP is a longitudinal, nationally representative household survey that has been conducted every year since 1984 (27). It encompasses subjective and objective variables of demography, work and employment, family and social networks, health, values and attitudes, and migration over the life course. Thanks to enlargement samples and oversampling, the SOEP allows for in-depth analyses of migrant populations.

We used the most recent version v35 (28) (2018), as gender-related variables on attitudes and norms have been added and updated in this wave. Individuals younger than 18 years (*n* = 86) were excluded, as well as cases with missing values in the gender-related variables (*n* = 4,026 cases where these were not part of the survey and *n* = 5,427 cases with missings). Supplementary material 1 compares the demographics of these excluded cases with our sample. Overall, the excluded cases were more likely to being male, of older age and presenting with lower socioeconomic status and an immigration history.

### Measurement

#### Gendered social practices

We used gender-related variables from a large representative dataset to construct a context-specific gender score and assess its discriminatory power. For that, we applied Pelletier et al.'s methodology to a new population and calculated a gender score describing gendered social practices and norms that are specific to the sample under investigation (12). We adopted the methodology of Pelletier et al. (12), which was originally based on Lippa and Conelly's gender diagnosticity approach (13). In a nutshell, this diagnostic approach assesses the presence or absence of gendered dimensions in an individual, relying on the sex-differential distribution of these dimensions in a given population (29). While gender is a distinct concept from sex assigned at birth (defined as biological, physiological and hormonal characteristics “enabling sexual reproduction” (30), these two concepts are interdependent. For example, based on varying sets of norms prescribed on individuals according to their sex assigned at birth in socialisation processes, we can observe systemic differences between women, men and gender-diverse people at large scale. The gender diagnostic approach builds on this understanding of gender as a “differential social construct”(29) and indicates “how much an individual shares one/several gendered dimension(s) of a given population, place and time” (29). It thereby allows operationalising gender as context- and population-specific and aligning with a relational understanding of gender.

#### Variable selection

To transparently report on the development the gender score, we used the TRIPOD checklist (Supplementary material 2). Though initially developed for and mostly applied in clinical settings, the checklist facilitated transparent reporting. We first screened the SOEP Core dataset systematically to identify gender-related variables. We conducted an online search on the companion.soep.de website and screened all the SOEP Topics for variables that captured gendered social processes, practices and attitudes. All variables were checked against the theoretical understanding of gender based on Connell (24). We excluded variables that (1) reported direct health outcomes since we intend to apply the score in future epidemiological studies, (2) did not describe social gendered processes, practices or beliefs (e.g., height or weight), (3) captured outcomes of gendered inequalities but not the gendered processes itself (such as employment status, formal education, income), and (4) had more than 25% missing cases (which also lead to the exclusion of parent-specific questions).

To validate the findings of our hand-search and identify potential gender-related variables, we additionally conducted bivariate analyses with all variables of the SOEP Core dataset (two-tailed *t*-tests and Chi-2 tests, with Bonferonni correction to avoid Type 1 errors). Given the lack of a gold standard to measure gender (31) and the lack of data to describe one's gender in more diverse terms than women and men in the SOEP, we used sex assigned at birth as a proxy for the socially constructed norms and expectations related to being perceived and/or seeing themselves as a woman or a man (12). The SOEP does not contain an open answer option to enter one's gender identity and a by-law mandatory third gender option has only been introduced by the end of 2018. All variables that showed a significant association with sex assigned at birth (= outcome variable in the prediction model) were then again checked with the aforementioned exclusion criteria. We further excluded variables that described similar phenomena either based on the strength of the bivariate association (where we retained the stronger association) or chose variables that were identified in prior research as relevant variables of gendered practices (9, 11, 12, 16).

#### Constructing the gender score

We conducted a principal component analysis (PCA) to capture underlying gender-based constructs, identify the relevant variables for this sample population and thereby reduce the number of variables. With the help of hierarchical logistic regression models, we then assessed which of the gender-related variables were independently associated with sex assigned at birth as the outcome measure. Non-significant variables (p>0.05) were excluded backward-stepwise in descending order based on their *p*-value. We found no evidence for multicollinearity (maximum Variance Inflation Factor: 1.73).

The coefficient estimates obtained in the final hierarchical logistic regression model were used to calculate the conditional probability to be categorised as “female” ranging on a continuum from 0 to 1 that we interpreted as the gender score. Estimates of conditional probabilities can be used in observational studies respective of a specific exposure, in our analysis to be categorised as “female” (vs. being categorised as “male”), given the observed covariates (i.e., the 13 gender related variables identified in the final logistic regression model and constituting the gender score) (32). Pelletier et al. originally applied the terminology of propensity scores to describe the conditional probabilities. To emphasise that we do not study a causal research question, we decided to use conditional probabilities instead. Accordingly, the interpretation of the gender score used in this analysis is as followed: the higher the gender score of a participant is, the higher the levels of gendered practices and attitudes associated with being female are. In line with the original works of Pelletier et al., we described the continuum ranging from “masculine” (towards zero) to “feminine” (towards 1) gendered social practices, with “androgynous” gendered practices in-between the two poles indicating balanced levels of masculine and feminine gendered social practices (12). Individuals with similar gender score values shared similar gendered practices and attitudes.

#### Stratifying variables for sub-group comparisons

We defined age groups for the ranges of 18 to 30, 31 to 45, 46 to 60, and 61 to 75 + years (with 30 years as the first cut-off value for 15-year age groups given the average age of parents at birth of first child (33) and the average age at marriage (34) in Germany– both assumed to be relevant life events with regard to variables measuring gendered practices). The formal educational attainment was categorised in low, medium and high educational levels based on the modified classification scheme of the Comparative Analysis of Social Mobility in Industrial Nations (CASMIN) (35, 36). The household pre-government income combined income before taxes and government transfers of all household members above the age of 16 years. It was categorised with the help of quintiles into high- (highest quintile), middle- (2nd to 4th quintile) and low-income (lowest income quintile) groups. In addition, we differentiated by the German federal state of residence contrasting between East and West Germany given the historical contrasts in economic and family policies before the German reunification (37).

Since the concept of race is not used in the German context, we used migration status as a proxy for the experiences of racialisation, migratisation and “othering” among migrants because of their assumed cultural differences (38). To differentiate between migrant and non-migrant groups and within migrant groups, we used several variables to account for some of the heterogeneity. First, we used the country of birth to differentiate between individuals born in Germany or those who were born in a different country. In addition, we differentiated between the 5 most frequent countries of origin in the 2018 sample, which were Poland, Russia, Kazakhstan, Turkey and Romania. Third, we grouped the majority of countries of origin to higher-level regions of origin (Eastern Europe, Western Europe, Southwest-Asia and the Middle East). To compare these heterogeneous migrant groups beyond countries of origin, we additionally included the legal status (dichotomous variable differentiating between temporary and unlimited residence permit) and the length of stay in Germany (in years).

We also used social stratifiers that are strongly associated with a social concept of gender. Usually or currently being in a same-sex/gender partnership was based on a generated SOEP variable derived from multiple items: either self-reported sexual attraction with the options heterosexual, homosexual and bisexual; the marital status that distinguished between same-sex civil unions and opposite-sex marriage; or the relation between the head of the household and all household members (39). This approach has some limitations, most importantly that bisexual individuals, especially in longterm partnerships, could not be clearly identified, even in the longitudinal study design of the SOEP (40). We therefore distinguished between those who are usually or currently in a same-sex/gender relationship or not and those with insufficient information where the named sources did not allow attributions. Since some of the variables of social gendered practices were located at the household level, we decided to differentiate between individuals living with a partner and those who were single or did not live with their partner assuming that this might capture everyday gender relations best. The transition to parenthood is associated with changes in gender roles for women, men (41) and gender-diverse individuals who are also experiencing high levels of heteronormative discrimination (42–44). In our analysis, we therefore differentiated between parents (based on a broader social concept of parenthood i.e., individuals with at least one biological, adopted or foster child or individuals living with their partner's child in one household) and those who did not have a child.

### Analysis strategy

We first explored the distribution of the gender score by sex assigned at birth and across social groups stratified by sociodemographic variables as well as relationship and family status. We conducted multiple sensitivity analyses. To assess the gender score performance, we calculated different versions of the gender score by excluding variables based on statistical or content-related reasons. We compared the model fit of the different regression models (using the Akaike Information Criterion) and the distribution of the different gender scores to see how stable and robust the score performs. We then checked whether the variables defining the gender score varied in different sub-populations. For that we separately calculated the gender score in samples defined by the social stratifiers that showed diverging patterns in the descriptive analyses (i.e., age, migrants vs. non-migrants, region of origin, parenthood, usually or currently living in a same-sex/gender partnership and cohabitation status with a partner). In addition, we performed bootstrapping to check for model validity (TRIPOD guideline internal validity). We assessed the estimated the bias in the coefficient estimates we used for calculating the gender score (*n* = 1,000 replications) to examine the predictive accuracy of the logistic regression model.

Statistical analyses were performed using R version 3.6.3 (R Code provided in Supplementary material 3) (45). All significance tests were performed two-sided with a significance level of α = 0.05.

## Results

### Sample description

Table 1 shows the sample characteristics. The overall sample (with valid cases for the gender-related variables included in the overall gender score) included 20,767 participants, with a moderate sex imbalance of 57% females to 43% males. For the variable selection processes (PCA, logistic regressions and sensitivity analyses), 19,426 cases with complete information on all potential gender-related variables were available for the process to develop the gender score.

**Table 1:** Sample characteristics, SOEP, Germany, 2018 (*n* = 20,767).

		**Sample distribution**			
		***N***	**valid %**	**missings**	
				***N***	**%**
	**Sex assigned at birth**				
	Female	11,865	57.1	0	0.0
**Sociodemographics**	Male	8,902	42.9		
	**Age**				
	18–30 years	3,624	17.5	0	0.0
	31–45 years	5,360	25.8		
	46–60 years	6,375	30.7		
	61–75 + years	5,408	26.0		
	**Formal educational attainment**				
	Low educational attainment	6,122	30.6	0	0.0
	Middle educational attainment	8,764	43.9		
	High educational attainment	5,096	25.5		
	**Pre-government household income**				
	Lowest income quintil	2,650	12.8	0	0.0
	Middle income quintiles	13,394	64.5		
	Highest income quintil	4,723	22.7		
	**Region of residence**				
	West-Germany	15,875	76.4	0	0.0
	East-Germany	4,892	23.6		
	**Country of birth**				
	Born in Germany or immigr. <1950	17,359	83.6	0	0.0
**Migration status**	Not born in Germany	3,408	16.4		
	**Country of origin**				
	Germany	17,359	91.1	1,712	8.2
	Poland	463	2.4		
	Russia	382	2.0		
	Kazakhstan	330	1.7		
	Turkey	271	1.4		
	Romania	250	1.3		
	**Region of origin**				
	Eastern Europe	1,696	8.3	17,724	85.3
	Western Europe	455	2.2		
	Central Asia	410	2.0		
	Middle East	482	2.4		
	**Year of immigration**				
	before 2009	2,576	76.7	17,410	83.8
	2009 to 2018	781	23.3		
	**Residence status**				
	Unlimited	998	68.9	19,319	93.0
	Temporary	450	31.1		
**Relationship and** **family status**	**Parenthood**				
	Childless	5,966	28.7	0	0.0
	Parent	14,801	71.3		
	**Living with a partner**				
	Single or not living with their partner	8,993	43.4	62	0.3
	Living with a partner	11,712	56.6		
	**Usually or currently living in a same sex/gender partnership**				
	No	17,401	83.8	0	0.0
	Yes	255	1.2		
	Insufficient information	3,111	15.0		

Authors' own elaboration. Socioeconomic panel, wave v35.

### Constructing the gender score

**Figure 1:**
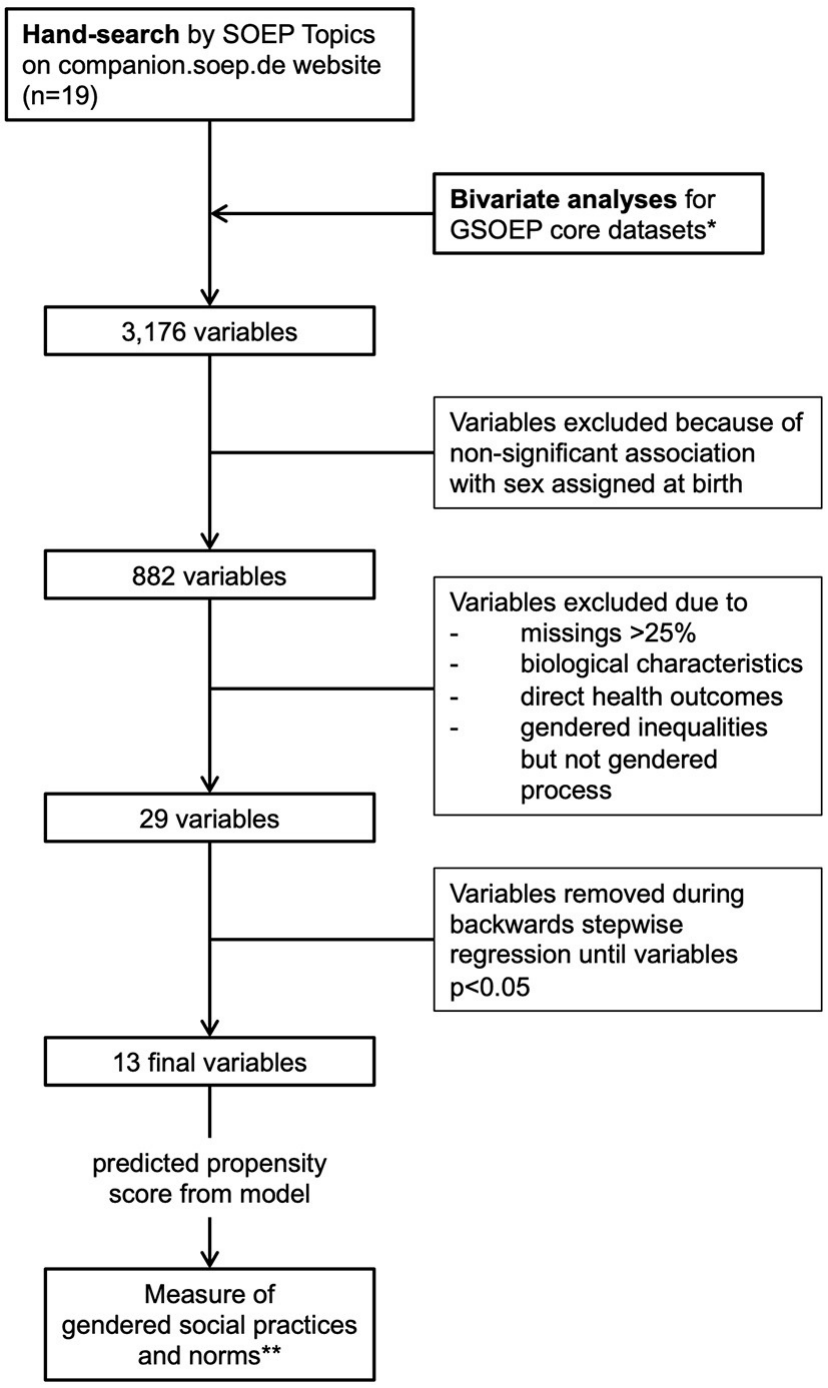
Flowchart of variable selection process for the gender score, SOEP, Germany, 2018. *we excluded datasets exclusively focused on under-aged, that did not provide valid data for the survey year and those that only contained interview data. **calculated for the overall sample. Authors' own elaboration.

Figure 1 visualises the process of constructing the gender score. Screening the SOEP core samples, we identified 29 variables. We did not identify any combination of variables that explained a large proportion of the variance, so PCA was not applicable to reduce the number of variables (for details see Supplementary material 4).

Table 2 shows the coefficients of the 13 variables that were significantly associated with sex assigned at birth in the final hierarchical logistic regression. We retained variables measuring attitudes and norms towards gender roles and gendered behaviour, also referred to as “symbolic relations” by Connell in the Relational Theory on Gender (22). These covered attitudes on marriage, discrimination and rights of LGBTQI^*^ people and roles within the family for women. Variables describing “economic and power relations” among individuals of different genders included working experience in part-time employment, hours of housework, leisure and repairs on weekdays. For the “affective relations” component of gender, we identified variables on satisfaction with housework, worries about crime in Germany and global terrorism as well as willingness to take risks. The variables relating to economic power relations, especially those on household responsibilities, presented with the highest coefficient estimates and therefore had the highest weight in the calculation of the gender score.

**Table 2:** Logistic regression coefficients of gendered social practices associated with sex assigned at birth used to construct the gender score, SOEP, Germany, 2018 (*n* = 20,767).

	**Coefficient** **estimate**	***p***
**Intercept**	-1.39	<0.001
**Symbolic relations (attitudes and norms)**		
A person who is living with their partner for the long term should get married	0.04	<0.001
Children below the age of 6 suffer if their mother works	0.12	<0.001
A same-sex couple can raise a child just as well as a man and woman	0.13	<0.001
It would be good for society if transgender people were recognised as normal	0.10	<0.001
**Economic and power relations (access to** **resources and participation)**		
Working experience part-time employment	0.18	<0.001
Hours/weekday housework	1.21	<0.001
Hours/weekday repairs	−0.80	<0.001
Hours/weekday leisure, hobbies	−0.08	<0.001
**Affective relations (emotional resources)**		
Worried about global terrorism	−0.31	<0.001
Worried about crime in Germany	−0.10	0.003
Satisfaction with housework	−0.06	<0.001
Willingness to take risks	−0.13	<0.001
Worried about own retirement pension	−0.08	0.003

Categorisation Categorisation in symbolic, economic and power as well as affective relations is based on (22). Authors' own elaboration. Socioeconomic panel, wave v35.

### Gendered social practices in social groups

**Figure 2:**
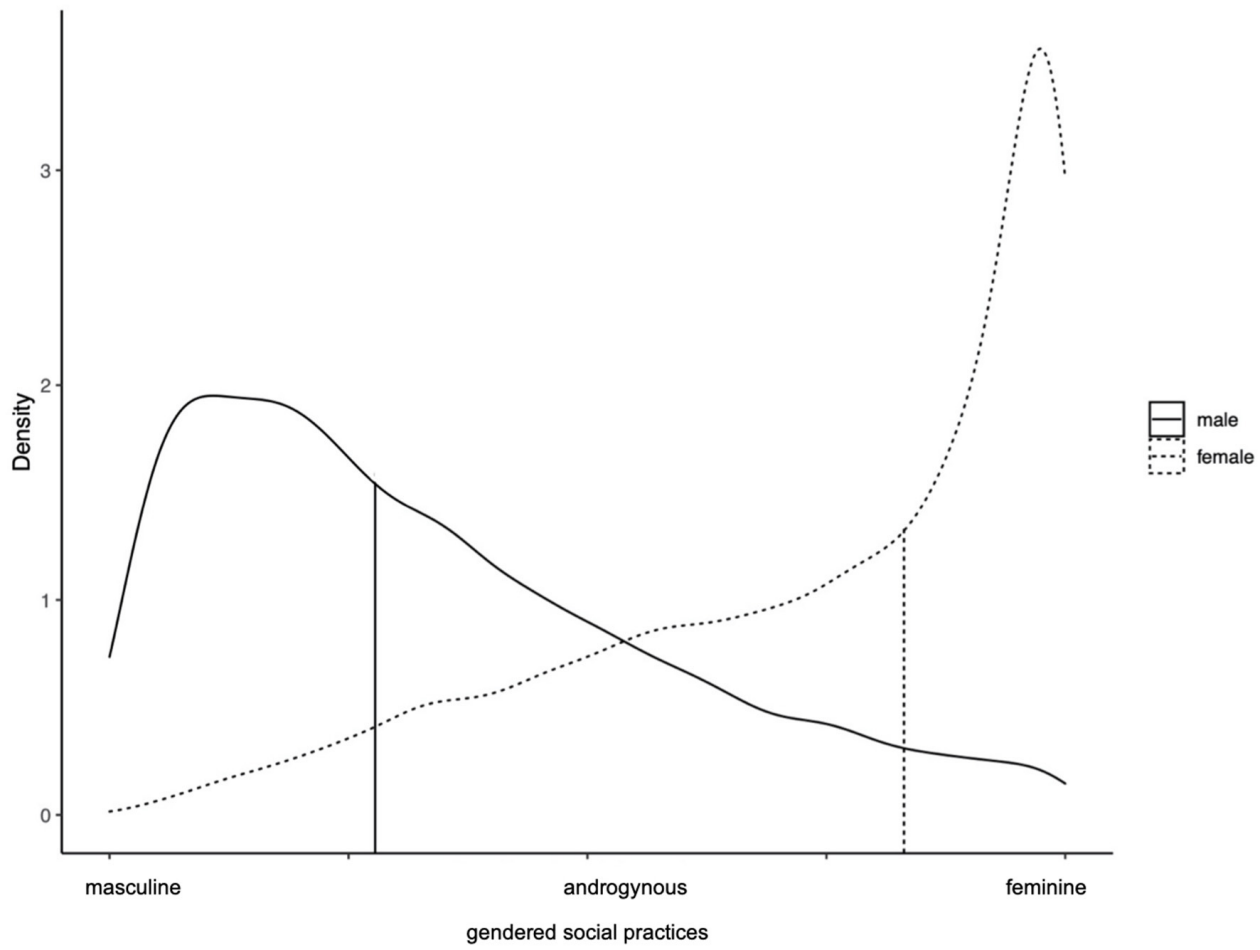
Gendered social practices in females and males, SOEP, Germany 2018 (*n* = 20,767). The vertical bars represent the median for females and males respectively. *Authors' own elaboration. Socioeconomic panel, wave v35*.

The gender score revealed clear differences in gendered social practices by sex assigned at birth. Females and males showed highly skewed distributions towards one extreme, respectively feminine and masculine social practices (Figure 2). The distribution for females was more skewed than the one for males. Still, one could observe an overlap between females and males across the gendered social practices, indicating that these were at least in parts independent from sex assigned at birth. To ease the interpretation of the visualisations of the gender score, we integrated the median score for females and males as a measure of central tendency. It indicates whether females or males rather present with clearly differentiated patters of masculine or feminine practices (median is located at the extremes of the continuum) or whether the gendered practices are rather balanced (median is closer to the middle of the continuum).

**Figure 3:**
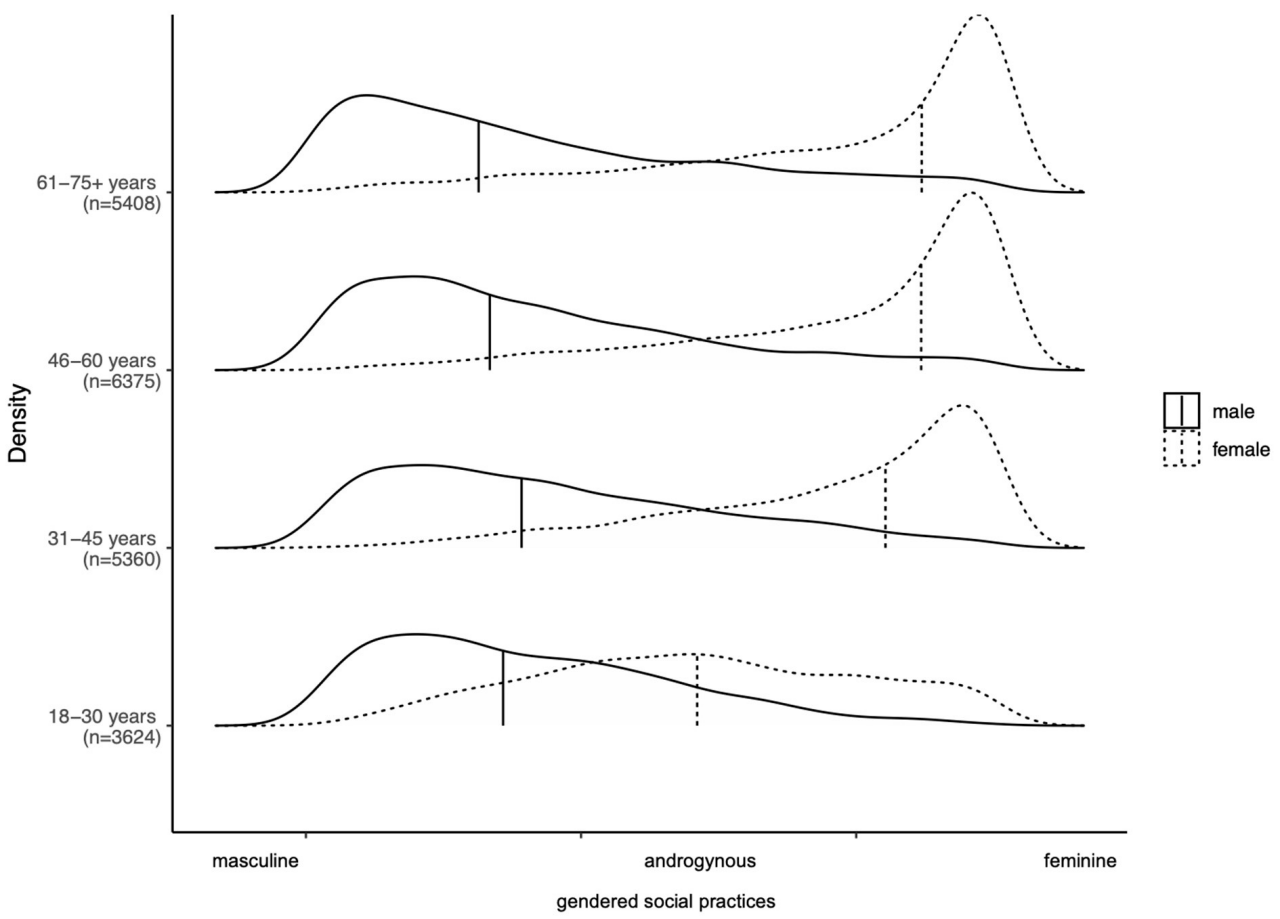
Gendered social practices by age, SOEP, Germany, 2018 (*n* = 20,767). The vertical bars represent the median for females and males respectively. Authors' own elaboration. Socioeconomic panel, wave v35.

When comparing the distribution of gendered social practices among different sociodemographic groups, the differences between age groups were most pronounced compared to all other groupings examined in this analysis (Figure 3). The older the participants, the more the distribution of females and males was skewed towards the respective end of the continuum. The youngest age group showed a different pattern: especially females did not show highly pronounced feminine social practices but shifted towards a more balanced distribution between the extremes. The overlap between females and males in gendered social practices was larger than for all other age groups. For males, the distribution did not change considerably. For females and males residing in eastern Germany, the overall distribution was very similar but the feminine and masculine social practices were less pronounced compared to females and males residing in western Germany (Supplementary material 5). We did not observe clear changes in patterns of social gendered practices by formal educational attainment or pre-government household income (Supplementary material 5).

In populations with immigration experience, we observed a different pattern (Supplementary material 5): feminine social practices in females became less pronounced, even shifting towards masculine practices. The distribution of masculine gendered practices in males did not change. With regard to the region of origin, among citizens of Western European countries and asylum seekers, masculine gendered practices among males were not as accentuated as among persons from Eastern Europe, South-West Asia, the Middle East and other immigration groups. Comparing the year of immigration to Germany, those who immigrated within the past 10 years showed higher overlap between gendered social practices among females and males, and comparatively lower peaks at both ends of the continuum (which might be due to a higher proportion of people of young age). The gendered social practices among females and males with permanent and temporary residence status in Germany did not differ from the overall pattern in Figure 2. When further stratifying migrant (and non-migrant) groups by age (Supplementary material 5), the age-specific pattern with more balanced distributions of gendered social practices for females (and partially males) remained.

**Figure 4:**
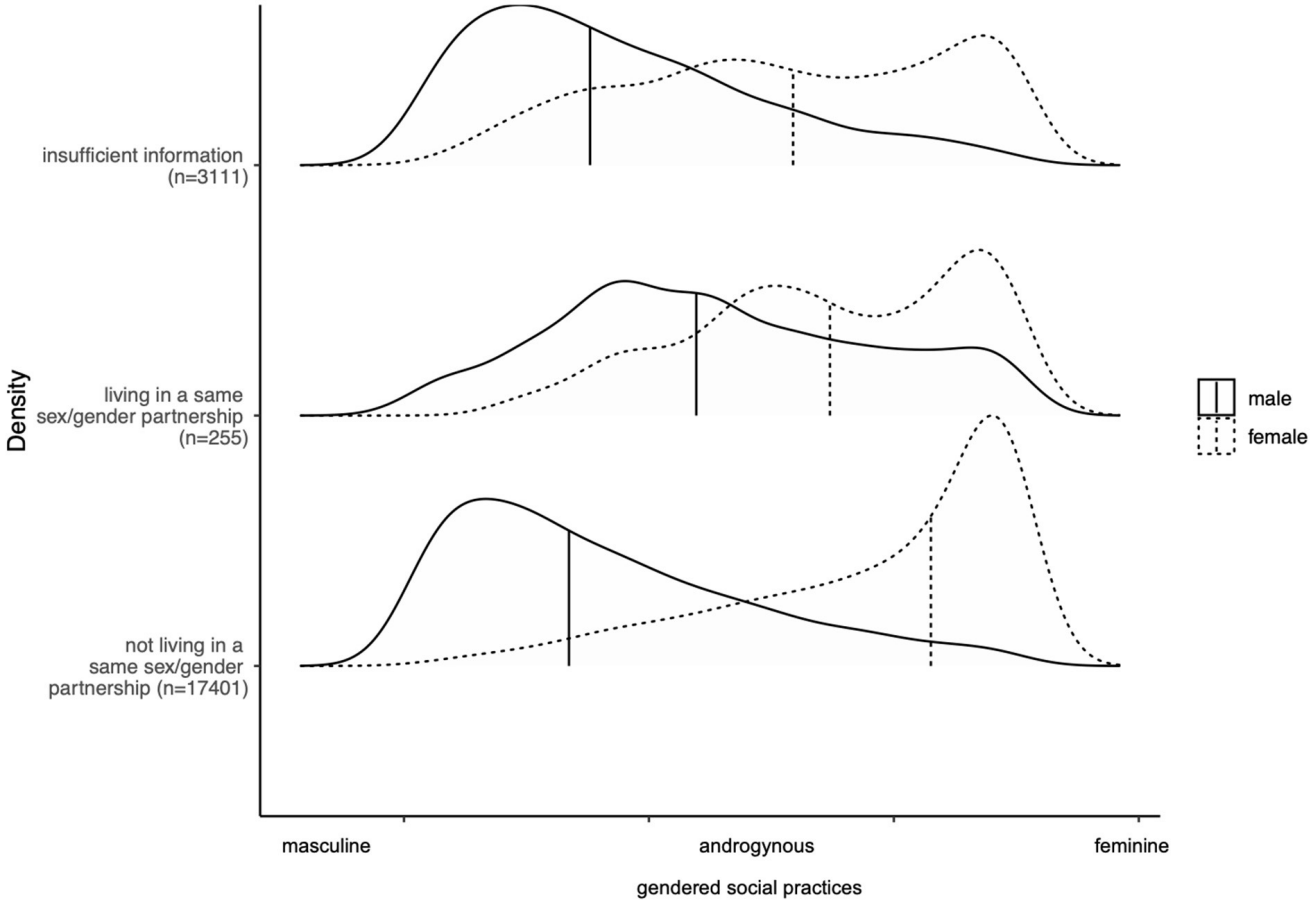
Gendered social practices by living in a same sex/gender partnership, SOEP, Germany, 2018 (*n* = 20,767). The vertical bars represent the median for females and males respectively. *Authors' own elaboration. Socioeconomic panel, wave v35*.

The distributions of masculine and feminine social practices also diverged from its overall pattern when comparing it in social groups defined by relationship and family status (Supplementary material 3). Females and males that did not have children, did not live with a partner or lived in a same sex/gender partnership showed greater overlap between feminine and masculine social practices among the sexes compared to those with organisations of family and partnership that are considered more traditional. Feminine social practices were still pronounced in females, but there was also a large proportion that shared rather balanced social practices. The same applied to males, especially for those who currently or usually live in a same-sex/gender partnership (Figure 4). For those who were currently or usually not living in a same-sex/gender partnership, having children and living with a partner, the distribution was very much similar to the overall distribution where we only differentiated by sex. We also compared the patterns of gendered social practices by parenthood and cohabitation with age and migration status (being born in Germany) and they remained stable in the additional subgroups (Supplementary material 6). This cross-comparison was not possible for the social groups describing same-sex/gender partnership due to the small sample size.

### Sensitivity analyses

When comparing the gender scores of the different regression models, the overall as well as the sex-specific distribution did not differ substantially, indicating that the exclusion of some variables did not affect the overall pattern of gendered social practices (Supplementary material 7).

Calculating the gender scores specifically for each social group, we saw that the variables defining gendered social practices might differ by social position (Supplementary material 7). For example, among individuals that were usually living in a same-sex/gender partnership and migrants originating from the Middle East, working in part-time jobs was not selected while it was highly significant in all other subsamples. Attitudes supporting gender equality among women, men and LGBTQI^*^ were less frequently represented in gender scores among migrant populations compared to non-migrant populations and also among individuals that were currently or usually living in a same-sex/gender partnership. Accordingly, the gender-related variables varied in their ability to discriminate between females and males in specific social groups. Yet, these subgroup analyses had varying sample sizes that were substantially smaller than the total sample which limited the generalisability of the findings.

The bootstrapping indicated marginal bias and low standard errors in coefficient estimates which indicates an acceptable model validity (Supplementary material 8).

## Discussion

Our explorative analyses showed that the distribution of gendered-social practices differed among social subgroups which empirically backed up the theoretical notion of gender being a relational, context-specific social construct, also at the intersection with social positions. We identified clear differences in gendered social practices among females and males by age, partnership status and parenthood, but less pronounced deviations for other demographics, such as formal education, income and migration status.

The gendered social practices showed highly skewed distributions either towards feminine social practices among females or masculine social practices among males respectively. This pattern was similar to previous scores, just like the variables selected to describe gendered practices or gendered norms: all encompassed data on household responsibilities, occupational status, level of empathy for other people, attitudes on gender norms and roles (9, 16, 18). Depending on the research interest, some variables were more prominent in other gender scores. For example, Smith et al. focused on labour force participation (9) and Fleming et al. addressed rather stereotyped behaviours and coping mechanisms in adolescents (e.g., frequency of crying, aggressive behaviours, physical activity, use of drugs) (16). A variable that wasn't considered in previous measures but identified in our multi-staged selection process included worries about terrorism. This constitutes an emerging field of research, as preliminary analyses suggest that gender is associated with differential vulnerability to the consequences of terror as well as differential awareness and perceptions (46).

Differences between masculine and feminine social practices diminished among young adults, as it has been observed in previous studies (47, 48). On the one hand, this could mirror societal change in gendered social practices with younger individuals showing less adherence to traditional gender roles. Surveys have shown that especially younger individuals agree less with traditional gender norms, and show higher diversity in terms of gender identity and increasing awareness and critique of gender hierarchies (49, 50). On the other hand, the variables included in the gender score might not mirror the gendered social practices of younger individuals as adequately as in middle and older age groups. Economic participation and household responsibilities could play a lesser role since for many of them their labour market debut is yet to come, they might not have their own household yet, and still live with their parents.

Migration status had less explanatory power for gendered social practices in our analyses. The differences between migrant and non-migrant populations as well as within migrant groups were rather marginal. This is not in line with evidence from the US showing higher adherence to traditional gender roles and norms among migrants compared to non-migrants (51). US migration history, however, differs from Germany's in terms of different groups entering the country, which could partly explain why patterns differed in our sample.

We observed differences in gendered practices in eastern and western Germany reflecting that work-family organisation is still substantially different due to historical political context. While in the communist state East Germany the “universal working” model was common, in West Germany the “breadwinner model” dominated. Their effects can still be seen today, e.g., in higher full-time employment rates among mothers of young children (52) and a smaller gender pay gap in eastern Germany compared to western Germany (even though the trends are converging) (53).

The variables our gender score is built on differentiated gendered social practices best for females and males with an organisation of family that is often perceived as “traditional”, i.e., individuals living with their partner, being parent, and not living in a same-sex/gender relationship. We saw a strong effect of settling in and starting a family, either with a partner or with children, on gendered social practices. Differences in gendered social practices became more pronounced among females and males when they lived with their partner or had at least one child which is consistent with studies showing a strong effect of marriage and parenthood e.g., on hours of housework, occupational status and income for women (but less for men) (54, 55). This could mirror the prevailing of heteronormative norms in our sample. It may also be an indication that gender is about relations to each other and not that much about individual characteristics. Family and household structures were highly important in constructing the gender score, which is consistent with previously constructed gender scores (9, 12, 16). These ultimately reflect political contexts and labour force expectations at the individual level. It is striking that in spite of the progress made in the last decades regarding women's participation in the work force and more egalitarian roles in the household (47), these remained the most relevant variables to describe gendered social practices in the largest German population survey in the year of 2018. Accordingly, the household and the labour market continue to be relevant contexts in forming, maintaining and reshaping gender (in)equality (56). These findings are consistent with European cross-country comparisons, which show that substantial gender disparities—usually to the detriment of women and in particular mothers – persist over the life course, both in employment and in private and family life (57).

Time-use data on housework and women's labour force participation have been significant variables of egalitarian gender roles and gender (in)equality of the past decades (47, 58, 59). Nordic countries have a longstanding formal commitment to gender equality and institutional support systems for working parents. Especially supportive family policies, such as formal childcare, parental leave, day care benefits and family allowance are known to help increase part- and full-time employment among women in Europe (60). Also, contextual, country-level gender ideologies were suggested to have an effect on the gendered division of housework (59). In comparison to other progressive European countries like the Nordic countries, the Netherlands or United Kingdom, institutional changes structuring family and labour market policies were introduced relatively late in the mid-2000s in Germany (37). The perseverance of the male breadwinner/female carer model is further supported by the diverging patterns of gendered social practices, especially for females, when comparing cohabitation status with a partner and having children.

To further examine the usefulness of gender scores, one could assess how gender-related variables vary in different political contexts and how they perform in cross-country comparisons compared to other gender measures. This could also expand to the small-area level to investigate whether smaller social contexts influenced by community actions, prevailing norms in the neighbourhood or school districts for adolescents show varying gendered social practices. Also, one could adapt this methodology to define a continuous scale for gender measures that are traditionally assessed as categorical variables, e.g., gender norms. Longitudinal studies are required to further examine whether the differences in gendered practices by age describe societal or individual-level changes over time. In addition, future data collection in health surveys needs to apply targeted recruitment strategies to include better SGM and allow for more differentiated analyses. Data on diverse gender identities can contribute to make gender measures, including the gender score, more inclusive (11). Moreover, we are in crucial need of measures that do not reproduce gender inequalities by using heteronormative and (albeit implicitly) sexist items to assess attitudes on gender norms and roles.

### Strengths & limitations

The construction of the gender score allowed for a theory-informed approach within the limits of a secondary data analysis. The gender diagnosticity method is a pragmatic approach to create a “local” (29) indicator of gender at the individual level representing gendered practise and performance that are shaped by societal gender norms in a given population. These characteristics, the relatively straight-forward methodological steps to create the gender score and the ability to operationalise a multidimensional gender concept in one variable have been considered particularly valuable and applicable by scholars engaged in gender analysis in social epidemiology and the broader quantitative population health research (8, 9, 11, 16, 18, 29). Still, capturing gendered practices based on the presence or absence of gendered dimension(s) in an individual has shortcomings that need to be taken into account when interpreting the findings. First, given the lack of a gold-standard measure for gender, this approach relies on sex-assigned at birth as the differential construct which has been identified as one of the major limitations and at times conflicting characteristics of this approach (61, 62). Second, the gendered dimensions in an individual might not be consistent (29). For example, a male participant shows affective relations that are considered to be more feminine in the given population (measured by worries of violence and terrorism), while the economic and power relations correspond to what is considered masculine (measured by the amount of full-time work). This shortcoming could be addressed by creating sub-scores of the different gender dimensions to account for the heterogenous nature of gender (in our case sub-scores for symbolic, economic and power as well as emotional relations, alternatives include to differentiate between professional and domestic gender etc.,) (11, 29, 63). Third, one must keep in mind that gendered dimensions might not solely be attributed to a gender mechanism, but could also be affected by other determinants, e.g., socioeconomic status (e.g., for attitudes on gender norms additional markers might be religious and political orient<ation) (29). Last, referring to the methodological aspects, the modelling approach including the conditional probabilities is typically used within a causal framework. When borrowed to create gender scores for secondary analysis in population health research, the research focus however shifts to identifying associations but not causal pathways. Taking into account these limitations, the gender score and similar approaches do not represent a holistic, universal operationalisation of (individual) gender. Most importantly they provide a pragmatic approach to create a multidimensional and context-specific measure of gender in a given population, particularly valuable in secondary data analysis (29).

We operationalised the gender score as a continuum and thereby tried to overcome a strict dichotomous approach, within the given limitations of data availability for biological sex. Accordingly, we were not able to capture the gendered experiences of intersex, gender-diverse or transgender people. Yet, alternatives to sex assigned at birth like attitudes towards gender norms or gendered behaviour didn't seem to be appropriate as they would only cover parts of the gender concepts. Also, the available items still reflected potential underlying gender bias: the SOEP does not provide gender-balanced questions on all topics covered, e.g., the survey only assesses attitudes towards working women but not working men. Although this approach does not succeed to overcome binary categories entirely, it can be measured with a continuous scale and represents a theoretically more profound approach by acknowledging that it is a context-specific construct. In addition, the relational gender theory understands gender and sex as distinct but interrelated concepts which makes a strict separation challenging (22).

Our analyses were explorative in nature and only provide cross-sectional data. In addition, the gender score has not been validated. Previous analyses however have shown good face validity in the sense that gender scores were associated but still distinct from sex and allowed to capture changes over time (9, 16). Our score used mostly self-reported data which might be subject to social desirability and recall bias. Especially for the time-use data, time diary data have shown to provide more precise and reliable estimates compared to self-reported data (64), which affects our estimates of weekday hours spent on housework and associated tasks. Also, we saw differences in the response rate of females and males to gender-related items included in the gender score, with males showing more missings than females (42% vs. 30% of all participants). Accordingly, our sample included more females than males which might indicate a reporting bias. Although our overall sample size was high, the sample selection might have introduced a selection bias which could impede the generalisability of the observed differences in gendered social practices by social position for males, people of older age and lower socioeconomic status.

### Conclusions

Our analysis shows differences in gendered practices across diverse social positions. The gender score appears to be a feasible approach in secondary data analysis and sensitive to these differential distributions of gendered practices among and between social positions. Our findings reiterate the need for gender measures that acknowledge gender as a context- and population specific social and relational construct and highlight the associated potential benefits for more precise gender analysis in epidemiology. We thereby add to the growing efforts to operationalise a comprehensive and specific gender measure in the field of public health.

Gender score measures (broadly based on the gender diagnosticity approach) covering similar data like time-use data, attitudes on gender norms and economic participation were associated with subjective well-being and symptoms like premature acute coronary syndrome (12, 17, 65). When gendered practices are included in representative surveys and health monitoring data, this could allow for a more precise differentiation of biological (sex) factors and socially constructed gendered practices that drive health behaviours, access to health care facilities or treatment decisions and ultimately health inequalities. One could then for example assess sex and gender as intermediate or mediating variables or examine the relative contribution of biological predictors and social dimensions to health differences (9). In addition, such measures could play a role in advancing gender analysis in clinical trials and treatment-decision making (11, 66).

Last, such measures could address the call for greater inclusion of theory in gender analysis in the field of epidemiology and public health in general. Drawing on social theories that are increasingly applied in health research, like the relational theory of gender by Connell (22) or intersectionality (67), contributes to increasing the gender-transformative potential of gender analysis in epidemiology. First, by identifying and monitoring markers driving heterogeneity between individuals related to sex/gender, we could more effectively contribute to dismantle these systems of privilege and oppression in local and national level policies and cultural beliefs. Second, by emphasising the relational and context-specific nature of gender—and at the same time continuously criticising the prevailing dichotomy and commingling with sex—a theory-driven approach could provide a more targeted examination of the prevailing power relations for the health issues under consideration.

## Data availability statement

The data analysed in this study is subject to the following licences/restrictions: The data is available upon reasonable request for scientific use only. Requests to access these datasets should be directed to German Institute for Economic Research (DIW Berlin), https://www.diw.de/en/diw_02.c.222829.en/access_and_ordering.html.

## Ethics statement

This study analysed secondary data. All data were anonymised prior to the authors receiving the data. The study protocols of SOEP-Core were approved by the German Institute for Economic Research and written informed consent was obtained from each subject.

## Author contributions

LW is primarily responsible for the idea, design and conduct of the research, and drafted the manuscript. OS, OR, and CM were involved in conceptualising the study design and the data analysis strategy. LW, OS, OR, and CM contributed to the final manuscript. All authors contributed to the article and approved the submitted version.

## Funding

This research has been conducted in the Gender Epidemiology Junior Research Group, funded by Bielefeld University. It received no specific grant from any funding agency in the public, commercial or not-for-profit sectors. Work on othering coordinated by OR is funded by German Research Foundation (DFG), RA 889/9-1 OTHER, as part of the Research Group PH-LENS (FOR 2928). We acknowledge the financial support of the German Research Foundation (DFG) and the Open Access Publication Fund of Bielefeld University for the article processing charge.

## Conflict of interest

The authors declare that the research was conducted in the absence of any commercial or financial relationships that could be construed as a potential conflict of interest.

## Publisher's note

All claims expressed in this article are solely those of the authors and do not necessarily represent those of their affiliated organizations, or those of the publisher, the editors and the reviewers. Any product that may be evaluated in this article, or claim that may be made by its manufacturer, is not guaranteed or endorsed by the publisher.
