# Supplementary material for: Development of a gender score in a representative German population sample and its association with diverse social positions
Source: Front Epidemiol. 2022 Aug 24;2:914819. doi: 10.3389/fepid.2022.914819 (PMC10910995; doi:10.3389/fepid.2022.914819)
Supplement: Supplementary file 1 [file Table_1.DOCX]

Supplementary Material 1 – Demographic differences between the sample and cases with missings excluded from the analysis

**Table 1** Demographics for sample with valid cases (included in the analysis) and cases with missing values for at least of the gender-related variables included in the genderscore (excluded from the analysis), SOEP, 2018, Germany

|  |  | **Sample** | | | | **Cases with missings**  (excluded from analysis) | | | | | p-value (Chi^2^-Test) |
| --- | --- | --- | --- | --- | --- | --- | --- | --- | --- | --- | --- |
|  |  | n | valid % | missings | | n | valid % | | missings | |  |
|  |  |  |  | n | % |  |  | | n | % |  |
| **Sociodemographics** | **Sex assigned at birth** |  |  |  |  |  |  | |  |  |  |
|  | male | 8902 | 42.87 | 0 | 0.00 | 3221 | 59.35 | 0 | | 0.00 | **<0.001** |
|  | female | 11865 | 57.13 |  |  | 2206 | 40.65 |  | |  |  |
|  | **Age** |  |  |  |  |  |  |  | |  |  |
|  | 18-30 years | 3624 | 17.45 |  |  | 1181 | 21.76 | 0 | | 0.00 | **<0.001** |
|  | 31-45 years | 5360 | 25.81 |  |  | 1200 | 22.11 |  | |  |  |
|  | 46-60 years | 6375 | 30.70 |  |  | 1493 | 27.51 |  | |  |  |
|  | 61-75+ years | 5408 | 26.04 |  |  | 1553 | 28.62 |  | |  |  |
|  | **Educational attainment (CASMIN classification)** |  |  |  |  |  |  |  | |  |  |
|  | low educational attainment | 6122 | 30.64 | 0 | 0.00 | 1788 | 35.55 | 397 | | 7.32 | **<0.001** |
|  | middle educational attainment | 8764 | 43.86 |  |  | 1946 | 38.69 |  | |  |  |
|  | high educational attainment | 5096 | 25.50 |  |  | 1296 | 25.77 |  | |  |  |
|  | **Pre-government household income** |  |  |  |  |  |  |  | |  |  |
|  | lowest income quintil | 2650 | 12.76 | 0 | 0.00 | 981 | 18.25 | 51 | | 0.94 | **<0.001** |
|  | middle income quintiles | 13394 | 64.50 |  |  | 3234 | 60.16 |  | |  |  |
|  | highest income quintil | 4723 | 22.74 |  |  | 1161 | 21.60 |  | |  |  |
|  |  |  |  |  |  |  |  |  | |  |  |
|  | **Region of Residence** |  |  |  |  |  |  |  | |  |  |
|  | West-Germany | 15875 | 76.44 | 0 | 0.00 | 4121 | 75.94 | 0 | | 0.00 | 0.443 |
|  | East-Germany | 4892 | 23.56 |  |  | 1306 | 24.06 |  | |  |  |
| **Migration status** | **Born in Germany** |  |  |  |  |  |  |  | |  |  |
|  | born in Germany or immigr.<1950 | 17359 | 83.59 | 0 | 0.00 | 4044 | 74.52 |  | |  | **<0.001** |
|  | not born in Germany | 3408 | 16.41 |  |  | 1383 | 25.48 |  | |  |  |
|  | **Migration background** |  |  |  |  |  |  |  | |  |  |
|  | no migration background | 15815 | 76.15 | 0 | 0.00 | 3664 | 67.51 |  | |  | **<0.001** |
|  | direct migration background | 3408 | 16.41 |  |  | 1383 | 25.48 |  | |  |  |
|  | indirect migration background | 1544 | 7.43 |  |  | 380 | 7.00 |  | |  |  |
|  | **Country of origin (including migrants and non-migrants)** |  |  |  |  |  |  |  | |  |  |
|  | Germany | 17359 | 91.10 |  |  | 4044 | 89.69 |  | |  |  |
|  | Poland | 463 | 2.43 | 1712 | 8.24 | 90 | 2.00 | 918 | | 16.92 | **<0.001** |
|  | Russia | 382 | 2.00 |  |  | 111 | 2.46 |  | |  |  |
|  | Kazakhstan | 330 | 1.73 |  |  | 93 | 2.06 |  | |  |  |
|  | Turkey | 271 | 1.42 |  |  | 107 | 2.37 |  | |  |  |
|  | Rumania | 250 | 1.31 |  |  | 64 | 1.42 |  | |  |  |
|  | **Region of origin (including migrants and non-migrants)** |  |  |  |  |  |  |  | |  |  |
|  | Germany | 17359 | 85.08 |  |  | 4044 | 77.68 |  | |  |  |
|  | Eastern Europe | 1696 | 8.31 | 365 | 1.76 | 478 | 9.18 | 221 | | 4.07 | **<0.001** |
|  | Western Europe | 455 | 2.23 |  |  | 99 | 1.90 |  | |  |  |
|  | Central Asia | 410 | 2.01 |  |  | 180 | 3.46 |  | |  |  |
|  | Middle East | 482 | 2.36 |  |  | 405 | 7.78 |  | |  |  |
|  | **Year of immigration** |  |  |  |  |  |  |  | |  |  |
|  | before 2009 | 2576 | 76.74 | 17410 | 83.83 | 749 | 55.20 | 4070 | | 75.00 | **<0.001** |
|  | 2009 to 2018 | 781 | 23.26 |  |  | 608 | 44.80 |  | |  |  |
|  | **Immigration group** |  |  |  |  |  |  |  | |  |  |
|  | Person of German descent from Eastern Europe | 936 | 32.30 | 17869 | 86.05 | 258 | 21.27 | 4214 | | 77.65 | **<0.001** |
|  | German who lived abroad | 80 | 2.76 |  |  | 19 | 1.57 |  | |  |  |
|  | Citizen of EU country (up to 2009 EC) | 759 | 26.19 |  |  | 187 | 15.42 |  | |  |  |
|  | Asylum seeker, refugee | 384 | 13.25 |  |  | 553 | 45.59 |  | |  |  |
|  | Other foreigner | 739 | 25.50 |  |  | 196 | 16.16 |  | |  |  |
|  | **Residence status** |  |  |  |  |  |  |  | |  |  |
|  | Unlimited | 998 | 68.92 | 19319 | 93.03 | 300 | 37.97 | 4637 | | 85.44 | **<0.001** |
|  | Temporary | 450 | 31.08 |  |  | 490 | 62.03 |  | |  |  |
| **Partnership and parenthood** | **Biological parenthood** |  |  |  |  |  |  |  | |  |  |
|  | childless | 5966 | 28.73 | 0 | 0.00 | 1824 | 33.61 |  | |  | **<0.001** |
|  | parent | 14801 | 71.27 |  |  | 3603 | 66.39 |  | |  |  |
|  | **Living with a partner** |  |  |  |  |  |  |  | |  |  |
|  | Single or not living with their partner | 8993 | 43.43 | 62 | 0.30 | 2364 | 43.72 | 20 | | 0.37 | 0.716 |
|  | living with a partner | 11712 | 56.57 |  |  | 3043 | 56.28 |  | |  |  |
|  | **Usually or currently living with a same sex/gender partner** |  |  |  |  |  |  |  | |  |  |
|  | no | 17401 | 83.79 | 0 | 0.00 | 4336 | 79.90 |  | |  | **<0.001** |
|  | yes | 255 | 1.23 |  |  | 53 | 0.98 |  | |  |  |
|  | insufficient information | 3111 | 14.98 |  |  | 1038 | 19.13 |  | |  |  |
