# Supplementary material for: Development of a gender score in a representative German population sample and its association with diverse social positions
Source: Front Epidemiol. 2022 Aug 24;2:914819. doi: 10.3389/fepid.2022.914819 (PMC10910995; doi:10.3389/fepid.2022.914819)
Supplement: Supplementary file 3 [file Table_3.DOCX]

Supplementary Material: R Code

##created by Lisa Wandschneider, last updated July 2022

##This code is prepared to replicate the analysis conducted for the paper

##IMPORT DATA

#bip = individual questionnaire 2018

#bioimmig = generated biographical information

#pl = individual questionnaire long

#pequiv =cross-national equivalent file long

#pgen = generated individual data

#ppathl = individual tracking file

library("foreign")

library("tidyverse")

library("dplyr")

#GENERATED DATA

#HEALTH

health <- read.dta("~/Desktop/SOEP Daten/Originaldatensatz/cs-transfer/Stata/health.dta")

glimpse(health)

#extract the variables needed, drop the others

keep <- c("hid", "pid", "syear", "hhnr", "persnr", "cid",

"mcs", "pcs", "bmi")

healths <- health[keep]

save(healths, file="healths.Rda")

#BIOIMMIG

bioimmig <- read.dta("~/Desktop/SOEP Daten/Originaldatensatz/cs-transfer/Stata/bioimmig.dta")

glimpse(bioimmig)

#extract the variables needed, drop the others

keep <- c("hid", "pid", "syear", "hhnr", "persnr", "bifamcl", "cid",

"bifamc", "biwfam", "biresper", "biimgrp")

bioimmigs <- bioimmig[keep]

save(bioimmigs, file="bioimmigs.Rda")

#PEQUIV

pequiv <- read.dta("~/Desktop/SOEP Daten/Originaldatensatz/cs-transfer/Stata/pequiv.dta")

glimpse(pequiv)

#extract the variables needed, drop the others

keep <- c("hid", "pid", "syear", "i11210", "e11102", "cid",

"e11201", "d11101", "l11101", "i11201", "d11104", "h11111",

"i11202", "m11125", "e11104", "i11203", "m11126", "h11101")

pequivs <- pequiv[keep]

save(pequivs, file="pequivs.Rda")

#PGEN

pgen <- read.dta("~/Desktop/SOEP Daten/Originaldatensatz/cs-transfer/Stata/pgen.dta")

glimpse(pgen)

#extract the variables needed, drop the others

keep <- c("hid", "pid", "syear", "pgcasmin", "cid",

"pgexppt", "pglabgro", "pgnation", "pglfs", "pglabnet",

"pgtatzeit", "pgemplst", "pgexpft", "pgisced11")

pgens <- pgen[keep]

save(pgens, file="pgens.Rda")

#PPATHL

ppathl <- read.dta("~/Desktop/SOEP Daten/Originaldatensatz/cs-transfer/Stata/ppathl.dta")

glimpse(ppathl)

#extract the variables needed, drop the others

keep <- c("hid", "pid", "syear", "persnr", "arefback", "cid",

"germborn", "migback", "miginfo", "sex", "immiyearinfo",

"sexorinfo", "arefinfo", "corigin", "gebmonat", "immiyear",

"sexor", "gebmoval", "germborninfo", "corigininfo")

ppathls <- ppathl[keep]

save(ppathls, file="ppathls.Rda")

#PL

pl <- read.dta("~/Desktop/SOEP Daten/Originaldatensatz/cs-transfer/Stata/pl.dta")

glimpse(pl)

#extract the variables needed, drop the others

keep <- c("hid", "pid", "syear", "cid",

"plh0173", "plh0180", "plj0023", "pli0022_h", "ple0036",

"ple0014", "ple0023", "pli0051", "pli0010", "ple0015",

"plh0181", "ple0027", "ple0028", "plh0042", "plh0178",

"plh0035", "plh0033", "plh0175", "ple0020", "ple0029",

"plh0179", "pli0019_h", "ple0011",

"pli0046", "ple0004", "ple0008", "pli0044_h", "ple0005",

"plj0022", "ple0026", "ple0032", "ple0035", "plh0176",

"pli0043_h", "plj0175", "pli0012_h", "ple0021", "plh0171",

"plh0335", "ple0034", "ple0017", "plh0032", "ple0030",

"plb0022_h", "plh0038", "pli0036", "plh0174", "ple0033",

"ple0031", "ple0018", "ple0016", "pli0016_h", "plh0182",

"plh0177", "ple0022", "ple0019", "plb0219", "ple0013",

"plh0336", "ple0012", "plh0040",

"plb0018", "plb0037_h", "plb0241_h", "plc0010", "pld0062_v2",

"pld0068_v2", "pld0083_v2", "plh0033", "plh0033", "plh0036",

"plh0037", "plh0038", "plh0039", "plh0040", "plh0040",

"plh0162", "plh0171", "plh0172", "plh0174", "plh0176", "plh0178",

"plh0184", "plh0185", "plh0186", "plh0187", "plh0189", "plh0193",

"plh0195", "plh0204_h", "plh0335", "pli0043_h", "pli0046",

"pli0049_h", "pli0051", "pli0162", "plj0566", "plj0567")

pls <- pl[keep]

save(pls, file="pls.Rda")

#BIOBIRTH

biobirth <- read.dta("/Users/lisawandschneider/Desktop/SOEP Daten/Originaldatensatz/cs-transfer/Stata/biobirth.dta")

keep <- c("hid", "pid", "gebjahr", "cid",

"biovalid", "sumkids", "biokids")

biobirths <- biobirth[keep]

save(biobirths, file="biobirths.Rda")

#RAW DATASETS

#BIP

bip <- read.dta("~/Desktop/SOEP Daten/Originaldatensatz/cs-transfer/Stata/raw/bip.dta")

glimpse(bip)

#extract the variables needed, drop the others

keep <- c("hid", "pid", "syear", "cid",

"bip_197_02", "bip_197_06", "bip_197_05", "bip_197_04",

"bip_197_07", "bip_197_01", "bip_197_08", "bip_197_03",

"bip_148", "bip_43", "bip_19")

bips <- bip[keep]

save(bips, file="bips.Rda")

#bipequiv

bipequiv <- read.dta("/Users/lisawandschneider/Desktop/SOEP Daten/Originaldatensatz/cs-transfer/Stata/raw/bipequiv.dta")

keep <- c("hid", "pid", "syear",

"i1110118")

bipequivs <- bipequiv[keep]

save(bipequivs, file="bipequivs.Rda")

#bipgen

bipgen <- read.dta("/Users/lisawandschneider/Desktop/SOEP Daten/Originaldatensatz/cs-transfer/Stata/raw/bipgen.dta")

glimpse(bipgen)

#extract the variables needed, drop the others

keep <- c("hid", "pid", "cid", "syear",

"bifamstd")

bipgens <- bipgen[keep]

save(bipgens, file="bipgens.Rda")

#pbrutto

pbrutto <- read.dta("/Users/lisawandschneider/Desktop/SOEP Daten/Originaldatensatz/cs-transfer/Stata/pbrutto.dta")

glimpse(pbrutto)

#extract the variables needed, drop the others

keep <- c("hid", "pid", "cid", "syear",

"stell_v1", "stell_v2", "stell_h")

pbruttos <- pbrutto[keep]

save(pbruttos, file="pbruttos.Rda")

nrow(subset(pbrutto, syear=="2018"))

pbrutto2018 <- subset(pbrutto, syear=="2018")

glimpse(pbrutto2018)

table(pbrutto2018$stell_v1, exclude = NULL)

table(pbrutto2018$stell_v2, exclude = NULL)

table(pbrutto2018$stell_h, exclude = NULL)

df <- pbrutto2018

#child in the household: biological child, stepchild, adopted, foster child

glimpse(df)

child <- c("[21] leibliches Kind",

"[22] Stiefkind", "[23] Adoptivkind",

"[24] Pflegekind")

df <- mutate(df, hchild = ifelse(stell_v2 %in% child, 1, 0))

table(df$hchild)

table(df$stell_v2)

#consistent results

#even larger definition: incl. grandchild, great grandchild

child1 <- c("[21] leibliches Kind",

"[22] Stiefkind", "[23] Adoptivkind",

"[24] Pflegekind", "[25] Enkelkind", "[26] Urenkelkind")

df <- mutate(df, hchild1 = ifelse(stell_v2 %in% child1, 1, 0))

table(df$hchild1)

table(df$stell_v2)

#consistent, adds about 150 children

#create household level data

#households with at least one individual with hchild == 1 should be scored 1, all other 0

#new dataset with hid and #children in the hh

test <- aggregate(df$hchild, by=list(Category=df$hid), FUN=sum)

test1 <- aggregate(df$hchild1, by=list(Category=df$hid), FUN=sum)

#merge the two new hhdata sets

df_1 <- merge(test, test1, by = "Category", all.x = TRUE, all.y = TRUE)

#recode variables

#category to hid

df_1$hid <- df_1$Category

glimpse(df_1)

table(df_1$x.x)

table(df_1$x.y)

#children

df_1 <- mutate(df_1, hhchild = ifelse(x.x == 0, 0, 1))

#all children incl grandchildren

#x.x and x.y to hhchild

df_1 <- df_1 %>% mutate(hhchild_ext = x.x + x.y)

df_1 <- mutate(df_1, hhchild_extr = ifelse(hhchild_ext == 0, 0, 1))

glimpse(df_1)

table(df_1$hhchild)

table(df_1$hhchild_extr)

##merge with pbrutto to get back to individual level

parent <- merge(df_2, pbrutto2018, by = "hid", all = TRUE)

glimpse(parent)

#recode to individual level

#code as parent if: person = head of household and hhchild == 1

# OR person == partner of household & hhchild ==1

table(parent$stell_v2)

head_partner <- c("[0] Haushaltsvorstand", "[11] Ehegatte/-in",

"[12] Gleichgeschlechtliche/r Partner/in lt. Lebenspartnergesetz",

"[13] Lebenspartner/-in")

parent <- mutate(parent, socp = ifelse(stell_v2 %in% head_partner

& hhchild == 1, 1, 0) )

glimpse(parent)

table(parent$socp)

#extract the variables needed, drop the others

keep <- c("hid", "pid", "hhchild", "hhchild_extr", "socp")

parents <- parent[keep]

save(parents, file="parents.Rda")

#----------------------------------------------------------#

library(dplyr)

library(tidyr)

library(ggplot2)

#load datasets

load("/Users/lisawandschneider/sciebo2/meinGendEpi - Work in Progress/5_SOEP_composite gender measure/SOEP_compm/pequivs.Rda")

load("/Users/lisawandschneider/sciebo2/meinGendEpi - Work in Progress/5_SOEP_composite gender measure/SOEP_compm/ppathls.Rda")

load("/Users/lisawandschneider/sciebo2/meinGendEpi - Work in Progress/5_SOEP_composite gender measure/SOEP_compm/pgens.Rda")

load("/Users/lisawandschneider/sciebo2/meinGendEpi - Work in Progress/5_SOEP_composite gender measure/SOEP_compm/bips.Rda")

load("/Users/lisawandschneider/sciebo2/meinGendEpi - Work in Progress/5_SOEP_composite gender measure/SOEP_compm/healths.Rda")

load("/Users/lisawandschneider/sciebo2/meinGendEpi - Work in Progress/5_SOEP_composite gender measure/SOEP_compm/bioimmigs.Rda")

load("/Users/lisawandschneider/sciebo2/meinGendEpi - Work in Progress/5_SOEP_composite gender measure/SOEP_compm/bipequivs.Rda")

load("/Users/lisawandschneider/sciebo2/meinGendEpi - Work in Progress/5_SOEP_composite gender measure/SOEP_compm/pls.Rda")

load("/Users/lisawandschneider/sciebo2/meinGendEpi - Work in Progress/5_SOEP_composite gender measure/SOEP_compm/biobirths.Rda")

load("/Users/lisawandschneider/sciebo2/meinGendEpi - Work in Progress/5_SOEP_composite gender measure/SOEP_compm/bipgens.Rda")

load("/Users/lisawandschneider/sciebo2/meinGendEpi - Work in Progress/5_SOEP_composite gender measure/SOEP parenthood/parents.Rda")

#MERGE DATA

#merge two data frames by ID

#start with bip - 2018 questionnaire

#check merge: n vergleichen, missings in pid zählen

#all = True -> keep all rows from both dataframes

#To include all the rows of your data frame x and only those from y that match, specify all.x=TRUE.

#merge0 <- merge(bips, kidlongs, by = c("pid", "cid"), all.x = TRUE)

#glimpse(merge0)

#merge0$syear <- merge0$syear.x

#merge1 <- merge(merge0, pls, by = c("pid", "syear"), all.x = TRUE)

merge1 <- merge(bips, pls, by = c("pid", "syear"), all.x = TRUE)

merge2 <- merge(merge1, pequivs, by = c("pid", "syear"), all.x = TRUE)

merge3 <- merge(merge2, bipequivs, by = c("pid", "syear"), all.x = TRUE)

merge4 <- merge(merge3, ppathls, by = c("pid", "syear"), all.x = TRUE)

merge5 <- merge(merge4, pgens, by = c("pid", "syear"), all.x = TRUE)

merge6 <- merge(merge5, healths, by = c("pid", "syear"), all.x = TRUE)

merge6a <- merge(merge6, bipgens, by = c("pid", "cid"), all.x = TRUE)

merge7 <- merge(merge6a, biobirths, by = c("pid", "cid"), all.x = TRUE)

glimpse(merge7)

merge7$syear <- merge7$syear.x

#to add bioimmigs without minimising the 2018 data

#select only 2018 in bioimmigs

bioimmig18 <- subset(bioimmigs, syear=="2018")

merge8 <- merge(merge7, bioimmig18, by = c("pid", "syear"), all.x = TRUE)

glimpse(merge8)

df_raw <- merge8

nrow(subset(df_raw, syear=="2018"))

#clean merge datasets

rm(merge1, merge2, merge3, merge4, merge5, merge6, merge7, merge8)

rm(pequivs, pls, ppathls, bioimmigs, bipequivs, healths, pgens, biobirths, bioimmig18, bips)

#------------------------------------------------------------#

#remove duplicate colnames

colnames(df_raw) <- make.unique(names(df_raw))

glimpse(df_raw)

df_raw$cid <- df_raw$cid.x.3

#clean duplicates

df_raw$cid.x <- NULL

df_raw$cid.x.1 <- NULL

df_raw$cid.x.2 <- NULL

df_raw$cid.y <- NULL

df_raw$cid.y.1 <- NULL

df_raw$cid.y.2 <- NULL

df_raw$cid.y.3 <- NULL

df_raw$syear.x <- NULL

df_raw$syear.y <- NULL

df_raw$cid.x.3 <- NULL

#remove duplicate cases

df_raw <- unique(df_raw)

save(df_raw, file="df_raw.Rda")

#------------------------------------------------------------#

#replace all negative values with NA

#numeric variables

dfna <- df_raw %>% mutate_all(list(~ replace(., .<0, NA)))

warnings()

#factor variables

dfna[] <- lapply(dfna, function(x) {

is.na(levels(x)) <- levels(x) == "[-1] keine Angabe"

x

})

dfna[] <- lapply(dfna, function(x) {

is.na(levels(x)) <- levels(x) == "[-1] Keine Angabe"

x

})

dfna[] <- lapply(dfna, function(x) {

is.na(levels(x)) <- levels(x) == "[-2] trifft nicht zu"

x

})

dfna[] <- lapply(dfna, function(x) {

is.na(levels(x)) <- levels(x) == "[-2] Trifft nicht zu"

x

})

dfna[] <- lapply(dfna, function(x) {

is.na(levels(x)) <- levels(x) == "[-3] nicht valide"

x

})

dfna[] <- lapply(dfna, function(x) {

is.na(levels(x)) <- levels(x) == "[-3] Nicht valide"

x

})

dfna[] <- lapply(dfna, function(x) {

is.na(levels(x)) <- levels(x) == "[-4] Unzulaessige Mehrfachantwort"

x

})

dfna[] <- lapply(dfna, function(x) {

is.na(levels(x)) <- levels(x) == "[-4] unzulaessige Mehrfachantwort"

x

})

dfna[] <- lapply(dfna, function(x) {

is.na(levels(x)) <- levels(x) == "[-5] In Fragebogenversion nicht enthalten"

x

})

dfna[] <- lapply(dfna, function(x) {

is.na(levels(x)) <- levels(x) == "[-5] in Fragebogenversion nicht enthalten"

x

})

dfna[] <- lapply(dfna, function(x) {

is.na(levels(x)) <- levels(x) == "[-6] Fragebogenversion mit geaenderter Filterfuehrung"

x

})

dfna[] <- lapply(dfna, function(x) {

is.na(levels(x)) <- levels(x) == "[-8] Frage in diesem Jahr nicht Teil des Frageprogramms"

x

})

dfna[] <- lapply(dfna, function(x) {

is.na(levels(x)) <- levels(x) == "[-8] Frage in diesem Jahr nicht Teil des Frageprograms"

x

})

save(dfna, file="dfna.Rda")

#--------------------------------------------------------------#

##RECODE VARIABLES

dfs <- dfna

library("sjmisc")

library("dplyr")

#SOCIODEMOGRAPHICS

#SEX sex

table(dfs$sex, exclude = NULL)

dfs$sexr <- as.character(dfs$sex)

dfs$sexr <- dplyr::recode_factor(dfs$sexr, '[1] maennlich' = "male", '[2] weiblich' = "female",

as.default = NA_character_)

table(dfs$sexr, exclude = NULL)

class(dfs$sexr)

#AGE d11101

library("ggplot2")

mean(dfs$d11101, na.rm = TRUE)

median(dfs$d11101, na.rm = TRUE)

sd(dfs$d11101, na.rm=TRUE)

min(dfs$d11101, na.rm = TRUE)

max(dfs$d11101, na.rm = TRUE)

p <- dfs %>% ggplot(aes(d11101)) + geom_histogram(binwidth = 1)

p

dfs$age <- as.numeric(dfs$d11101)

class(dfs$age)

descr(dfs$age)

#age groups 18-30, 30-45, 46-60, 60-75+

dfs <- mutate(dfs, agec = ifelse(d11101 %in% 18:30, 0,

ifelse(d11101 %in% 31:45, 1,

ifelse(d11101 %in% 46:60, 2,

ifelse(d11101 %in% 61:120, 3, NA)))))

dfs$agecr <- as.factor(dfs$agec)

dfs$agecr <- dplyr::recode_factor(dfs$agecr, '0' = "18-30 years",

'1' = "31-45 years",

'2' = "46-60 years",

'3' = "61-75+ years",

.default = NA_character_)

table(dfs$agecr, exclude = NULL)

#EDUCATIONAL ATTAINMENT

#CASMIN pgcasmin

#The high educa-tion group is defined as all persons with low or high tertiary degrees, the medium education group consists of those with a vocational degree (intermediate general qualification, intermediate vocational, general matu- rity certificate, vocational maturity certificate) and the lowest education group includes all respondents with inadequately completed general education, general elementary education or basic vocational qualification.

table(dfs$pgcasmin)

dfs$edu <- dplyr::recode(dfs$pgcasmin, '[0] (0) in school' = 0,

'[1] (1a) inadequately completed' = 1,

'[2] (1b) general elementary school' = 2,

'[3] (1c) basic vocational qualification' = 3,

'[4] (2b) intermediate general qualification' = 4,

'[5] (2a) intermediate vocational' = 5,

'[6] (2c_gen) general maturity certificate' = 6,

'[7] (2c_voc) vocational maturity certificate' = 7,

'[8] (3a) lower tertiary education' = 8,

'[9] (3b) higher tertiary education' = 9)

dfs <- mutate(dfs, educ = ifelse(edu %in% 0:3, 0,

ifelse(edu %in% 4:7, 1,

ifelse(edu %in% 8:9, 2, NA))))

dfs$edur <- dplyr::recode_factor(dfs$educ, '0' = "low educational attainment",

'1' = "middle educational attainment",

'2' = "high educational attainment",

.default = NA_character_)

table(dfs$edur, exclude = NULL)

table(dfs$pgcasmin, exclude = NULL)

#HOUSEHOLD INCOME

#Household Pre-Government Income i1110118 -> need to extract it from pequiv

#categorize pcs into quintiles

library(DescTools)

quantile(dfs$i1110118, probs = seq(0, 1, 1/5), na.rm = TRUE)

dfs$quint_inc <- CutQ(dfs$i1110118, breaks = quantile(dfs$i1110118, seq(0, 1, by = 0.2), na.rm = TRUE))

table(dfs$quint_inc, exclude = NULL)

dfs$inc <- dplyr::recode(dfs$quint_inc,

'Q1' = 1,

'Q2' = 2,

'Q3' = 3,

'Q4' = 4,

'Q5' = 5)

table(dfs$quint_inc)

table(dfs$inc)

dfs <- mutate(dfs, inc_c = ifelse(inc == 1, 0,

ifelse(inc %in% 2:4, 1,

ifelse(inc == 5, 2, NA))))

dfs$incr <- dplyr::recode_factor(dfs$inc_c, '0' = "low monthly household income",

'1' = "middle monthly household income",

'2' = "high monthly household income",

.default = NA_character_)

table(dfs$incr, exclude = NULL)

#STATE OF RESIDENCE l11101

table(dfs$l11101, exclude = NULL)

dfs$germstate <- as.character(dfs$l11101)

dfs$germstate <- dplyr::recode_factor(dfs$germstate,

'[1] Scpequiveswig-Holstein 1' = "Schleswig-Holstein",

'[2] Hamburg 2' = "Hamburg",

'[3] Lower Saxony 3' = "Lower Saxony",

'[4] Bremen 4' = "Bremen",

'[5] North-Rhine-Westfalia 5' = "North-Rhine-Westfalia",

'[6] Hessen 6' = "Hessen",

'[7] Rheinland-Pfalz 7' = "Rheinland-Pfalz",

'[8] Baden-Wuerttemberg 8' = "Baden-Wuerttemberg",

'[9] Bavaria 9' = "Bavaria",

'[10] Saarland 10' = "Saarland",

'[11] Berlin 11' = "Berlin",

'[12] Brandenburg 12' = "Brandenburg",

'[13] Mecklenburg-Vorpommern 13' = "Mecklenburg-Vorpommern",

'[14] Saxony 14' = "Saxony",

'[15] Saxony-Anhalt 15' = "Saxony-Anhalt",

'[16] Thuringia 16' = "Thuringia",

as.default = NA_character_)

table(dfs$germstate, exclude = NULL)

class(dfs$germstate)

frq(dfs$germstate)

#dichotomous variable: East vs. West

#east: Brandenburg, Sachsen, Sachsen-Anhalt, Mecklenburg, Thüringen

# 1 east, 0 west

dfs$east <- as.character(dfs$l11101)

dfs$east <- dplyr::recode(dfs$east, '[1] Scpequiveswig-Holstein 1' = 1,

'[2] Hamburg 2' = 2,

'[3] Lower Saxony 3' = 3,

'[4] Bremen 4' = 4,

'[5] North-Rhine-Westfalia 5' = 5,

'[6] Hessen 6' = 6,

'[7] Rheinland-Pfalz 7' = 7,

'[8] Baden-Wuerttemberg 8' = 8,

'[9] Bavaria 9' = 9,

'[10] Saarland 10' = 10,

'[11] Berlin 11' = 11,

'[12] Brandenburg 12' = 12,

'[13] Mecklenburg-Vorpommern 13' = 13,

'[14] Saxony 14' = 14,

'[15] Saxony-Anhalt 15' = 15,

'[16] Thuringia 16' = 16)

dfs <- mutate(dfs, eastr = ifelse(east %in% 11:16, 1,

ifelse(east %in% 1:10, 0, NA)))

table(dfs$eastr)

dfs$eastr <- as.factor(dfs$eastr)

dfs$eastr <- dplyr::recode_factor(dfs$eastr, '0' = "West", '1' = "East")

frq(dfs$eastr)

#sexual orientation

table(dfs$sexor)

dfs$sexor <- as.factor(dfs$sexor)

dfs$sexor <- dplyr::recode_factor(dfs$sexor, '[0] probably heterosexual' = "probably heterosexual",

'[1] probably bi/homosexual' = "probably bi/homosexual",

'[2] insufficient information' = "insufficient information")

table(dfs$sexor)

#PARENTHOOD

glimpse(dfs)

glimpse(parents)

dfs <- merge(dfs, parents, by = c("cid", "pid"), all.x = TRUE)

#social parenthood = socp

table(dfs$socp)

dfs$socpr <- recode_factor(dfs$socp, '0' = "no social children",

'1' = "at least 1 social child")

table(dfs$socpr)

#biological parenthood

table(dfs$sumkids, exclude = NULL) #total number of births

table(dfs$biokids, exclude = NULL) #number of births from biography

table(dfs$biovalid, exclude = NULL)

#sumkids = total number of kids born (identifies most parents, no missings)

dfs <- mutate(dfs, biop = ifelse(sumkids == 0, 0,

ifelse(sumkids %in% 1:17, 1, NA)))

dfs$biopr <- recode_factor(dfs$biop, '0' = "no biological children",

'1' = "at least 1 biological child")

table(dfs$biopr, exclude = NULL)

#merge social and biological parenthood, 3 categories

#parents cohabitating with children (social & biological),

#parents not cohabitating (biological parents not identified in social parenthood),

#neither biological nor social children

dfs <- mutate(dfs, parent_all = ifelse(biop == 1 & socp == 1, 2,

ifelse(biop == 1 & socp == 0, 1, 0)))

table(dfs$parent_all)

dfs$parent_allr <- recode_factor(dfs$parent_all, '0' = "no children",

'1' = "parents not cohabitating with child(ren)",

'2' = "parents cohabitating with child(ren)")

table(dfs$parent_allr)

table(dfs$biopr)

table(dfs$socpr)

#marital status

table(dfs$bifamstd)

single <- c("[2] Verheiratet, dauernd getrennt lebend", "[3] Ledig",

"[4] Geschieden / eingetragene gleichgeschlechtliche Partnerschaft aufgehoben",

"[5] Verwitwet / Lebenspartner/-in aus eingetragener gleichgeschlechtlicher Partner",

"[8] Eingetragene gleichgeschlechtliche Partnerschaft getrennt lebend")

partner <- c("[1] Verheiratet, mit Ehepartner zusammenlebend", "[6] Ehepartner im Ausland",

"[7] Eingetragene gleichgeschlechtliche Partnerschaft zusammenlebend")

livewpartner <- c("[1] Verheiratet, mit Ehepartner zusammenlebend",

"[7] Eingetragene gleichgeschlechtliche Partnerschaft zusammenlebend")

livapart <- c("[2] Verheiratet, dauernd getrennt lebend", "[3] Ledig", "[6] Ehepartner im Ausland",

"[4] Geschieden / eingetragene gleichgeschlechtliche Partnerschaft aufgehoben",

"[5] Verwitwet / Lebenspartner/-in aus eingetragener gleichgeschlechtlicher Partner",

"[8] Eingetragene gleichgeschlechtliche Partnerschaft getrennt lebend")

dfs <- mutate(dfs, single = ifelse(bifamstd %in% single, 0,

ifelse(bifamstd %in% partner, 1, NA)))

table(dfs$single, exclude = NULL)

dfs$single <- recode_factor(dfs$single, '0' = "single",

'1' = "in partnership")

dfs <- mutate(dfs, livwpart = ifelse(bifamstd %in% livapart, 0,

ifelse(bifamstd %in% livewpartner, 1, NA)))

table(dfs$livwpart, exclude = NULL)

dfs$livwpart <- recode_factor(dfs$livwpart, '0' = "single or not living with their partner",

'1' = "living with a partner")

#HEALTH VARIABLES

#OUTCOMES

#PCS

mean(dfs$pcs, na.rm=TRUE)

median(dfs$pcs, na.rm=TRUE)

sd(dfs$pcs, na.rm=TRUE)

descr(dfs$pcs)

p <- dfs %>% ggplot(aes(pcs)) + geom_histogram(binwidth = 1)

p

#categorize pcs into tertiales

quantile(dfs$pcs, probs = seq(0, 1, 1/3), na.rm = TRUE)

dfs$pcscat <- CutQ(dfs$pcs, breaks = quantile(dfs$pcs, seq(0, 1, by = 1/3), na.rm = TRUE))

table(dfs$pcscat, exclude = NULL)

dfs$pcscat <- dplyr::recode(dfs$pcscat,

'Q1' = 1,

'Q2' = 2,

'Q3' = 3)

table(dfs$pcscat)

dfs$pcscatr <- dplyr::recode_factor(dfs$pcscat, '1' = "low physical health",

'2' = "middle physical health",

'3' = "high physical health",

.default = NA_character_)

table(dfs$pcscatr)

dfs <- mutate(dfs, pcsdich = ifelse(pcscat %in% 1:2, 0,

ifelse(pcscat == 3, 1,NA)))

dfs$pcsdich <- dplyr::recode_factor(dfs$pcsdich, '0' = "low to middle physical health",

'1' = "high physical health",

.default = NA_character_)

table(dfs$pcsdich, exclude = NULL)

#MCS

mean(dfs$mcs, na.rm=TRUE)

median(dfs$mcs, na.rm=TRUE)

sd(dfs$mcs, na.rm=TRUE)

descr(dfs$mcs)

p <- dfs %>% ggplot(aes(mcs)) + geom_histogram(binwidth = 1)

p

#categorize mcs into tertiales

quantile(dfs$mcs, probs = seq(0, 1, 1/3), na.rm = TRUE)

dfs$mcscat <- CutQ(dfs$mcs, breaks = quantile(dfs$mcs, seq(0, 1, by = 1/3), na.rm = TRUE))

table(dfs$mcscat, exclude = NULL)

dfs$mcscat <- dplyr::recode(dfs$mcscat,

'Q1' = 1,

'Q2' = 2,

'Q3' = 3)

table(dfs$mcscat)

dfs$mcscatr <- dplyr::recode_factor(dfs$mcscat, '1' = "low mental health",

'2' = "middle mental health",

'3' = "high mental health",

.default = NA_character_)

table(dfs$mcscatr)

dfs <- mutate(dfs, mcsdich = ifelse(mcscat %in% 1:2, 0,

ifelse(mcscat == 3, 1,NA)))

dfs$mcsdich <- dplyr::recode_factor(dfs$mcsdich, '0' = "low to middle mental health",

'1' = "high mental health",

.default = NA_character_)

table(dfs$mcsdich, exclude = NULL)

#MIGRATION RELATED VARIABLES

#MIGRATION BACKGROUND migback

#SOEP classification

#Respondents were assigned to the MIGBACK categories based on country of birth (see GERMBORN): Being born in another country than Germany indicates, by definition, a direct migration background (2), while respondents born in Germany may have either no (1) or an indirect (3) migration background. Respondents whose parents had no migration background were assigned the code “(1) no migration background”, while respondents whose father or mother had a migration background were assigned the code “(3) indirect migration background”. Grandparental information were additionally used if information on mothers’ and fathers’ migration background were missing

table(dfs$migback, exclude = NULL)

dfs$migbackr <- as.character(dfs$migback)

dfs$migbackr <- dplyr::recode_factor(dfs$migbackr, '[1] kein Migrationshintergrund' = "no migration background",

'[2] direkter Migrationshintergrund' = "direct migration background",

'[3] indirekter Migrationshintergrund' = "indirect migration background",

as.default = NA_character_)

table(dfs$migbackr, exclude = NULL)

#migration background vs. no migration background

dfs <- mutate(dfs, migbackbin = ifelse(migbackr == "no migration background", 0, 1))

dfs$migbackbin <- dplyr::recode_factor(dfs$migbackbin, '0' = "No migration background",

'1' = "Migration background", as.default = NA_character_)

table(dfs$migbackbin)

#COUNTRY OF ORIGIN corigin corigin

table(dfs$corigin, exclude = NULL)

#too many countries, go for imgroup or germborn

sort(table(dfs$corigin),decreasing=T, exclude = NULL)

#biggest groups: Deutschland, Polen, Russland, Kasachstan, Tuerkei, Rumaenien

dfs <- mutate(dfs, corigin_big6 = ifelse(corigin == "[1] Deutschland", 1,

ifelse(corigin == "[22] Polen", 2,

ifelse(corigin == "[32] Russland", 3,

ifelse(corigin == "[74] Kasachstan", 4,

ifelse(corigin == "[2] Tuerkei", 5,

ifelse(corigin == "[21] Rumaenien", 6, NA)))))))

table(dfs$corigin_big6)

dfs$corigin_big6 <- recode_factor(dfs$corigin_big6, '1' = "Germany",

'2' = "Poland", '3' = "Russia", '4' = "Kazakhstan",

'5' = "Turkey", '6' = "Rumania",

as.default = NA_character_)

table(dfs$corigin_big6, exclude = NULL)

#by region

Germany <- c("[1] Deutschland", "[7] Ex-DDR(nur Herkunftsland)")

easteurope <- c("[3] Ex-Jugoslawien",

"[21] Rumaenien", "[22] Polen", "[26] Ungarn", "[29] Bulgarien",

"[31] Tschechien", "[32] Russland", "[75] Albanien",

"[78] Ukraine", "[140] Kosovo-Albaner",

"[165] Serbien", "[101] Estland",

"[103] Lettland", "[106] Montenegro", "[119] Kroatien",

"[120] Bosnien/Herzegowina", "[121] Makedonien", "[122] Slowenien",

"[123] Slowakei", "[132] Weissrussland", "[146] Litauen",

"[153] Freistaat Danzig", "[165] Serbien", "[168] Montenegro",

"[180] Bessarabien", "[222] Eastern Europe")

westeurope <- c("[4] Griechenland","[5] Italien", "[6] Spanien",

"[10] Oesterreich", "[11] Frankreich", "[12] Benelux", "[13] Daenemark",

"[14] Grossbritannien", "[15] Schweden", "[16] Norwegen", "[17] Finnland",

"[19] Schweiz", "[28] Portugal",

"[112] Malta",

"[62] Monaco", "[69] Liechtenstein", "[70] Island", "[71] Irland",

"[116] Luxemburg", "[117] Belgien", "[118] Niederlande")

Asia <- c("[23] Korea", "[25] Indonesien", "[38] Philippinen", "[40] Japan",

"[42] Indien", "[44] Thailand", "[50] Bangla Desh", "[63] Hongkong",

"[65] Sri Lanka", "[66] Nepal", "[68] China", "[83] Vietnam",

"[93] Singapur", "[100] Laos", "[104] Malaysia", "[128] Malaysia",

"[145] Mongolei", "[154] Taiwan", "[160] Osttimor",

"[163] Malediven", "[169] Kambodscha", "[181] Myanmar")

SWAsia <- c("[74] Kasachstan", "[73] Moldawien", "[91] Turkmenistan-UdSSR (bis 1991)",

"[77] Kirgistan", "[82] Tadschikistan", "[97] Usbekistan",

"[33] Kurdistan", "[130] Aserbaidschan",

"[141] Georgien", "[148] Armenien",

"[149] Kurdistan", "[155] Turkmenistan", "[172] Kaukasus")

centralasia <- c("[74] Kasachstan", "[91] Turkmenistan-UdSSR (bis 1991)",

"[77] Kirgistan", "[82] Tadschikistan", "[97] Usbekistan",

"[33] Kurdistan",

"[149] Kurdistan", "[155] Turkmenistan", "[43] Afghanistan")

mideast <- c("[76] Libanon", "[24] Iran", "[30] Syrien", "[39] Israel", "[60] Irak",

"[46] Saudi-Arabien", "[87] Vereinigte Arabische Emirate", "[90] Jordanien",

"[126] Kuwait", "[136] Oman", "[151] Jemen", "[152] Palaestina",

"[161] Bahrain", "[193] Katar", "[81] Aegypten", "[2] Tuerkei")

GMEast <- c("[76] Libanon", "[24] Iran", "[30] Syrien", "[39] Israel", "[60] Irak",

"[46] Saudi-Arabien","[87] Vereinigte Arabische Emirate", "[90] Jordanien",

"[126] Kuwait", "[136] Oman", "[151] Jemen", "[152] Palaestina",

"[161] Bahrain", "[193] Katar", "[81] Aegypten", "[2] Tuerkei", "[58] Zypern",

"[43] Afghanistan","[85] Pakistan",

"[52] Tunesien",

"[67] Marokko", "[79] Algerien",

"[111] Libyen", "[142] Sudan", "[190] Dschibuti", "[84] Somalia")

Australia <- c("[41] Australien", "[56] Neuseeland", "[137] Mikronesien",

"[182] Fidschi", "[129] Samoa")

Americas <- c("[18] USA", "[55] Kanada", "[164] Hawaii")

CSAmerica <- c("[20] Chile", "[27] Bolivien", "[34] Mexiko", "[35] Argentinien ",

"[45] Jamaika", "[48] Kolumbien", "[51] Venezuela", "[59] Kuba",

"[64] Peru", "[72] St. Lucia", "[88] El Salvador", "[92] Costa Rica",

"[96] Ecuador", "[99] Puerto Rico", "[107] Belize",

"[108] Dominikanische Republik", "[109] Nicaragua", "[114] Haiti",

"[124] Paraguay", "[133] Uruguay", "[134] Bahamas", "[157] Guatemala",

"[159] Panama", "[167] Honduras", "[170] Surinam", "[171] Guyana",

"[175] Grenada", "[61] Brasilien")

NorthAfrica <- c("[47] Aethiopien",

"[52] Tunesien",

"[67] Marokko", "[79] Algerien",

"[81] Aegypten", "[89] Eritrea",

"[111] Libyen",

"[138] Mali", "[142] Sudan",

"[183] Niger", "[190] Dschibuti")

MSAfrica <- c("[36] Cap Verden", "[37] Benin", "[49] Ghana", "[53] Mauritius",

"[54] Nigeria", "[57] Tansania", "[80] Mocambique",

"[84] Somalia", "[86] Suedafrika",

"[94] Burkina Faso", "[95] Sambia", "[102] Angola", "[105] Namibia",

"[110] Kenia", "[113] Botswana", "[115] Trinidad, Tobago",

"[125] Guinea", "[127] Elfenbeinkueste", "[131] Seychellen",

"[135] Uganda", "[139] Kamerun", "[143] Kongo", "[144] Togo",

"[147] Tschad", "[150] Liberia", "[156] Afrika", "[158] Sierra Leone(Westafrika)",

"[162] Senegal", "[166] Gambia", "[173] Simbabwe", "[174] Madagaskar",

"[176] Lesotho", "[177] Bhutan", "[178] Ruanda", "[179] Malawi")

Africa <- c("[47] Aethiopien",

"[52] Tunesien",

"[67] Marokko", "[79] Algerien",

"[81] Aegypten", "[89] Eritrea",

"[111] Libyen",

"[138] Mali", "[142] Sudan",

"[183] Niger", "[190] Dschibuti",

"[36] Cap Verden", "[37] Benin", "[49] Ghana", "[53] Mauritius",

"[54] Nigeria", "[57] Tansania", "[80] Mocambique",

"[84] Somalia", "[86] Suedafrika",

"[94] Burkina Faso", "[95] Sambia", "[102] Angola", "[105] Namibia",

"[110] Kenia", "[113] Botswana", "[115] Trinidad, Tobago",

"[125] Guinea", "[127] Elfenbeinkueste", "[131] Seychellen",

"[135] Uganda", "[139] Kamerun", "[143] Kongo", "[144] Togo",

"[147] Tschad", "[150] Liberia", "[156] Afrika", "[158] Sierra Leone(Westafrika)",

"[162] Senegal", "[166] Gambia", "[173] Simbabwe", "[174] Madagaskar",

"[176] Lesotho", "[177] Bhutan", "[178] Ruanda", "[179] Malawi")

dfs <- mutate(dfs, region = ifelse(corigin %in% Germany, 1,

ifelse(corigin %in% easteurope, 2,

ifelse(corigin %in% westeurope, 3,

ifelse(corigin %in% Asia, 4,

ifelse(corigin %in% centralasia, 5,

ifelse(corigin %in% mideast, 6,

ifelse(corigin %in% Americas, 7,

ifelse(corigin %in% CSAmerica, 8,

ifelse(corigin %in% Australia, 9,

ifelse(corigin %in% NorthAfrica, 10,

ifelse(corigin %in% MSAfrica, 11, NA))))))))))))

table(dfs$region, exclude = NULL)

#Asia 85, Americas 22, CSAmericas 47, Australia & Oceana 5

dfs <- mutate(dfs, regionc = ifelse(corigin %in% Germany, 1,

ifelse(corigin %in% easteurope, 2,

ifelse(corigin %in% westeurope, 3,

ifelse(corigin %in% centralasia, 4,

ifelse(corigin %in% mideast, 5, NA))))))

table(dfs$regionc, exclude = NULL)

dfs$regionc <- dplyr::recode_factor(dfs$regionc, '1' = "Germany",

'2' = "Eastern Europe",

'3' = "Western Europe",

'4' = "Central Asia",

'5' = "Middle East",

as.default = NA_character_)

table(dfs$regionc, exclude = NULL)

#by continent

Europe <- c("[2] Tuerkei", "[3] Ex-Jugoslawien",

"[21] Rumaenien", "[22] Polen", "[26] Ungarn", "[29] Bulgarien",

"[31] Tschechien", "[32] Russland", "[75] Albanien",

"[78] Ukraine", "[140] Kosovo-Albaner",

"[165] Serbien", "[101] Estland",

"[103] Lettland", "[106] Montenegro", "[119] Kroatien",

"[120] Bosnien/Herzegowina", "[121] Makedonien", "[122] Slowenien",

"[123] Slowakei", "[132] Weissrussland", "[146] Litauen",

"[153] Freistaat Danzig", "[165] Serbien", "[168] Montenegro",

"[180] Bessarabien", "[222] Eastern Europe",

"[4] Griechenland","[5] Italien", "[6] Spanien",

"[10] Oesterreich", "[11] Frankreich", "[12] Benelux", "[13] Daenemark",

"[14] Grossbritannien", "[15] Schweden", "[16] Norwegen", "[17] Finnland",

"[19] Schweiz", "[28] Portugal",

"[58] Zypern", "[112] Malta",

"[62] Monaco", "[69] Liechtenstein", "[70] Island", "[71] Irland",

"[116] Luxemburg", "[117] Belgien", "[118] Niederlande")

Asia <- c("[23] Korea", "[25] Indonesien", "[38] Philippinen", "[40] Japan",

"[42] Indien", "[44] Thailand", "[50] Bangla Desh", "[63] Hongkong",

"[65] Sri Lanka", "[66] Nepal", "[68] China", "[83] Vietnam",

"[93] Singapur", "[100] Laos", "[104] Malaysia", "[128] Malaysia",

"[145] Mongolei", "[154] Taiwan", "[160] Osttimor",

"[163] Malediven", "[169] Kambodscha", "[181] Myanmar",

"[74] Kasachstan", "[73] Moldawien", "[91] Turkmenistan-UdSSR (bis 1991)",

"[77] Kirgistan", "[82] Tadschikistan", "[97] Usbekistan", "[85] Pakistan",

"[33] Kurdistan", "[43] Afghanistan", "[130] Aserbaidschan",

"[141] Georgien", "[148] Armenien",

"[149] Kurdistan", "[155] Turkmenistan", "[172] Kaukasus",

"[76] Libanon", "[24] Iran", "[30] Syrien", "[39] Israel", "[60] Irak",

"[46] Saudi-Arabien","[87] Vereinigte Arabische Emirate", "[90] Jordanien",

"[126] Kuwait", "[136] Oman", "[151] Jemen", "[152] Palaestina",

"[161] Bahrain", "[193] Katar")

Australia <- c("[41] Australien", "[56] Neuseeland", "[137] Mikronesien",

"[182] Fidschi", "[129] Samoa")

NAmerica <- c("[18] USA", "[55] Kanada", "[164] Hawaii")

CSAmerica <- c("[20] Chile", "[27] Bolivien", "[34] Mexiko", "[35] Argentinien ",

"[45] Jamaika", "[48] Kolumbien", "[51] Venezuela", "[59] Kuba",

"[64] Peru", "[72] St. Lucia", "[88] El Salvador", "[92] Costa Rica",

"[96] Ecuador", "[99] Puerto Rico", "[107] Belize",

"[108] Dominikanische Republik", "[109] Nicaragua", "[114] Haiti",

"[124] Paraguay", "[133] Uruguay", "[134] Bahamas", "[157] Guatemala",

"[159] Panama", "[167] Honduras", "[170] Surinam", "[171] Guyana",

"[175] Grenada", "[61] Brasilien")

Africa <- c("[47] Aethiopien",

"[52] Tunesien",

"[67] Marokko", "[79] Algerien",

"[81] Aegypten", "[89] Eritrea",

"[111] Libyen",

"[138] Mali", "[142] Sudan",

"[183] Niger", "[190] Dschibuti",

"[36] Cap Verden", "[37] Benin", "[49] Ghana", "[53] Mauritius",

"[54] Nigeria", "[57] Tansania", "[80] Mocambique",

"[84] Somalia", "[86] Suedafrika",

"[94] Burkina Faso", "[95] Sambia", "[102] Angola", "[105] Namibia",

"[110] Kenia", "[113] Botswana", "[115] Trinidad, Tobago",

"[125] Guinea", "[127] Elfenbeinkueste", "[131] Seychellen",

"[135] Uganda", "[139] Kamerun", "[143] Kongo", "[144] Togo",

"[147] Tschad", "[150] Liberia", "[156] Afrika", "[158] Sierra Leone(Westafrika)",

"[162] Senegal", "[166] Gambia", "[173] Simbabwe", "[174] Madagaskar",

"[176] Lesotho", "[177] Bhutan", "[178] Ruanda", "[179] Malawi")

#NSAmerica 26, CSmerica 64, Australia 6

dfs <- mutate(dfs, continent = ifelse(corigin %in% Europe, 1,

ifelse(corigin %in% Asia, 2,

ifelse(corigin %in% Africa, 3,

ifelse(corigin %in% Germany, 4, NA)))))

table(dfs$continent, exclude = NULL)

dfs$continentc <- dplyr::recode_factor(dfs$continent, '1' = "Europe",

'2' = "Asia",

'3' = "Africa",

'4' = "Germany",

as.default = NA_character_)

table(dfs$continentc, exclude = NULL)

#BORN IN GERMANY

table(dfs$germborn, exclude = NULL)

dfs$germbornr <- as.character(dfs$germborn)

dfs$germbornr <- dplyr::recode_factor(dfs$germbornr, '[1] in Deutschland geboren oder immigr.<1950' = "born in Germany or immigr.<1950",

'[2] nicht in Deutschland geboren' = "not born in Germany",

as.default = NA_character_)

table(dfs$germbornr, exclude = NULL)

#RESIDENCE STATUS

table(dfs$biresper, exclude = NULL)

dfs$resistat <- as.character(dfs$biresper)

dfs$resistatr <- dplyr::recode_factor(dfs$resistat, '[1] Unbefristet' = "Unlimited", '[2] Befristet' = "Temporary",

as.default = NA_character_)

table(dfs$resistatr, exclude = NULL)

class(dfs$resistatr)

#LENGTH OF STAY IN GERMANY IN YEARS immiyear

mean(dfs$immiyear, na.rm=TRUE)

median(dfs$immiyear, na.rm=TRUE)

sd(dfs$immiyear, na.rm=TRUE)

max(dfs$immiyear, na.rm=TRUE)

min(dfs$immiyear, na.rm=TRUE)

descr(dfs$immiyear)

p <- dfs %>% ggplot(aes(immiyear)) + geom_histogram(binwidth = 1)

p

sort(table(dfs$immiyear),decreasing=T, exclude = NULL)

#length of stay in categories

#dichotomous - <=5 years or more

#1 immigrated between 2014-2018, 0 before

dfs <- mutate(dfs, imyear5 = ifelse(immiyear %in% 2014:2018, 1,

ifelse(immiyear %in% 1950:2013, 0, NA)))

table(dfs$imyear5, exclude = NULL)

dfs$imyear5 <- dplyr::recode_factor(dfs$imyear5, '0' = "before 2014", '1' = "2014 to 2018")

frq(dfs$imyear5)

#5year, 10 years, 15 years, 20 years, longer

dfs <- mutate(dfs, imyearcat = ifelse(immiyear %in% 2014:2018, 0,

ifelse(immiyear %in% 2009:2013, 1,

ifelse(immiyear %in% 2004:2008, 2,

ifelse(immiyear %in% 1999:2003, 3,

ifelse(immiyear %in% 1950:1998, 4, NA))))))

table(dfs$imyearcat, exclude = NULL)

dfs$imyearcatr <- as.character(dfs$imyearcat)

dfs$imyearcatr <- dplyr::recode_factor(dfs$imyearcatr, '0' = "2014 to 2018",

'1' = "2009 to 2013",

'2' = "2004 to 2008",

'3' = "1999 to 2003",

'4' = "before 1999", .default = NA_character_)

table(dfs$imyearcatr, exclude = NULL)

#IMMIGRANT GROUP

table(dfs$biimgrp, exclude = NULL)

dfs$imgrpr <- as.character(dfs$biimgrp)

dfs$imgrpr <- dplyr::recode_factor(dfs$imgrpr, '[2] Aussiedler, Osteuropa' = "Person of German descent from Eastern Europe",

'[3] Deutsche, Ausland' = "German who lived abroad",

'[4] EU-Mitgliedsstaat (bis 2009 EG)' = "Citizen of EU country (up to 2009 EC)",

'[5] Asylbewerber,Fluechtling' = "Asylum seeker, refugee",

'[6] Sonstige Auslaender' = "Other foreigner", .default = NA_character_)

table(dfs$imgrpr, exclude = NULL)

#COMPOSITE GENDER MEASURE

#bip_197_01 A person who is living with their partner for the long term should get married

#1 full disagreement to 7 full agreement

table(dfs$bip_197_01, exclude = NULL)

dfs$bip_197_01 <- dplyr::recode(dfs$bip_197_01, '[1] Stimme ueberhaupt nicht zu' = 1,

'[2] 2' = 2,

'[3] 3' = 3,

'[4] 4' = 4,

'[5] 5' = 5,

'[6] 6' = 6,

'[7] Stimme voll zu' = 7)

table(dfs$bip_197_01)

class(dfs$bip_197_01)

# bip_197_02 I think it is good that marriages between two women or two men are legally recognized

#1 full disagreement to 7 full agreement

table(dfs$bip_197_02, exclude = NULL)

dfs$bip_197_02 <- dplyr::recode(dfs$bip_197_02, '[1] Stimme ueberhaupt nicht zu' = 1,

'[2] 2' = 2,

'[3] 3' = 3,

'[4] 4' = 4,

'[5] 5' = 5,

'[6] 6' = 6,

'[7] Stimme voll zu' = 7)

table(dfs$bip_197_02)

#bip_197_03 A single parent can raise a child just as well as two parents together

#this can have two meanings: either based on traditional family ideals or because single parents aren't supported enough in our society

#1 full disagreement to 7 full agreement

table(dfs$bip_197_03, exclude = NULL)

dfs$bip_197_03 <- dplyr::recode(dfs$bip_197_03, '[1] Stimme ueberhaupt nicht zu' = 1,

'[2] 2' = 2,

'[3] 3' = 3,

'[4] 4' = 4,

'[5] 5' = 5,

'[6] 6' = 6,

'[7] Stimme voll zu' = 7)

table(dfs$bip_197_03)

#bip_197_04 Children below the age of 6 suffer if their mother works

#1 full disagreement to 7 full agreement

table(dfs$bip_197_04, exclude = NULL)

dfs$bip_197_04 <- dplyr::recode(dfs$bip_197_04, '[1] Stimme ueberhaupt nicht zu' = 1,

'[2] 2' = 2,

'[3] 3' = 3,

'[4] 4' = 4,

'[5] 5' = 5,

'[6] 6' = 6,

'[7] Stimme voll zu' = 7)

table(dfs$bip_197_04)

#bip_197_05 Children below the age of 3 suffer if their mother works

#1 full disagreement to 7 full agreement

table(dfs$bip_197_05, exclude = NULL)

dfs$bip_197_05 <- dplyr::recode(dfs$bip_197_05, '[1] Stimme ueberhaupt nicht zu' = 1,

'[2] 2' = 2,

'[3] 3' = 3,

'[4] 4' = 4,

'[5] 5' = 5,

'[6] 6' = 6,

'[7] Stimme voll zu' = 7)

table(dfs$bip_197_05)

#bip_197_06 Best if man and woman work the same amount so they can share the responsibility

#1 full disagreement to 7 full agreement

table(dfs$bip_197_06, exclude = NULL)

dfs$bip_197_06 <- dplyr::recode(dfs$bip_197_06, '[1] Stimme ueberhaupt nicht zu' = 1,

'[2] 2' = 2,

'[3] 3' = 3,

'[4] 4' = 4,

'[5] 5' = 5,

'[6] 6' = 6,

'[7] Stimme voll zu' = 7)

table(dfs$bip_197_06)

#bip_197_07 A same-sex couple can raise a child just as well as a man and woman

#1 full disagreement to 7 full agreement

table(dfs$bip_197_07, exclude = NULL)

dfs$bip_197_07 <- dplyr::recode(dfs$bip_197_07, '[1] Stimme ueberhaupt nicht zu' = 1,

'[2] 2' = 2,

'[3] 3' = 3,

'[4] 4' = 4,

'[5] 5' = 5,

'[6] 6' = 6,

'[7] Stimme voll zu' = 7)

table(dfs$bip_197_07)

#bip_197_08 It would be good for society if transgender people were recognized as normal

#1 full disagreement to 7 full agreement

table(dfs$bip_197_08, exclude = NULL)

dfs$bip_197_08 <- dplyr::recode(dfs$bip_197_08, '[1] Stimme ueberhaupt nicht zu' = 1,

'[2] 2' = 2,

'[3] 3' = 3,

'[4] 4' = 4,

'[5] 5' = 5,

'[6] 6' = 6,

'[7] Stimme voll zu' = 7)

table(dfs$bip_197_08)

#Economic and power relations

#pgexppt Working Experience Part-Time Employment - numeric

#This variable reflects the total length of part-time employment in the respondent’s career up to the point of the interview in a given year. The variable is created by combining monthly information on employment status from the calendar dataset ARTKALEN (which provides monthly information on activity status since an individual entered the SOEP) and annual information from the biographical dataset PBIOSPE (which provides information on activity status over the life course of an individual). PGEXPPT gives the length of time in years with months in decimal form.

class(dfs$pgexppt)

dfs$pgexppt <- as.numeric(dfs$pgexppt)

mean(dfs$pgexppt, na.rm=TRUE)

median(dfs$pgexppt, na.rm=TRUE)

sd(dfs$pgexppt, na.rm=TRUE)

p <- dfs %>% ggplot(aes(pgexppt)) + geom_histogram(binwidth = 1)

p

#pgexpft Working Experience Full-Time Employment - numeric

#total length of full-time employment in the respondent’s career up to the point of the interview in a given year. The variable is created by combining monthly information on employment status from the calendar dataset ARTKALEN (which provides monthly information on activity status since an individual entered the SOEP) and annual information from the biographical dataset PBIOSPE (which provides information on activity status over the individual’s life course). PGEXPFT gives the length of time in years with months in decimal form.

class(dfs$pgexpft)

dfs$pgexpft <- as.numeric(dfs$pgexpft)

mean(dfs$pgexpft, na.rm=TRUE)

median(dfs$pgexpft, na.rm=TRUE)

sd(dfs$pgexpft, na.rm=TRUE)

p <- dfs %>% ggplot(aes(pgexpft)) + geom_histogram(binwidth = 1)

p

#pli0043_h Hours Weekdays Housework

class(dfs$pli0043_h)

dfs$pli0043_h <- as.numeric(dfs$pli0043_h)

mean(dfs$pli0043_h, na.rm=TRUE)

median(dfs$pli0043_h, na.rm=TRUE)

sd(dfs$pli0043_h, na.rm=TRUE)

min(dfs$pli0043_h, na.rm=TRUE)

max(dfs$pli0043_h, na.rm=TRUE)

p <- dfs %>% ggplot(aes(pli0043_h)) + geom_histogram(binwidth = 1)

p

#pli0046 Hours Weekdays Care For Persons

class(dfs$pli0046)

dfs$pli0046 <- as.numeric(dfs$pli0046)

#pli0051 Hours Weekdays Leisure, Hobbies

class(dfs$pli0051)

dfs$pli0051 <- as.numeric(dfs$pli0051)

#pli0162 Koerperl. Aktivitaeten Std., Werktg.

dfs$pli0162 <- as.numeric(dfs$pli0162)

#pli0049_h Reparaturen etc. Std., Werktag [harmonisiert]

dfs$pli0049_h <- as.numeric(dfs$pli0049_h)

#Affective relations

#plh0174 Satisfaction With Housework

#0 low satisfaction t0 10 high satisfaction

table(dfs$plh0174, exclude = NULL)

dfs$plh0174 <- dplyr::recode(dfs$plh0174, '[0] 0 Zufrieden: Skala 0-Niedrig bis 10-Hoch' = 0,

'[1] 1 Zufrieden: Skala 0-Niedrig bis 10-Hoch' = 1,

'[2] 2 Zufrieden: Skala 0-Niedrig bis 10-Hoch' = 2,

'[3] 3 Zufrieden: Skala 0-Niedrig bis 10-Hoch' = 3,

'[4] 4 Zufrieden: Skala 0-Niedrig bis 10-Hoch' = 4,

'[5] 5 Zufrieden: Skala 0-Niedrig bis 10-Hoch' = 5,

'[6] 6 Zufrieden: Skala 0-Niedrig bis 10-Hoch' = 6,

'[7] 7 Zufrieden: Skala 0-Niedrig bis 10-Hoch' = 7,

'[8] 8 Zufrieden: Skala 0-Niedrig bis 10-Hoch' = 8,

'[9] 9 Zufrieden: Skala 0-Niedrig bis 10-Hoch' = 9,

'[10] 10 Zufrieden: Skala 0-Niedrig bis 10-Hoch' = 10)

table(dfs$plh0174)

#plh0176 Satisfaction With Personal Income

#0 low satisfaction t0 10 high satisfaction

table(dfs$plh0176, exclude = NULL)

dfs$plh0176 <- dplyr::recode(dfs$plh0176, '[0] 0 Zufrieden: Skala 0-Niedrig bis 10-Hoch' = 0,

'[1] 1 Zufrieden: Skala 0-Niedrig bis 10-Hoch' = 1,

'[2] 2 Zufrieden: Skala 0-Niedrig bis 10-Hoch' = 2,

'[3] 3 Zufrieden: Skala 0-Niedrig bis 10-Hoch' = 3,

'[4] 4 Zufrieden: Skala 0-Niedrig bis 10-Hoch' = 4,

'[5] 5 Zufrieden: Skala 0-Niedrig bis 10-Hoch' = 5,

'[6] 6 Zufrieden: Skala 0-Niedrig bis 10-Hoch' = 6,

'[7] 7 Zufrieden: Skala 0-Niedrig bis 10-Hoch' = 7,

'[8] 8 Zufrieden: Skala 0-Niedrig bis 10-Hoch' = 8,

'[9] 9 Zufrieden: Skala 0-Niedrig bis 10-Hoch' = 9,

'[10] 10 Zufrieden: Skala 0-Niedrig bis 10-Hoch' = 10)

table(dfs$plh0176)

#plh0178 Satisfaction With Amount Of Leisure Time

#0 low satisfaction t0 10 high satisfaction

table(dfs$plh0178, exclude = NULL)

dfs$plh0178 <- dplyr::recode(dfs$plh0178, '[0] 0 Zufrieden: Skala 0-Niedrig bis 10-Hoch' = 0,

'[1] 1 Zufrieden: Skala 0-Niedrig bis 10-Hoch' = 1,

'[2] 2 Zufrieden: Skala 0-Niedrig bis 10-Hoch' = 2,

'[3] 3 Zufrieden: Skala 0-Niedrig bis 10-Hoch' = 3,

'[4] 4 Zufrieden: Skala 0-Niedrig bis 10-Hoch' = 4,

'[5] 5 Zufrieden: Skala 0-Niedrig bis 10-Hoch' = 5,

'[6] 6 Zufrieden: Skala 0-Niedrig bis 10-Hoch' = 6,

'[7] 7 Zufrieden: Skala 0-Niedrig bis 10-Hoch' = 7,

'[8] 8 Zufrieden: Skala 0-Niedrig bis 10-Hoch' = 8,

'[9] 9 Zufrieden: Skala 0-Niedrig bis 10-Hoch' = 9,

'[10] 10 Zufrieden: Skala 0-Niedrig bis 10-Hoch' = 10)

table(dfs$plh0178)

#plh0162 Zufriedenheit Lebensstandard

table(dfs$plh0162, exclude = NULL)

dfs$plh0162 <- dplyr::recode(dfs$plh0162, '[0] 0 Zufrieden: Skala 0-Niedrig bis 10-Hoch' = 0,

'[1] 1 Zufrieden: Skala 0-Niedrig bis 10-Hoch' = 1,

'[2] 2 Zufrieden: Skala 0-Niedrig bis 10-Hoch' = 2,

'[3] 3 Zufrieden: Skala 0-Niedrig bis 10-Hoch' = 3,

'[4] 4 Zufrieden: Skala 0-Niedrig bis 10-Hoch' = 4,

'[5] 5 Zufrieden: Skala 0-Niedrig bis 10-Hoch' = 5,

'[6] 6 Zufrieden: Skala 0-Niedrig bis 10-Hoch' = 6,

'[7] 7 Zufrieden: Skala 0-Niedrig bis 10-Hoch' = 7,

'[8] 8 Zufrieden: Skala 0-Niedrig bis 10-Hoch' = 8,

'[9] 9 Zufrieden: Skala 0-Niedrig bis 10-Hoch' = 9,

'[10] 10 Zufrieden: Skala 0-Niedrig bis 10-Hoch' = 10)

table(dfs$plh0162)

#plh0172 Zufriedenheit Schlaf

table(dfs$plh0172, exclude = NULL)

dfs$plh0172 <- dplyr::recode(dfs$plh0172, '[0] 0 Zufrieden: Skala 0-Niedrig bis 10-Hoch' = 0,

'[1] 1 Zufrieden: Skala 0-Niedrig bis 10-Hoch' = 1,

'[2] 2 Zufrieden: Skala 0-Niedrig bis 10-Hoch' = 2,

'[3] 3 Zufrieden: Skala 0-Niedrig bis 10-Hoch' = 3,

'[4] 4 Zufrieden: Skala 0-Niedrig bis 10-Hoch' = 4,

'[5] 5 Zufrieden: Skala 0-Niedrig bis 10-Hoch' = 5,

'[6] 6 Zufrieden: Skala 0-Niedrig bis 10-Hoch' = 6,

'[7] 7 Zufrieden: Skala 0-Niedrig bis 10-Hoch' = 7,

'[8] 8 Zufrieden: Skala 0-Niedrig bis 10-Hoch' = 8,

'[9] 9 Zufrieden: Skala 0-Niedrig bis 10-Hoch' = 9,

'[10] 10 Zufrieden: Skala 0-Niedrig bis 10-Hoch' = 10)

table(dfs$plh0172)

#plh0180 Zufriedenheit Family life

table(dfs$plh0180, exclude = NULL)

dfs$plh0180 <- dplyr::recode(dfs$plh0180, '[0] 0 Zufrieden: Skala 0-Niedrig bis 10-Hoch' = 0,

'[1] 1 Zufrieden: Skala 0-Niedrig bis 10-Hoch' = 1,

'[2] 2 Zufrieden: Skala 0-Niedrig bis 10-Hoch' = 2,

'[3] 3 Zufrieden: Skala 0-Niedrig bis 10-Hoch' = 3,

'[4] 4 Zufrieden: Skala 0-Niedrig bis 10-Hoch' = 4,

'[5] 5 Zufrieden: Skala 0-Niedrig bis 10-Hoch' = 5,

'[6] 6 Zufrieden: Skala 0-Niedrig bis 10-Hoch' = 6,

'[7] 7 Zufrieden: Skala 0-Niedrig bis 10-Hoch' = 7,

'[8] 8 Zufrieden: Skala 0-Niedrig bis 10-Hoch' = 8,

'[9] 9 Zufrieden: Skala 0-Niedrig bis 10-Hoch' = 9,

'[10] 10 Zufrieden: Skala 0-Niedrig bis 10-Hoch' = 10)

table(dfs$plh0180)

#plh0033 Worried About Finances

table(dfs$plh0033, exclude = NULL)

dfs$plh0033 <- dplyr::recode(dfs$plh0033, '[1] Grosse Sorgen' = 1,

'[2] Einige Sorgen' = 2,

'[3] Keine Sorgen' = 3)

table(dfs$plh0033, exclude = NULL)

#plh0335 Worried About Own Retirement Pension

table(dfs$plh0335, exclude = NULL)

dfs$plh0335 <- dplyr::recode(dfs$plh0335, '[1] Grosse Sorgen' = 1,

'[2] Einige Sorgen' = 2,

'[3] Keine Sorgen' = 3)

table(dfs$plh0335, exclude = NULL)

#plh0036 Sorgen Umweltschutz

table(dfs$plh0036, exclude = NULL)

dfs$plh0036 <- dplyr::recode(dfs$plh0036, '[1] Grosse Sorgen' = 1,

'[2] Einige Sorgen' = 2,

'[3] Keine Sorgen' = 3)

table(dfs$plh0036, exclude = NULL)

#plh0037 Sorgen Klimawandelfolgen

table(dfs$plh0037, exclude = NULL)

dfs$plh0037 <- dplyr::recode(dfs$plh0037, '[1] Grosse Sorgen' = 1,

'[2] Einige Sorgen' = 2,

'[3] Keine Sorgen' = 3)

table(dfs$plh0037, exclude = NULL)

#plh0038 Sorgen Friedenserhaltung

table(dfs$plh0038, exclude = NULL)

dfs$plh0038 <- dplyr::recode(dfs$plh0038, '[1] Grosse Sorgen' = 1,

'[2] Einige Sorgen' = 2,

'[3] Keine Sorgen' = 3)

table(dfs$plh0038, exclude = NULL)

#plh0039 Sorgen globalen Terrorismus

table(dfs$plh0039, exclude = NULL)

dfs$plh0039 <- dplyr::recode(dfs$plh0039, '[1] Grosse Sorgen' = 1,

'[2] Einige Sorgen' = 2,

'[3] Keine Sorgen' = 3)

table(dfs$plh0039, exclude = NULL)

#plh0040 Sorgen globalen Terrorismus

table(dfs$plh0040, exclude = NULL)

dfs$plh0040 <- dplyr::recode(dfs$plh0040, '[1] Grosse Sorgen' = 1,

'[2] Einige Sorgen' = 2,

'[3] Keine Sorgen' = 3)

table(dfs$plh0040, exclude = NULL)

#plb0018 Bezahlte Arbeit letzte 7 Tage dicho

table(dfs$plb0018)

dfs$plb0018 <- dplyr::recode(dfs$plb0018, '[1] Ja' = 1,

'[2] Nein' = 2)

table(dfs$plb0018, exclude = NULL)

#plh0184 Haeufigkeit aergerlich letzte 4 Wochen

table(dfs$plh0184)

dfs$plh0184 <- dplyr::recode(dfs$plh0184, '[1] Sehr selten' = 1,

'[2] Selten' = 2,

'[3] Manchmal' = 3,

'[4] Oft' = 4,

'[5] Sehr oft' = 5)

table(dfs$plh0184, exclude = NULL)

#plh0185 Haeufigkeit aengstlich letzte 4 Wochen

table(dfs$plh0185)

dfs$plh0185 <- dplyr::recode(dfs$plh0185, '[1] Sehr selten' = 1,

'[2] Selten' = 2,

'[3] Manchmal' = 3,

'[4] Oft' = 4,

'[5] Sehr oft' = 5)

table(dfs$plh0185, exclude = NULL)

#plh0186 Haeufigkeit gluecklich letzte 4 Wochen

table(dfs$plh0186)

dfs$plh0186 <- dplyr::recode(dfs$plh0186, '[1] Sehr selten' = 1,

'[2] Selten' = 2,

'[3] Manchmal' = 3,

'[4] Oft' = 4,

'[5] Sehr oft' = 5)

table(dfs$plh0186, exclude = NULL)

#plh0187 Haeufigkeit traurig letzte 4 Wochen

table(dfs$plh0187)

dfs$plh0187 <- dplyr::recode(dfs$plh0187, '[1] Sehr selten' = 1,

'[2] Selten' = 2,

'[3] Manchmal' = 3,

'[4] Oft' = 4,

'[5] Sehr oft' = 5)

table(dfs$plh0187, exclude = NULL)

#plh0189 Fuehle micht oft einsam

table(dfs$plh0189)

dfs$plh0189 <- dplyr::recode(dfs$plh0189, '[1] Stimmt ganz u.gar' = 1,

'[2] Stimmt eher' = 2,

'[3] Stimmt eher nicht' = 3,

'[4] Stimmt ganz u.gar nicht' = 4)

table(dfs$plh0189, exclude = NULL)

#plh0193 Heute kein Verlass auf niemand

table(dfs$plh0193)

dfs$plh0193 <- dplyr::recode(dfs$plh0193, '[1] Stimme voll zu' = 1,

'[2] Stimme eher zu' = 2,

'[3] Lehne eher ab' = 3,

'[4] Lehne voll ab' = 4)

table(dfs$plh0193, exclude = NULL)

#plh0195 Leute nutzen aus, sind fair

table(dfs$plh0195)

dfs$plh0195 <- dplyr::recode(dfs$plh0195, '[1] Leute nutzen aus' = 1,

'[2] Leute sind fair' = 2)

table(dfs$plh0195, exclude = NULL)

#plh0204_h Selbsteinschaetzung Risikobereitschaft [harmonisiert]

table(dfs$plh0204_h)

dfs$plh0204_h <- dplyr::recode(dfs$plh0204_h, '[0] 0 Risikobereit Skala 0-Gar nicht, 10-Sehr' = 0,

'[1] 1 Risikobereit Skala 0-Gar nicht, 10-Sehr' = 1,

'[2] 2 Risikobereit Skala 0-Gar nicht, 10-Sehr' = 2,

'[3] 3 Risikobereit Skala 0-Gar nicht, 10-Sehr' = 3,

'[4] 4 Risikobereit Skala 0-Gar nicht, 10-Sehr' = 4,

'[5] 5 Risikobereit Skala 0-Gar nicht, 10-Sehr' = 5,

'[6] 6 Risikobereit Skala 0-Gar nicht, 10-Sehr' = 6,

'[7] 7 Risikobereit Skala 0-Gar nicht, 10-Sehr' = 7,

'[8] 8 Risikobereit Skala 0-Gar nicht, 10-Sehr' = 8,

'[9] 9 Risikobereit Skala 0-Gar nicht, 10-Sehr' = 9,

'[10] 10 Risikobereit Skala 0-Gar nicht, 10-Sehr' = 10)

table(dfs$plh0204_h)

glimpse(dfs)

dfs_recoded <- dfs

dfs$cid

save(dfs_recoded, file = "dfs_recoded.Rda")

#---------------------------------------------------------------#

#SAMPLE SELECTION FOR PRINCIPAL COMPONENT ANALYSIS

#individuals aged 18 years and older that participated in wave 35 (2018), have completed the health questions and gender-related variables

#wave 2018

# n= 30306

nrow(subset(dfs, syear=="2018"))

#18 years and older

df_sample2018 <- subset(dfs, d11101 >= 18)

#n= 30220, excludes 86

30306-30220

#answered the gender-related questions

#gender-related variables - check for missings

library(sjmisc)

keep <- c("bip_197_01", "bip_197_02", "bip_197_03", "bip_197_04",

"bip_197_05", "bip_197_06", "bip_197_07", "bip_197_08",

"pgexpft", "pgexppt",

"plb0018", "plb0037_h", "plb0241_h", "plc0010", "pld0062_v2",

"pld0068_v2", "pld0083_v2", "plh0033", "plh0036",

"plh0037", "plh0038", "plh0039", "plh0040",

"plh0162", "plh0171", "plh0172", "plh0174", "plh0176", "plh0178",

"plh0184", "plh0185", "plh0186", "plh0187", "plh0189", "plh0193",

"plh0195", "plh0204_h", "plh0335", "pli0043_h", "pli0046",

"pli0049_h", "pli0051", "pli0162", "plj0566", "plj0567")

df1g <- df1[keep]

glimpse(df1g)

sum_allgendv <- descr(df1g)

write.csv(sum_allgendv, "sum_allgendv.csv")

0.25*29456

#exclude variables due to missings (more than 25% missing):

#plj0567

#plj0566

#pld0083_v2

#pld0062_v2

#pld0068_v2

#plb0241_h

#plc0010

#plb0037_h

#exclude content-wise variables

#too closely related with health behaviours/mental health indicators

#plh0185

#plh0186

#plh0187

#plh0184

#plh0189

#plh0172

#pli0162

dfs$plj0567 <- NULL

dfs$plj0566 <- NULL

dfs$pld0083_v2 <- NULL

dfs$pld0062_v2 <- NULL

dfs$pld0068_v2 <- NULL

dfs$plb0241_h <- NULL

dfs$plc0010 <- NULL

dfs$plb0037_h <- NULL

#keep all other variables

df_pca <- df_sample2018[complete.cases(df1$bip_197_01,

df1$bip_197_02,

df1$bip_197_03,

df1$bip_197_04,

df1$bip_197_05,

df1$bip_197_06,

df1$bip_197_07,

df1$bip_197_08,

df1$pgexpft,

df1$pgexppt,

df1$plb0018,

df1$plh0033,

df1$plh0036,

df1$plh0037,

df1$plh0038,

df1$plh0039,

df1$plh0040,

df1$plh0162,

df1$plh0171,

df1$plh0174,

df1$plh0176,

df1$plh0178,

df1$plh0193,

df1$plh0195,

df1$plh0204_h,

df1$plh0335,

df1$pli0043_h,

df1$pli0046,

df1$pli0049_h,

df1$pli0051), ]

#non-response analysis

#generate dataframe with gender variables only

#n = 19426

#missings by sex

table(df_sample2018$sex)

table(df_pca$sex)

1-(8443/14536)

1-(10983/15684)

#missings are different for men and women

#> 8443/14536

#[1] 0.5808338

#> 10983/15684

#[1] 0.7002678

14536/(14536+15684)

8443/(8443+10983)

10983/(8443+10983)

#proportions

frq(df_sample2018$sex)

frq(df_pca$sex)

#----------------------------------------------------------#

#COMPOSITE GENDER MEASURE

library(sjmisc)

library(dplyr)

library(tidyverse)

library("nFactors")

library("psych")

library("psy")

library("lme4")

#generate dataframe for gender variables only

gender <- c("bip_197_01", "bip_197_02", "bip_197_03", "bip_197_04",

"bip_197_05", "bip_197_06", "bip_197_07", "bip_197_08",

"pgexpft", "pgexppt", "plb0018", "plh0033",

"plh0036", "plh0037", "plh0038", "plh0039",

"plh0040", "plh0162", "plh0174",

"plh0176", "plh0178",

"plh0193",

"plh0195", "plh0204_h", "plh0335", "pli0043_h",

"pli0046", "pli0049_h", "pli0051")

gv <- df_pca[gender]

head(gv)

glimpse(gv)

# descriptives for all gender variables - outliners

describe(gv)

dg <- tibble(sjmisc::descr(gv))

dg

write.csv(dg, "desc_gender.csv")

#PRINCIPAL COMPONENT ANALYSIS

#Check Voraussetzungen

#1: check n

#dataset is big enough

#2: bartlett test for dataframe - is there correlation between the items

#p-value should be significant - items are correlated

bartlett <- cortest.bartlett(gv)

bartlett

#p is 0 -> correlation between items

#3: Kaiser Meyer Olkin criteria and MSA values

#KMO value should be higher than 0.5

#MSA values for each item should also be higher than 0.5 (exclude items with lower values, if it isn't that important for the content)

kmo <- KMO(gv)

kmo

# get ordered MSAi

kmo$MSAi[order(kmo$MSAi)]

#mäßige Eignung zur FA 0.74 (0.8 ist gut)

#pgexppt is just above 0.51

#observe bip_197_04 +05 and pli0043_h - below 0.6

#above 0.8: bip_197_02 plh0335 bip_197_08 plh0176 plh0178 plh0033 bip_197_03

#plh0162 plh0193 plh0195 plh0187 plh0186 plh0038

#plh0185 plh0184 plh0174 plh0189 bip_197_01 plh0172

#4: anti-image correlation matrix

#5: For all variable pairs with a correlation coefficient greater than 0.80, we excluded one variable randomly

library(corrplot)

c <- cor(gv, method = "spearman", use = "complete.obs")

c

write.csv(c, "correlation matrix.csv")

cor1 <- corrplot(c, method = "number", type = "upper")

cor2 <- corrplot.mixed(c, lower.col = "black", number.cex = .7)

cor1

cor2

#no correlation greater than 0.80, no exclusion based on the correlation coefficients

sumgv <- summary(gv)

sumgv

write.csv(sumgv, "sumgv.csv")

#principal component analysis

pca1 <- princomp(gv, scores = TRUE, cor = TRUE)

pca1

summary(pca1)

#according to eigenvalues, 9 components with eigenvalues higher than 1, explain 60% of the variance

#need for all variables

#loadings of principal components

loadings <- as.table(loadings(pca1))

loadings

write.csv(loadings, "loadings.csv")

loadings(pca1, cutoff = 0.25)

loadings(pca1, cutoff = 0.5, sort = TRUE)

#scree plot of eigenvalues

plot(pca1)

screeplot(pca1, type = "line", main = "Scree Plot")

#biplot of score variables

#biplot(pca1)

#----------------------------------------------------------#

#LOGISTIC REGRESSION TO CALCULATE GENDER SCORE

#to reduce number of variables, sex = outcome

library("DescTools")

#calculate with only with gender variables

gender1 <- c("pid", "cid", "sexr",

"bip_197_01", "bip_197_02", "bip_197_03", "bip_197_04",

"bip_197_05", "bip_197_06", "bip_197_07", "bip_197_08",

"pgexpft", "pgexppt", "plb0018", "plh0033",

"plh0036", "plh0037", "plh0038", "plh0039",

"plh0040", "plh0162", "plh0174",

"plh0176", "plh0178",

"plh0193",

"plh0195", "plh0204_h", "plh0335", "pli0043_h",

"pli0046", "pli0049_h", "pli0051")

gvr <- df_pca[gender1]

head(gvr)

glimpse(gvr)

gvr$pid <- as.factor(gvr$pid)

gvr$cid <- as.factor(gvr$cid)

length(unique(gvr$cid))

#multilevel logistic regression to control for household structure

m1 <- glmer(sexr ~ 1 + (1 | cid),

data = gvr,

family = binomial(link='logit'),

control = glmerControl(optimizer = "bobyqa"),

nAGQ = 0)

m1

summary(m1)

#AIC BIC logLik deviance df.resid

#26601.1 26616.8

#all variables - purely statistical selection

m2 <- glmer(sexr ~ bip_197_01 + bip_197_02 + bip_197_03 + bip_197_04 +

bip_197_05 + bip_197_06 + bip_197_07 + bip_197_08 +

pgexppt + pgexpft + plb0018 + plh0033 + plh0036 + plh0037 + plh0038 +

plh0039 + plh0040 + plh0162 + plh0174 + plh0176 +

plh0178 + plh0193 +

plh0195 + plh0204_h + plh0335 + pli0043_h + pli0046 + pli0049_h +

pli0051 + (1 | cid),

data = gvr,

family = binomial(link='logit'),

control = glmerControl(optimizer = "bobyqa"),

nAGQ = 0)

summary(m2)

#AIC BIC logLik deviance df.resid

#15935.6 16179.7

#exclude based on p-values in decending order (below 0.01)

#plh0033

#pli0046

#plh0335

#plh0176

#plh0036

#plh0193

#plh0178

#plh0195

#below 0.01

#bip_197_06

#plh0037

m3 <- glmer(sexr ~ bip_197_01 + bip_197_02 + bip_197_03 + bip_197_04 +

bip_197_05 + bip_197_07 + bip_197_08 +

pgexppt + pgexpft + plb0018 + plh0038 +

plh0039 + plh0040 + plh0162 + plh0174 +

plh0204_h + pli0043_h + pli0049_h +

pli0051 + (1 | cid),

data = gvr,

family = binomial(link='logit'),

control = glmerControl(optimizer = "bobyqa"),

nAGQ = 0)

summary(m3)

#AIC BIC logLik deviance df.resid

#15936.3 16101.7

m3_1 <- glm(sexr ~ bip_197_01 + bip_197_02 + bip_197_03 + bip_197_04 +

bip_197_05 + bip_197_07 + bip_197_08 +

pgexppt + pgexpft + plb0018 + plh0038 +

plh0039 + plh0040 + plh0162 + plh0174 +

plh0204_h + pli0043_h + pli0049_h +

pli0051,

data = gvr,

family = binomial(link='logit'))

Cstat(m3_1)

#--------------------------------------------------------------#

#Play around with aic and variables

aggregate(gvr$pgexpft, by=list(gvr$sexr), FUN=mean)

aggregate(gvr$pgexppt, by=list(gvr$sexr), FUN=mean)

aggregate(gvr$plh0039, by=list(gvr$sexr), FUN=mean)

aggregate(gvr$plh0162, by=list(gvr$sexr), FUN=mean)

aggregate(gvr$plh0174, by=list(gvr$sexr), FUN=mean)

aggregate(gvr$bip_197_08, by=list(gvr$sexr), FUN=mean)

aggregate(gvr$bip_197_03, by=list(gvr$sexr), FUN=mean)

aggregate(gvr$plh0204_h, by=list(gvr$sexr), FUN=mean)

aggregate(gvr$pli0043_h, by=list(gvr$sexr), FUN=mean)

aggregate(gvr$pli0049_h, by=list(gvr$sexr), FUN=mean)

aggregate(gvr$pli0051, by=list(gvr$sexr), FUN=mean)

#least significant

#bip_197_02 / 07 /05 - exclusion only minimally increases AIC

#highly significant:

#bip_197_03 #bip_197_08 #pgexppt #pgexpft #plh0039 #plh0162 #plh0174

#plh0204_h #pli0043_h #pli0049_h #pli0051

m3play <- glmer(sexr ~ bip_197_01 + bip_197_02 + bip_197_03 + bip_197_04 +

bip_197_05 + bip_197_07 + bip_197_08 +

pgexppt + pgexpft + plb0018 + plh0038 +

plh0039 + plh0040 + plh0162 + plh0174 +

plh0204_h + pli0043_h + pli0049_h +

pli0051 + (1 | cid),

data = gvr,

family = binomial(link='logit'),

control = glmerControl(optimizer = "bobyqa"),

nAGQ = 0)

summary(m3play)

#------------#

#exclude randomly exclude variables that are strongly associated with others - to only include one of them

#base m2

#correlations above 0.7

#I4&5

#bip_197_04 aic 15951.3

#bip_197_05 aic 15945.4

#exclude bip_197_04

mcor1a <- glmer(sexr ~ bip_197_01 + bip_197_02 + bip_197_03 +

bip_197_05 + bip_197_06 + bip_197_07 + bip_197_08 +

pgexppt + pgexpft + plb0018 + plh0033 + plh0036 + plh0037 + plh0038 +

plh0039 + plh0040 + plh0162 + plh0174 + plh0176 +

plh0178 + plh0193 +

plh0195 + plh0204_h + plh0335 + pli0043_h + pli0046 + pli0049_h +

pli0051 + (1 | cid),

data = gvr,

family = binomial(link='logit'),

control = glmerControl(optimizer = "bobyqa"),

nAGQ = 0)

summary(mcor1a)

#exclude bip_197_05

mcor1b <- glmer(sexr ~ bip_197_01 + bip_197_02 + bip_197_03 + bip_197_04 +

bip_197_06 + bip_197_07 + bip_197_08 +

pgexppt + pgexpft + plb0018 + plh0033 + plh0036 + plh0037 + plh0038 +

plh0039 + plh0040 + plh0162 + plh0174 + plh0176 +

plh0178 + plh0193 +

plh0195 + plh0204_h + plh0335 + pli0043_h + pli0046 + pli0049_h +

pli0051 + (1 | cid),

data = gvr,

family = binomial(link='logit'),

control = glmerControl(optimizer = "bobyqa"),

nAGQ = 0)

summary(mcor1b)

#I13&14

#exclude plh0038

mcor2a <- glmer(sexr ~ bip_197_01 + bip_197_02 + bip_197_03 + bip_197_04 +

bip_197_05 + bip_197_06 + bip_197_07 + bip_197_08 +

pgexppt + pgexpft + plb0018 + plh0033 + plh0036 + plh0037 +

plh0039 + plh0040 + plh0162 + plh0174 + plh0176 +

plh0178 + plh0193 +

plh0195 + plh0204_h + plh0335 + pli0043_h + pli0046 + pli0049_h +

pli0051 + (1 | cid),

data = gvr,

family = binomial(link='logit'),

control = glmerControl(optimizer = "bobyqa"),

nAGQ = 0)

summary(mcor2a)

#exclude plh0037 aic

mcor2b <- glmer(sexr ~ bip_197_01 + bip_197_02 + bip_197_03 + bip_197_04 +

bip_197_05 + bip_197_06 + bip_197_07 + bip_197_08 +

pgexppt + pgexpft + plb0018 + plh0033 + plh0036 + plh0038 +

plh0039 + plh0040 + plh0162 + plh0174 + plh0176 +

plh0178 + plh0193 +

plh0195 + plh0204_h + plh0335 + pli0043_h + pli0046 + pli0049_h +

pli0051 + (1 | cid),

data = gvr,

family = binomial(link='logit'),

control = glmerControl(optimizer = "bobyqa"),

nAGQ = 0)

summary(mcor2b)

#both cor models combined (bip05 & plh0037 excluded)

mcor3 <- glmer(sexr ~ bip_197_01 + bip_197_02 + bip_197_03 + bip_197_04 +

bip_197_06 + bip_197_07 + bip_197_08 +

pgexppt + pgexpft + plb0018 + plh0033 + plh0036 + plh0038 +

plh0039 + plh0040 + plh0162 + plh0174 + plh0176 +

plh0178 + plh0193 +

plh0195 + plh0204_h + plh0335 + pli0043_h + pli0046 + pli0049_h +

pli0051 + (1 | cid),

data = gvr,

family = binomial(link='logit'),

control = glmerControl(optimizer = "bobyqa"),

nAGQ = 0)

summary(mcor3)

#aic 15943.0

#stepwise exclusion

#plh0033

#pli0046

#plh0335

#plh0176

#plh0193

#plh0178

#below 0.01

#plh0195

#plh0036

#bip_197_06

mcor3a <- glmer(sexr ~ bip_197_01 + bip_197_02 + bip_197_03 + bip_197_04 +

bip_197_07 + bip_197_08 +

pgexppt + pgexpft + plb0018 + plh0038 +

plh0039 + plh0040 + plh0162 + plh0174 +

plh0204_h + pli0043_h + pli0049_h +

pli0051 + (1 | cid),

data = gvr,

family = binomial(link='logit'),

control = glmerControl(optimizer = "bobyqa"),

nAGQ = 0)

summary(mcor3a)

mcor3a_1 <- glm(sexr ~ bip_197_01 + bip_197_02 + bip_197_03 + bip_197_04 +

bip_197_07 + bip_197_08 +

pgexppt + pgexpft + plb0018 + plh0038 +

plh0039 + plh0040 + plh0162 + plh0174 +

plh0204_h + pli0043_h + pli0049_h +

pli0051,

data = gvr,

family = binomial(link='logit'))

Cstat(mcor3a_1)

#------------#

#content-wise reduction

#pgexppt, pgexpft (einzeln oder gemeinsam), - schlechteres aic

#exclude highly correlated (bip 05 & 0037), but all other variables

#exclude pgexppt

mpt <- glmer(sexr ~ bip_197_01 + bip_197_02 + bip_197_03 + bip_197_04 +

bip_197_06 + bip_197_07 + bip_197_08 +

pgexpft + plb0018 + plh0033 + plh0036 + plh0038 +

plh0039 + plh0040 + plh0162 + plh0174 + plh0176 +

plh0178 + plh0193 +

plh0195 + plh0204_h + plh0335 + pli0043_h + pli0046 + pli0049_h +

pli0051 + (1 | cid),

data = gvr,

family = binomial(link='logit'),

control = glmerControl(optimizer = "bobyqa"),

nAGQ = 0)

summary(mpt)

#exclude pgexpft

mft <- glmer(sexr ~ bip_197_01 + bip_197_02 + bip_197_03 + bip_197_04 +

bip_197_06 + bip_197_07 + bip_197_08 +

pgexppt + plb0018 + plh0033 + plh0036 + plh0038 +

plh0039 + plh0040 + plh0162 + plh0174 + plh0176 +

plh0178 + plh0193 +

plh0195 + plh0204_h + plh0335 + pli0043_h + pli0046 + pli0049_h +

pli0051 + (1 | cid),

data = gvr,

family = binomial(link='logit'),

control = glmerControl(optimizer = "bobyqa"),

nAGQ = 0)

summary(mft)

#better aic when pgexpft is excluded

#stepwise exclusion

#bip_197_02

#bip_197_06

#plh0193

#pli0046

#plh0195

#below 0.1

#plh0178

#plb0018

#plh0335

#plh0040

mft1 <- glmer(sexr ~ bip_197_01 + bip_197_03 + bip_197_04 +

bip_197_07 + bip_197_08 +

pgexppt + plh0033 + plh0036 + plh0038 +

plh0039 + plh0162 + plh0174 + plh0176 +

plh0204_h + pli0043_h + pli0049_h +

pli0051 + (1 | cid),

data = gvr,

family = binomial(link='logit'),

control = glmerControl(optimizer = "bobyqa"),

nAGQ = 0)

summary(mft1)

mft1_1 <- glm(sexr ~ bip_197_01 + bip_197_03 + bip_197_04 +

bip_197_07 + bip_197_08 +

pgexppt + plh0033 + plh0036 + plh0038 +

plh0039 + plh0162 + plh0174 + plh0176 +

plh0204_h + pli0043_h + pli0049_h +

pli0051,

data = gvr,

family = binomial(link='logit'))

Cstat(mft1_1)

#------------------------------------------------#

#exclude more variables a priori - content based selection

#base m2

#exclude correlation pairs - bip05, pl0037

#exclude pgexpft (pgexppt has better aic)

#content-wise exclusion:

#bip 03

#plh0036

#plh0038

#plh0039

#plh0162

#plh193

#plh0195

mc1 <- glmer(sexr ~ bip_197_01 + bip_197_02 + bip_197_04 +

bip_197_06 + bip_197_07 + bip_197_08 +

pgexppt + plb0018 + plh0033 +

plh0040 + plh0174 + plh0176 +

plh0178 +

plh0204_h + plh0335 + pli0043_h + pli0046 + pli0049_h +

pli0051 + (1 | cid),

data = gvr,

family = binomial(link='logit'),

control = glmerControl(optimizer = "bobyqa"),

nAGQ = 0)

summary(mc1)

#stepwise exclusion

#plh0176

#bip_197_06

#pli0046

#bip_197_02

#plh0033

#plh0178

mc2 <- glmer(sexr ~ bip_197_01 + bip_197_04 +

bip_197_07 + bip_197_08 +

pgexppt + plb0018 +

plh0040 + plh0174 +

plh0204_h + plh0335 + pli0043_h + pli0049_h +

pli0051 + (1 | cid),

data = gvr,

family = binomial(link='logit'),

control = glmerControl(optimizer = "bobyqa"),

nAGQ = 0)

summary(mc2)

mc2_1 <- glm(sexr ~ bip_197_01 + bip_197_04 +

bip_197_07 + bip_197_08 +

pgexppt + plb0018 +

plh0040 + plh0174 +

plh0204_h + plh0335 + pli0043_h + pli0049_h +

pli0051,

data = gvr,

family = binomial(link='logit'))

Cstat(mc2_1)

#same as mc1, but retain plh0036 - climate and plh0039 terrorism

mc3 <- glmer(sexr ~ bip_197_01 + bip_197_02 + bip_197_04 +

bip_197_06 + bip_197_07 + bip_197_08 +

pgexppt + plb0018 + plh0033 + plh0036 + plh0039 +

plh0040 + plh0174 + plh0176 +

plh0178 +

plh0204_h + plh0335 + pli0043_h + pli0046 + pli0049_h +

pli0051 + (1 | cid),

data = gvr,

family = binomial(link='logit'),

control = glmerControl(optimizer = "bobyqa"),

nAGQ = 0)

summary(mc3)

#stepwise exclusion

#plh0176

#bip_197_06

#pli0046

#plh0033

#bip_197_02

#plh0178

#plh0036

#plb0018

mc4 <- glmer(sexr ~ bip_197_01 + bip_197_04 +

bip_197_07 + bip_197_08 +

pgexppt + plh0039 +

plh0040 + plh0174 +

plh0204_h + plh0335 + pli0043_h + pli0049_h +

pli0051 + (1 | cid),

data = gvr,

family = binomial(link='logit'),

control = glmerControl(optimizer = "bobyqa"),

nAGQ = 0)

summary(mc4)

mc4_1 <- glm(sexr ~ bip_197_01 + bip_197_04 +

bip_197_07 + bip_197_08 +

pgexppt + plh0039 +

plh0040 + plh0174 +

plh0204_h + plh0335 + pli0043_h + pli0049_h +

pli0051,

data = gvr,

family = binomial(link='logit'))

Cstat(mc4_1)

#----------------------------------------------------------#

#COMPARE THE DIFFERENT MODELS

#check the different models

summary(m3)

summary(mcor3a)

summary(mft1)

summary(mc2)

summary(mc4)

##GENERATE THE SCORE

library("MatchIt")

glimpse(df_pca)

#dataset with variables included in the analysis

analysis <- c("pid", "sexr", "cid",

"age", "agecr", "edur",

"incr",

"germstate", "eastr",

"parentr", "hhchild", "single", "livwpart", "sexor",

"migbackr", "migbackbin", "germbornr", "resistatr",

"corigin_big6", "regionc", "continentc",

"immiyear", "imyear5", "imyearcatr", "imgrpr",

"mcs",

"pcs",

"bip_197_01", "bip_197_02", "bip_197_03", "bip_197_04",

"bip_197_05", "bip_197_06", "bip_197_07", "bip_197_08",

"pgexpft", "pgexppt", "plb0018", "plh0033",

"plh0036", "plh0037", "plh0038", "plh0039",

"plh0040", "plh0162", "plh0174",

"plh0176", "plh0178",

"plh0193",

"plh0195", "plh0204_h", "plh0335", "pli0043_h",

"pli0046", "pli0049_h", "pli0051")

df_analysis <- df_pca[analysis]

#Compute Propensity score

#Attach the predicted propensity score to the datafile

df_analysis$M0 <- predict(m3, type = "response")

df_analysis$M1 <- predict(mcor3a, type = "response")

df_analysis$M2 <- predict(mft1, type = "response")

df_analysis$M3 <- predict(mc4, type = "response")

df_analysis$M4 <- predict(mc2, type = "response")

glimpse(df_analysis)

#compare the different scores

summary(df_analysis$M0)

summary(df_analysis$M1)

summary(df_analysis$M2)

summary(df_analysis$M3)

summary(df_analysis$M4)

ps_sum <- df_analysis %>%

select(M0, M1, M2, M3, M4) %>%

psych::describe(quant=c(.25,.75)) %>%

as_tibble(rownames="rowname")

write.csv(ps_sum, "summary psvalues")

#very similiar distributions

aggregate(df_analysis$M0, by=list(df_analysis$sexr), FUN=summary)

aggregate(df_analysis$M1, by=list(df_analysis$sexr), FUN=summary)

aggregate(df_analysis$M2, by=list(df_analysis$sexr), FUN=summary)

aggregate(df_analysis$M3, by=list(df_analysis$sexr), FUN=summary)

aggregate(df_analysis$M4, by=list(df_analysis$sexr), FUN=summary)

psvalues <- c("pid", "sexr", "M0", "M1", "M2", "M3", "M4")

df_ps <- df_analysis[psvalues]

library(summarytools)

ps_sum_bysex <- stby(data = df_ps,

INDICES = df_ps$sexr,

FUN = descr, stats = "common", transpose = TRUE)

ps_sum_bysex

# Plot

library("RColorBrewer")

display.brewer.all()

library(wesanderson)

library(reshape2)

df_psl <- melt(df_ps, id.vars = c("pid", "sexr"))

glimpse(df_psl)

dist_scores <- ggplot(df_psl,aes(x=value, fill=variable)) +

geom_density(alpha=0.25) +

xlab("Different versions of composite gender scores") +

ggtitle("Distributions of the composite gender scores") +

theme_classic() +

theme(legend.title=element_blank()) +

scale_fill_brewer(palette = "Greys")

dist_scores

box_scores <- ggplot(df_psl,aes(x=variable, y=value, fill=variable)) +

geom_boxplot() +

xlab("Different versions of composite gender scores") +

ggtitle("Distributions of the composite gender scores") +

theme_classic() +

theme(legend.title=element_blank()) +

scale_fill_brewer(palette = "Greys")

box_scores

dist_scores_sex <- ggplot(df_psl,aes(x=value, fill=variable)) +

geom_density(alpha=0.25) +

xlab("Different versions of composite gender scores") +

ggtitle("Distribution of the composite gender scores\nby sex assigned at birth") +

theme_classic() +

theme(legend.title=element_blank()) +

scale_fill_brewer(palette = "Greys") +

facet_grid(. ~ sexr)

box_scores_sex <- ggplot(df_psl,aes(x=variable, y=value, fill=variable)) +

geom_boxplot() +

xlab("Different versions of composite gender scores") +

ggtitle("Distribution of the composite gender scores\nby sex assigned at birth") +

theme_classic() +

theme(legend.title=element_blank()) +

scale_fill_brewer(palette = "Greys") +

facet_grid(. ~ sexr)

dfs_analysis %>% ggplot(aes(x = genderscore, linetype = sexr)) +

geom_density() +

ylab("Density") + xlab("Gendered social practices") +

theme_classic() +

theme(axis.text.x = element_blank()) +

theme(legend.title=element_blank())

dist_scores_sex

box_scores_sex

pdf("Comparison of different scores.pdf")

print(dist_scores) # Plot 1 --> in the first page of PDF

print(box_scores) # Plot 2 ---> in the second page of the PDF

print(dist_scores_sex)

print(box_scores_sex)

dev.off()

#----------------------------------------------------------#

#Decide for a score

#mc4 --> balance between content-based and purely statistical decision

mc4 <- glmer(sexr ~ bip_197_01 + bip_197_04 +

bip_197_07 + bip_197_08 +

pgexppt + plh0039 +

plh0040 + plh0174 +

plh0204_h + plh0335 + pli0043_h + pli0049_h +

pli0051 + (1 | cid),

data = gvr,

family = binomial(link='logit'),

control = glmerControl(optimizer = "bobyqa"),

nAGQ = 0)

summary(mc4)

#CALCULATE GENDER SCORE FOR NEW SAMPLE

#complete cases only for the variables included

#bip_197_01 + bip_197_04 + bip_197_07 + bip_197_08 + pgexppt + plh0039 +

# plh0040 + plh0174 + plh0204_h + plh0335 + pli0043_h + pli0049_h + pli0051

df_sample <- df_sample2018[complete.cases(df_sample2018$bip_197_01,

df_sample2018$bip_197_04,

df_sample2018$bip_197_07,

df_sample2018$bip_197_08,

df_sample2018$pgexppt,

df_sample2018$plh0039,

df_sample2018$plh0040,

df_sample2018$plh0174,

df_sample2018$plh0204_h,

df_sample2018$plh0335,

df_sample2018$pli0043_h,

df_sample2018$pli0049_h,

df_sample2018$pli0051), ]

glimpse(df_sample)

glimpse(df_analysis)

summary(df_sample)

#dataset with variables included in the analysis

analysis1 <- c("pid", "sexr", "cid",

"age", "agecr", "edur",

"incr",

"germstate", "eastr", "sexor",

"single", "livwpart",

"migbackr", "migbackbin", "germbornr", "resistatr",

"corigin_big6", "regionc", "continentc",

"immiyear", "imyear5", "imyearcatr", "imgrpr",

"mcs",

"pcs",

"bip_197_01", "bip_197_04",

"bip_197_07", "bip_197_08",

"pgexppt", "plh0039",

"plh0040", "plh0174",

"plh0204_h", "plh0335", "pli0043_h", "pli0049_h",

"pli0051", "biopr", "socpr", "parent_allr")

dfs_analysis <- df_sample[analysis1]

#apply labels

library(expss)

dfs_analysis <- apply_labels(dfs_analysis, sexr = "Sex assigned at birth",

age = "Age in years", agecr = "Age in years (categorised)",

inc = "Pre-government household income",

inc3r = "Pre-government household income (categorised)",

eastr = "State of residence - East vs. West-Germany",

sexor = "Assumed current sexual attraction",

edur = "Educational attainment according to CASMIN 2011 classification (merged groups)",

parentr = "Parental status", hhchild = "Household with at least 1 child below the age of 14 years",

single = "Marital status", livwpart = "Living together with a partner",

migbackr = "Migration background",

germbornr = "Born in Germany",

corigin_big6 = "Countries of origin (the 6 countries most frequent in the SOEP 2018 sample)",

regionc = "Region of origin", continentc = "Continent of origin",

immiyear = "Year moved to Germany",

imyear5 = "Year of immigration (in 5-year groups)",

imyearcatr = "Year of immigration before and after 2014",

mcs = "Mental health score (SF-12_v2)",

pcs = "Physical health score (SF-12_v2)")

glimpse(dfs_analysis)

#DISTRIBUTION OF GENDER SCORE

cgs <- glmer(sexr ~ bip_197_01 + bip_197_04 +

bip_197_07 + bip_197_08 +

pgexppt + plh0039 +

plh0040 + plh0174 +

plh0204_h + plh0335 + pli0043_h + pli0049_h +

pli0051 + (1 | cid),

data = dfs_analysis,

family = binomial(link='logit'),

control = glmerControl(optimizer = "bobyqa"),

nAGQ = 0)

summary(cgs)

summary(dfs_analysis$bip_197_01)

summary(dfs_analysis$bip_197_04)

summary(dfs_analysis$pgexppt)

summary(dfs_analysis$plh0039)

summary(dfs_analysis$plh0040)

summary(dfs_analysis$plh0174)

summary(dfs_analysis$plh0204_h)

summary(dfs_analysis$pli0049_h)

dfs_analysis$genderscore <- predict(cgs, type = "response")

glimpse(dfs_analysis)

#recode norms variables

glimpse(dfs_analysis)

dfs_analysis <- dfs_analysis %>%

mutate(bip_197_01_Reversed = 8 - bip_197_01,

bip_197_04_Reversed = 8 - bip_197_04)

glimpse(dfs_analysis)

cgs <- glmer(sexr ~ bip_197_01_Reversed + bip_197_04_Reversed +

bip_197_07 + bip_197_08 +

pgexppt + plh0039 +

plh0040 + plh0174 +

plh0204_h + plh0335 + pli0043_h + pli0049_h +

pli0051 + (1 | cid),

data = dfs_analysis,

family = binomial(link='logit'),

control = glmerControl(optimizer = "bobyqa"),

nAGQ = 0)

summary(cgs)

#check model fit

library(tidyverse)

library(broom)

library(car)

## estimate the probability (p) of being a women

probabilities <- predict(cgs, type = "response")

predicted.classes <- ifelse(probabilities > 0.5, "fem", "mal")

head(predicted.classes)

#linearity assumption

#remove qualitative variables from dataset

glimpse(dfs_analysis)

mydata <- dfs_analysis %>%

dplyr::select_if(is.numeric)

glimpse(mydata)

mydata = subset(mydata, select = -c(mcs, pcs, immiyear, nonresp, genderscore))

predictors <- colnames(mydata)

# Bind the logit and tidying the data for plot

mydata <- mydata %>%

mutate(logit = log(probabilities/(1-probabilities))) %>%

gather(key = "predictors", value = "predictor.value", -logit)

#create the scatter plots

ggplot(mydata, aes(logit, predictor.value))+

geom_point(size = 0.5, alpha = 0.5) +

geom_smooth(method = "loess") +

theme_bw() +

facet_wrap(~predictors, scales = "free_y")

#influential values

#extreme values

plot(cgs, which = 4, id.n = 3)

#standardized residuals to check whether influential

# Extract model results

model.data <- augment(cgs) %>%

mutate(index = 1:n())

#The data for the top 3 largest values, according to the Cook’s distance,

#can be displayed as follow:

model.data %>% top_n(3, .cooksd)

#Plot the standardized residuals:

ggplot(model.data, aes(index, .std.resid)) +

geom_point(aes(color = diabetes), alpha = .5) +

theme_bw()

#Filter potential influential data points with abs(.std.res) > 3:

model.data %>%

filter(abs(.std.resid) > 3)

#multicollinearity test

#check_collinearity(cgs, verbose = TRUE)

??check_collinearity

vif(cgs) # all VIF between 1.0 and 1.63

table(dfs_analysis$agecr, dfs_analysis$imyear10)

#descriptive statistics (to add sample size to figures)

frq(dfs_analysis)

frq_summary <- dfSummary(dfs_analysis)

view(frq_summary, file = "frq_summary.html")

dffrequency <- c("sexr", "agecr", "edur","incr",

"eastr", "sexor",

"parentr", "hhchild", "single", "livwpart",

"migbackr", "migbackbin", "germbornr", "resistatr",

"corigin_big6", "regionc", "continentc",

"imyear5", "imyearcatr", "imgrpr")

dffreq <- dfs_analysis[dffrequency]

frq(dffreq)

#describe genderscore in more detail

summary(dfs_analysis$genderscore)

frq(dfs_analysis$sexr)

dfs_w <- subset(dfs_analysis, sexr=="female")

dfs_m <- subset(dfs_analysis, sexr=="male")

quantile(dfs_analysis$genderscore, probs = seq(0, 1, 1/3), na.rm = TRUE)

quantile(dfs_w$genderscore, probs = seq(0, 1, 1/3), na.rm = TRUE)

quantile(dfs_m$genderscore, probs = seq(0, 1, 1/3), na.rm = TRUE)

summarytools::descr(dfs_analysis$genderscore)

summarytools::descr(dfs_w$genderscore)

summarytools::descr(dfs_m$genderscore)

library(summarytools)

aggregate(dfs_w$genderscore, list(dfs_analysis$agecr), mean)

aggregate(dfs_w$genderscore, list(dfs_analysis$agecr), sd)

?summarytools::descr

data("dfs_analysis")

#recode stratifying variables to give n in subgroups

#SEX sex

sjmisc::frq(dfs_analysis$sexr)

dfs_analysis$sexrn <- dplyr::recode_factor(dfs_analysis$sexr,

'male' = "male (n = 8902)", 'female' = "female (n=11865)",

as.default = NA_character_)

table(dfs_analysis$sexrn)

#AGE d11101

frq(dfs_analysis$agecr)

dfs_analysis$agecrn <- dplyr::recode_factor(dfs_analysis$agecr,

'18-30 years' = "18-30 years \n(n=3624)",

'31-45 years' = "31-45 years \n(n=5360)",

'46-60 years' = "46-60 years \n(n=6375)",

'61-75+ years' = "61-75+ years \n(n=5408)",

.default = NA_character_)

#EDUCATIONAL ATTAINMENT

frq(dfs_analysis$edur)

dfs_analysis$edurn <- dplyr::recode_factor(dfs_analysis$edur, 'low educational attainment' = "low (n=6122)",

'middle educational attainment' = "middle (n=8764)",

'high educational attainment' = "high (n=5096)",

.default = NA_character_)

#HOUSEHOLD INCOME

#Household Pre-Government Income i1110118 -> need to extract it from pequiv

frq(dfs_analysis$incr)

dfs_analysis$incrn <- dplyr::recode_factor(dfs_analysis$incr, 'low monthly household income' = "lowest income quintil \n(n=2650)",

'middle monthly household income' = "middle income quintiles \n(n=13394)",

'high monthly household income' = "highest income quintile \n(n=4723)",

.default = NA_character_)

#STATE OF RESIDENCE l11101

frq(dfs_analysis$eastr)

dfs_analysis$eastrn <- dplyr::recode_factor(dfs_analysis$eastr,

'West' = "West Germany \n(n=15875)",

'East' = "East Germany \n(n=4892)")

#sexual orientation

frq(dfs_analysis$sexor)

dfs_analysis$sexorn <- dplyr::recode_factor(dfs_analysis$sexor, 'probably heterosexual' = "not living in a \nsame sex/gender \npartnership (n=17401)",

'probably bi/homosexual' = "living in a same \nsex/gender partnership \n(n=255)",

'insufficient information' = "insufficient information \n(n=3111)")

frq(dfs_analysis$sexorn)

#Parenthood

frq(dfs_analysis$biopr)

dfs_analysis$bioprn <- recode_factor(dfs_analysis$biopr, 'no biological children' = "no children (n=5966)",

'at least 1 biological child' = "at least one biological child (n=14801)")

#Parenthood & cohabitation status

frq(dfs_analysis$parent_allr)

dfs_analysis$parent_allrn <- recode_factor(dfs_analysis$parent_allr,

'no children' = "no children (n=5966)",

'parents not cohabitating with child(ren)' = "parents not cohabitating \nwith child(ren) (n=6173)",

'parents cohabitating with child(ren)' = 'parents cohabitating with \nchild(ren) (n=8628)')

#marital status

frq(dfs_analysis$single)

dfs_analysis$singlen <- recode_factor(dfs_analysis$single, 'single' = "single (n=8983)",

'in partnership' = "in a partnership (n=11722)")

frq(dfs_analysis$livwpart)

dfs_analysis$livwpartn <- recode_factor(dfs_analysis$livwpart,

'single or not living with their partner' = "single or not living \nwith their partner (n=8993)",

'living with a partner' = "living with their \npartner (n=11712)")

#MIGRATION RELATED VARIABLES

#MIGRATION BACKGROUND migback

frq(dfs_analysis$migbackr)

dfs_analysis$migbackrn <- dplyr::recode_factor(dfs_analysis$migbackr,

'no migration background' = "no migration background (n=15815)",

'direct migration background' = "direct migration background(n=3408)",

'indirect migration background' = "indirect migration background (n=1544)",

as.default = NA_character_)

#COUNTRY OF ORIGIN corigin corigin

frq(dfs_analysis$corigin_big6)

dfs_analysis$corigin_big6n <- recode_factor(dfs_analysis$corigin_big6,

'Germany' = "Germany \n(n=17359)",

'Poland' = "Poland \n(n=463)",

'Russia' = "Russia \n(n=382)",

'Kazakhstan' = "Kazakhstan \n(n=330)",

'Turkey' = "Turkey \n(n=271)",

'Rumania' = "Romania \n(n=250)",

as.default = NA_character_)

#by region

frq(dfs_analysis$regionc)

dfs_analysis$regioncn <- dplyr::recode_factor(dfs_analysis$regionc,

'Eastern Europe' = "Eastern Europe \n(n=1696)",

'Western Europe' = "Western Europe \n(n=455)",

'Central Asia' = "Central Asia \n(n=410)",

'Middle East' = "Middle East \n(n=482)",

as.default = NA_character_)

table(dfs_analysis$regioncn)

is.na(dfs_analysis$regioncn) <- dfs_analysis$regioncn == "Germany"

?droplevels

#BORN IN GERMANY

frq(dfs_analysis$germbornr)

dfs_analysis$germbornrn <- dplyr::recode_factor(dfs_analysis$germbornr,

'born in Germany or immigr.<1950' = "born in Germany or \nimmigr. <1950 (n=17359)",

'not born in Germany' = "not born in Germany \n(n=3408)",

as.default = NA_character_)

#RESIDENCE STATUS

frq(dfs_analysis$resistatr)

dfs_analysis$resistatrn <- dplyr::recode_factor(dfs_analysis$resistat,

'Unlimited' = "unlimited \n(n=998)", 'Temporary' = "temporary \n(n=450)",

as.default = NA_character_)

#LENGTH OF STAY IN GERMANY IN YEARS immiyear

dfs_analysis <- mutate(dfs_analysis, imyear10 = ifelse(immiyear %in% 2009:2018, 1,

ifelse(immiyear %in% 1950:2008, 0, NA)))

table(dfs_analysis$imyear10, exclude = NULL)

dfs_analysis$imyear10 <- dplyr::recode_factor(dfs_analysis$imyear10,

'0' = "before 2009 \n(n=2576)", '1' = "2009 to 2018 \n(n=781)")

frq(dfs_analysis$imyear10)

#IMMIGRANT GROUP

frq(dfs_analysis$imgrpr)

dfs_analysis$imgrprn <- dplyr::recode_factor(dfs_analysis$imgrpr,

'Person of German descent from Eastern Europe' = "person of German descent \nfrom Eastern Europe (n=936)",

'Citizen of EU country (up to 2009 EC)' = "citizen of EU country \n(up to 2009 EC) (n=759)",

'Asylum seeker, refugee' = "asylum seeker, refugee \n(n=384)",

'Other foreigner' = "other foreigner \n(n=739)",

.default = NA_character_)

frq(dfs_analysis$imgrprn)

library(openxlsx)

write.xlsx(dfs_analysis, 'dfs_analysis.xlsx')

#GGPLOTS -VISUALISATION OF GENDER SCORE DISTRIBUTION

#by sex

library(ggplot2)

library(EnvStats)

library(dplyr)

library(tidyr)

library(ggridges)

library(ggpubr)

install.packages()

#first histogramm by sex

f1 <- dfs_analysis %>% ggplot(aes(x = genderscore, linetype = sexr)) +

geom_density() +

ylab("Density") + xlab("Gendered social practices") +

theme_classic() +

theme(axis.text.x = element_blank()) +

theme(legend.title=element_blank())

f1

f_basic <- f1 + stat_central_tendency(type = "median")

f_basic

medians <- aggregate(genderscore ~ sexr, dfs_analysis, median)

dfs_analysis %>% ggplot(aes(x = genderscore, linetype = sexr)) +

geom_density() +

ylab("Density") + xlab("Gendered social practices") +

theme_classic() +

theme(axis.text.x = element_blank()) +

theme(legend.title=element_blank()) +

geom_text(data = medians, aes(label=round(genderscore, 1),

y = genderscore - 0.2), hjust = -0.5, size = 3) +

stat_central_tendency(type = "median")#adds median labels

#by migration characteristics

#country of birth

f_cobi <- dfs_analysis %>%

ggplot(aes(x = genderscore, y= germbornrn, linetype = sexr)) +

geom_density_ridges(quantile_lines = TRUE, quantiles = 0.5, scale = 1, alpha = 0.01) +

ggtitle(label = "Country of birth") +

ylab("Density") + xlab("Gendered social practices") +

theme_classic() +

theme(axis.text.x = element_blank()) +

theme(legend.title=element_blank())

f_cobi

#CROSS CLASSIFICATIONS

f3 <- dfs_analysis %>% ggplot(aes(x = genderscore, linetype = sexr)) +

geom_density() +

ylab("Density") + xlab("Gendered social practices") +

theme_classic() +

theme(axis.text.x = element_blank()) +

theme(legend.title=element_blank()) +

facet_grid(agecrn ~ germbornrn)

f3

f_cobi_age <- f3 + stat_central_tendency(type = "median")

f_cobi_age

f3a <- dfs_analysis %>% ggplot(aes(x = genderscore, linetype = sexr)) +

geom_density() +

ylab("Density") + xlab("Gendered social practices") +

theme_classic() +

theme(axis.text.x = element_blank()) +

theme(legend.title=element_blank()) +

facet_grid(parent_allrn ~ germbornrn)

f3a

f_cobi_parent <- f3a + stat_central_tendency(type = "median")

f_cobi_parent

f3b <- dfs_analysis %>% drop_na(livwpartn) %>%

ggplot(aes(x = genderscore, linetype = sexr)) +

geom_density() +

ylab("Density") + xlab("Gendered social practices") +

theme_classic() +

theme(axis.text.x = element_blank()) +

theme(legend.title=element_blank()) +

facet_grid(livwpartn ~ germbornrn)

f3b

f_cobi_partner <- f3b + stat_central_tendency(type = "median")

f_cobi_partner

#country of origin

frq(dfs_analysis$corigin_big6n)

#for the 6 most frequent countries of origin corigin_big6

f_corigin <- dfs_analysis %>% drop_na(corigin_big6) %>%

ggplot(aes(x = genderscore, y= corigin_big6n, linetype = sexr)) +

geom_density_ridges(quantile_lines = TRUE, quantiles = 0.5, scale = 1, alpha = 0.01) +

ggtitle(label = "Country of birth") +

ylab("Density") + xlab("Gendered social practices") +

theme_classic() +

theme(axis.text.x = element_blank()) +

theme(legend.title=element_blank())

f_corigin

#by region of origin regioc

f_rorigin <- dfs_analysis %>% drop_na(regioncn) %>%

ggplot(aes(x = genderscore, y= regioncn, linetype = sexr)) +

geom_density_ridges(quantile_lines = TRUE, quantiles = 0.5, scale = 1, alpha = 0.01) +

ggtitle(label = "Region of origin") +

ylab("Density") + xlab("Gendered social practices") +

theme_classic() +

theme(axis.text.x = element_blank()) +

theme(legend.title=element_blank())

f_rorigin

#year of immigration

frq(dfs_analysis$imyear10)

f_imy <- dfs_analysis %>% drop_na(imyear10) %>%

ggplot(aes(x = genderscore, y= imyear10, linetype = sexr)) +

geom_density_ridges(quantile_lines = TRUE, quantiles = 0.5, scale = 1, alpha = 0.01) +

ggtitle(label = "Year of immigration") +

ylab("Density") + xlab("Gendered social practices") +

theme_classic() +

theme(axis.text.x = element_blank()) +

theme(legend.title=element_blank())

f_imy

#immigration group

f_imgr <- dfs_analysis %>% drop_na(imgrprn) %>%

ggplot(aes(x = genderscore, y= imgrprn, linetype = sexr)) +

geom_density_ridges(quantile_lines = TRUE, quantiles = 0.5, scale = 1, alpha = 0.01) +

ggtitle(label = "Immigration group") +

ylab("Density") + xlab("Gendered social practices") +

theme_classic() +

theme(axis.text.x = element_blank()) +

theme(legend.title=element_blank())

f_imgr

#resident status

frq(dfs_analysis$resistatrn)

f_restat <- dfs_analysis %>% drop_na(resistatrn) %>%

ggplot(aes(x = genderscore, y= resistatrn, linetype = sexr)) +

geom_density_ridges(quantile_lines = TRUE, quantiles = 0.5, scale = 1, alpha = 0.01) +

ggtitle(label = "Resident status") +

ylab("Density") + xlab("Gendered social practices") +

theme_classic() +

theme(axis.text.x = element_blank()) +

theme(legend.title=element_blank())

f_restat

#other variables

#age

f_age <- dfs_analysis %>% ggplot(aes(x = genderscore, y= agecrn, linetype = sexr)) +

geom_density_ridges(quantile_lines = TRUE, quantiles = 0.5, scale = 1, alpha = 0.01) +

ggtitle(label = "Age") +

ylab("Density") + xlab("Gendered social practices") +

theme_classic() +

theme(axis.text.x = element_blank()) +

theme(legend.title=element_blank())

f_age

#income

f_inc <- dfs_analysis %>% ggplot(aes(x = genderscore, y= incrn, linetype = sexr)) +

geom_density_ridges(quantile_lines = TRUE, quantiles = 0.5, scale = 1, alpha = 0.01) +

ggtitle(label = "Income") +

ylab("Density") + xlab("Gendered social practices") +

theme_classic() +

theme(axis.text.x = element_blank()) +

theme(legend.title=element_blank())

f_inc

#cross classifications

f13 <- dfs_analysis %>%

ggplot(aes(x = genderscore, linetype = sexr)) +

geom_density() +

ylab("Density") + xlab("Gendered social practices") +

theme_classic() +

theme(axis.text.x = element_blank()) +

theme(legend.title=element_blank()) +

facet_grid(agecrn ~ incrn)

f13

f_inc_age <- f13 + stat_central_tendency(type = "median")

f_inc_age

#education

f_edu <- dfs_analysis %>% drop_na(edurn) %>%

ggplot(aes(x = genderscore, y= edurn, linetype = sexr)) +

geom_density_ridges(quantile_lines = TRUE, quantiles = 0.5, scale = 1, alpha = 0.01) +

ggtitle(label = "Formal education") +

ylab("Density") + xlab("Gendered social practices") +

theme_classic() +

theme(axis.text.x = element_blank()) +

theme(legend.title=element_blank())

f_edu

#state of residence

f_eastwest <- dfs_analysis %>% ggplot(aes(x = genderscore, y= eastrn, linetype = sexr)) +

geom_density_ridges(quantile_lines = TRUE, quantiles = 0.5, scale = 1, alpha = 0.01) +

ggtitle(label = "Region of residence in Germany") +

ylab("Density") + xlab("Gendered social practices") +

theme_classic() +

theme(axis.text.x = element_blank()) +

theme(legend.title=element_blank())

f_eastwest

#cross classification

f16 <- dfs_analysis %>%

ggplot(aes(x = genderscore, linetype = sexr)) +

geom_density() +

ylab("Density") + xlab("Gendered social practices") +

theme_classic() +

theme(axis.text.x = element_blank()) +

theme(legend.title=element_blank()) +

facet_grid(agecrn ~ eastrn)

f16

f_age_east <- f16 + stat_central_tendency(type = "median")

f_age_east

#currently living in a same sex/gender partnership

install.packages("ggridges")

library(ggridges)

f_sexor <- dfs_analysis %>% ggplot(aes(x = genderscore, y= sexorn, linetype = sexr)) +

geom_density_ridges(quantile_lines = TRUE, quantiles = 0.5, scale = 1, alpha = 0.01) +

ggtitle(label = "Usually living in a same \nsex/gender partnership") +

ylab("Density") + xlab("Gendered social practices") +

theme_classic() +

theme(axis.text.x = element_blank()) +

theme(legend.title=element_blank())

f_sexor

# parenthood

f_parent <- dfs_analysis %>% ggplot(aes(x = genderscore, y= parent_allrn, linetype = sexr)) +

geom_density_ridges(quantile_lines = TRUE, quantiles = 0.5, scale = 1, alpha = 0.01) +

ggtitle(label = "Parenthood") +

ylab("Density") + xlab("Gendered social practices") +

theme_classic() +

theme(axis.text.x = element_blank()) +

theme(legend.title=element_blank())

f_parent

#cross classification

f19 <- dfs_analysis %>%

ggplot(aes(x = genderscore, linetype = sexr)) +

geom_density() +

ylab("Density") + xlab("Gendered social practices") +

theme_classic() +

theme(axis.text.x = element_blank()) +

theme(legend.title=element_blank()) +

facet_grid(agecrn ~ parent_allrn)

f19

f_age_parent <- f19 + stat_central_tendency(type = "median")

f_age_parent

#living with a partner

frq(dfs_analysis$livwpartn)

f_partner <- dfs_analysis %>% drop_na(livwpartn) %>% ggplot(aes(x = genderscore, y= livwpartn, linetype = sexr)) +

geom_density_ridges(quantile_lines = TRUE, quantiles = 0.5, scale = 1, alpha = 0.01) +

ggtitle(label = "Living with a partner") +

ylab("Density") + xlab("Gendered social practices") +

theme_classic() +

theme(axis.text.x = element_blank()) +

theme(legend.title=element_blank())

f_partner

f21 <- dfs_analysis %>% drop_na(livwpart) %>%

ggplot(aes(x = genderscore, linetype = sexr)) +

geom_density() +

ylab("Density") + xlab("Gendered social practices") +

theme_classic() +

theme(axis.text.x = element_blank()) +

theme(legend.title=element_blank()) +

facet_grid(agecrn ~ livwpartn)

f21

f_age_partner <- f21 + stat_central_tendency(type = "median")

f_age_partner

frq(dfs_analysis$incrn)

f_age_parent

# Print plots to a pdf file

f_sexor

f_age

pdf("Figures_1.pdf", width = 10, height = 8)

print(f_basic)

dev.off()

install.packages("cowplot")

library(cowplot)

plot_grid(f_cobi, f_imy, ncol = 1, align="v")

pdf("Figures_2.pdf", width = 10, height = 5)

print(f_cobi)

print(f_imy)

print(f_restat)

print(f_eastwest)

print(f_partner)

dev.off()

pdf("New figures_3.pdf", width = 15, height = 5)

print(f4)

print(f11)

print(f12)

print(f14)

print(f17)

print(f18a)

dev.off()

pdf("New figures_4a.pdf", width = 20, height = 5)

print(f9)

dev.off()

pdf("New figures_4b.pdf", width = 20, height = 5)

print(f11)

dev.off()

pdf("New figures_4qua.pdf", width = 10, height = 10)

print(f9a)

print(f11a)

dev.off()

pdf("New figures_56.pdf", width = 15, height = 10)

print(f6)

print(f7)

dev.off()

#Cross-classifications

pdf("crosstables_2_4.pdf", width = 8, height = 6)

print(f_cobi_age)

print(f_age_east)

print(f_age_partner)

dev.off()

pdf("crosstables_3_4.pdf", width = 8, height = 6)

print(f_inc_age)

print(f_age_parent)

dev.off()

pdf("crosstables_2_2.pdf", width = 10, height = 10)

print(f_cobi_partner)

dev.off()

pdf("crosstables_2_3.pdf", width = 15, height = 10)

print(f_cobi_parent)

dev.off()

#-----------------------------------------------------------------------------------#

#SENSITIVITY ANALYSIS

#frq for stratifying variables

dffrequency <- c("sexr", "agecr", "sexor",

"parentr", "livwpart",

"migbackr", "germbornr", "regionc")

dffreq1 <- df_analysis[dffrequency]

frq(dffreq1)

#stratified regression models to define composite gender score

#stratifiers = variables that showed different patterns in gendered social practices

#migration status: germbron, migration background, region

frq(df_analysis$germbornr)

dfmig <- subset(df_analysis, germbornr == "not born in Germany")

dfnmig <- subset(df_analysis, germbornr == "born in Germany or immigr.<1950")

frq(df_analysis$migbackr)

dfmgn <- subset(df_analysis, migbackr == "no migration background")

dfmgd <- subset(df_analysis, migbackr == "direct migration background")

dfmgi <- subset(df_analysis, migbackr == "indirect migration background")

frq(df_analysis$regionc)

dfger <- subset(df_analysis, regionc == "Germany")

dfeast <- subset(df_analysis, regionc == "Eastern Europe")

dfwest <- subset(df_analysis, regionc == "Western Europe")

dfswasia <- subset(df_analysis, regionc == "Central Asia")

dfmideast <- subset(df_analysis, regionc == "Middle East")

#sociodemographics: age, having children, living with partner, sexual orientatation

frq(df_analysis$agecr)

age1 <- subset(df_analysis, agecr == "18-30 years")

age2 <- subset(df_analysis, agecr == "31-45 years")

age3 <- subset(df_analysis, agecr == "46-60 years")

age4 <- subset(df_analysis, agecr == "61-75+ years")

frq(df_analysis$livwpart)

lp <- subset(df_analysis, livwpart == "living with a partner")

nlp <- subset(df_analysis, livwpart == "single or not living with their partner")

frq(df_analysis$parentr)

dfpar <- subset(df_analysis, parentr == "parent")

dfnpar <- subset(df_analysis, parentr == "childless")

glimpse(df_analysis)

frq(df_analysis$sexor)

sa_osex <- subset(df_analysis, sexor == "probably heterosexual")

sa_ssex <- subset(df_analysis, sexor == "probably bi/homosexual")

sa_missi <- subset(df_analysis, sexor == "insufficient information")

#CONTENT BASED MODEL (selected for gender score)

#base model to build for each subgroup

mc3 <- glmer(sexr ~ bip_197_01 + bip_197_02 + bip_197_04 +

bip_197_06 + bip_197_07 + bip_197_08 +

pgexppt + plb0018 + plh0033 + plh0036 + plh0039 +

plh0040 + plh0174 + plh0176 +

plh0178 +

plh0204_h + plh0335 + pli0043_h + pli0046 + pli0049_h +

pli0051 + (1 | cid),

data = gvr,

family = binomial(link='logit'),

control = glmerControl(optimizer = "bobyqa"),

nAGQ = 0)

summary(mc3)

#MIGRANTS VS NO MIGRANTS

#not germborn

sngermborn <- glmer(sexr ~ bip_197_01 + bip_197_02 + bip_197_04 +

bip_197_06 + bip_197_07 + bip_197_08 +

pgexppt + plb0018 + plh0033 + plh0036 + plh0039 +

plh0040 + plh0174 + plh0176 +

plh0178 +

plh0204_h + plh0335 + pli0043_h + pli0046 + pli0049_h +

pli0051 + (1 | cid),

data = dfmig,

family = binomial(link='logit'),

control = glmerControl(optimizer = "bobyqa"),

nAGQ = 0)

summary(sngermborn)

#stepwise exclusion

#plh0176

#plh0174

#bip_197_02

#plb0018

#plh0033

#plh0335

#below 0.1

#bip_197_06

#plh0040

#pli0046

#bip_197_08

#plh0036

sngermborn1 <- glmer(sexr ~ bip_197_01 + bip_197_04 +

bip_197_07 + bip_197_08 + bip_197_06 +

pgexppt + plh0039 + plh0036 +

plh0178 + pli0046 + plh0040 +

plh0204_h + pli0043_h + pli0049_h +

pli0051 + (1 | cid),

data = dfmig,

family = binomial(link='logit'),

control = glmerControl(optimizer = "bobyqa"),

nAGQ = 0)

summary(sngermborn1)

#germborn

sgermborn <- glmer(sexr ~ bip_197_01 + bip_197_02 + bip_197_04 +

bip_197_06 + bip_197_07 + bip_197_08 +

pgexppt + plb0018 + plh0033 + plh0036 + plh0039 +

plh0040 + plh0174 + plh0176 +

plh0178 +

plh0204_h + plh0335 + pli0043_h + pli0046 + pli0049_h +

pli0051 + (1 | cid),

data = dfnmig,

family = binomial(link='logit'),

control = glmerControl(optimizer = "bobyqa"),

nAGQ = 0)

summary(sgermborn)

#stepwise exclusion

#pli0046

#plh0036

#plh0176

#bip_197_06

#plh0033

#bip_197_02

sgermborn1 <- glmer(sexr ~ bip_197_01 + bip_197_02 + bip_197_04 +

bip_197_07 + bip_197_08 +

pgexppt + plb0018 + plh0039 +

plh0040 + plh0174 +

plh0178 +

plh0204_h + plh0335 + pli0043_h + pli0049_h +

pli0051 + (1 | cid),

data = dfnmig,

family = binomial(link='logit'),

control = glmerControl(optimizer = "bobyqa"),

nAGQ = 0)

summary(sgermborn1)

summary(sgermborn1)

summary(sngermborn1)

#MIGRATION BACKGROUND VS. NO OR INDIRECT MIGBACK

#no migback

snmb <- glmer(sexr ~ bip_197_01 + bip_197_02 + bip_197_04 +

bip_197_06 + bip_197_07 + bip_197_08 +

pgexppt + plb0018 + plh0033 + plh0036 + plh0039 +

plh0040 + plh0174 + plh0176 +

plh0178 +

plh0204_h + plh0335 + pli0043_h + pli0046 + pli0049_h +

pli0051 + (1 | cid),

data = dfmgn,

family = binomial(link='logit'),

control = glmerControl(optimizer = "bobyqa"),

nAGQ = 0)

summary(snmb)

#stepwise exclusion

#plh0033

#plh0036

#pli0046

#plh0176

#bip_197_06

#bip_197_02

#plb0018

#plh0335

snmb1 <- glmer(sexr ~ bip_197_01 + bip_197_02 + bip_197_04 +

bip_197_07 + bip_197_08 +

pgexppt + plb0018 + plh0039 +

plh0040 + plh0174 +

plh0178 + plh0335 +

plh0204_h + pli0043_h + pli0049_h +

pli0051 + (1 | cid),

data = dfmgn,

family = binomial(link='logit'),

control = glmerControl(optimizer = "bobyqa"),

nAGQ = 0)

summary(snmb1)

#direct migback

sdmb <- glmer(sexr ~ bip_197_01 + bip_197_02 + bip_197_04 +

bip_197_06 + bip_197_07 + bip_197_08 +

pgexppt + plb0018 + plh0033 + plh0036 + plh0039 +

plh0040 + plh0174 + plh0176 +

plh0178 +

plh0204_h + plh0335 + pli0043_h + pli0046 + pli0049_h +

pli0051 + (1 | cid),

data = dfmgd,

family = binomial(link='logit'),

control = glmerControl(optimizer = "bobyqa"),

nAGQ = 0)

summary(sdmb)

#stepwise exclusion

#plh0176

#bip_197_02

#plh0174

#plb0018

#plh0033

#plh0335

#bip_197_06

#plh0040

#pli0046

#bip_197_08

#plh0036

sdmb1 <- glmer(sexr ~ bip_197_01 + bip_197_04 + bip_197_06 +

bip_197_07 + bip_197_08 +

pgexppt + plh0036 + plh0039 + plh0040 + pli0046 +

plh0178 +

plh0204_h + pli0043_h + pli0049_h +

pli0051 + (1 | cid),

data = dfmgd,

family = binomial(link='logit'),

control = glmerControl(optimizer = "bobyqa"),

nAGQ = 0)

summary(sdmb1)

#indirect migback

simb <- glmer(sexr ~ bip_197_01 + bip_197_02 + bip_197_04 +

bip_197_06 + bip_197_07 + bip_197_08 +

pgexppt + plb0018 + plh0033 + plh0036 + plh0039 +

plh0040 + plh0174 + plh0176 +

plh0178 +

plh0204_h + plh0335 + pli0043_h + pli0046 + pli0049_h +

pli0051 + (1 | cid),

data = dfmgi,

family = binomial(link='logit'),

control = glmerControl(optimizer = "bobyqa"),

nAGQ = 0)

summary(simb)

#stepwise exclusion

#plh0335

#plh0040

#bip_197_02

#plh0178

#plh0036

#bip_197_01

#bip_197_04

#bip_197_06

#pli0046

#plh0033

#plh0176

#bip_197_07

simb1 <- glmer(sexr ~ bip_197_07 + bip_197_08 +

pgexppt + plb0018 + plh0039 +

plh0174 + plh0176 +

plh0204_h + pli0043_h + pli0049_h +

pli0051 + (1 | cid),

data = dfmgi,

family = binomial(link='logit'),

control = glmerControl(optimizer = "bobyqa"),

nAGQ = 0)

summary(simb1)

summary(snmb1)

summary(sdmb1)

summary(simb1)

#corigin = region

#germany

#see above germborn

#eastern europe

seast <- glmer(sexr ~ bip_197_01 + bip_197_02 + bip_197_04 +

bip_197_06 + bip_197_07 + bip_197_08 +

pgexppt + plb0018 + plh0033 + plh0036 + plh0039 +

plh0040 + plh0174 + plh0176 +

plh0178 +

plh0204_h + plh0335 + pli0043_h + pli0046 + pli0049_h +

pli0051 + (1 | cid),

data = dfeast,

family = binomial(link='logit'),

control = glmerControl(optimizer = "bobyqa"),

nAGQ = 0)

summary(seast)

#stepwise exclusion

#plh0040

#plh0176

#plh0033

#bip_197_02

#plh0174

#plb0018

#pli0051

#plh0335

#plh0036

#bip_197_06

#below 0.1

#save model with all p-values below 0.05

seast1a <- glmer(sexr ~ bip_197_01 + bip_197_04 +

bip_197_07 + bip_197_08 +

pgexppt +

plh0204_h + pli0043_h + pli0049_h +

bip_197_04 + plh0178 + bip_197_08 + plh0039 + pli0046 +

(1 | cid),

data = dfeast,

family = binomial(link='logit'),

control = glmerControl(optimizer = "bobyqa"),

nAGQ = 0)

summary(seast1a)

#bip_197_04

#plh0178

#bip_197_08

#plh0039

#pli0046

seast1 <- glmer(sexr ~ bip_197_01 +

bip_197_07 +

pgexppt +

plh0204_h + pli0043_h + pli0049_h +

(1 | cid),

data = dfeast,

family = binomial(link='logit'),

control = glmerControl(optimizer = "bobyqa"),

nAGQ = 0)

summary(seast1)

#western europe

swest <- glmer(sexr ~ bip_197_01 + bip_197_02 + bip_197_04 +

bip_197_06 + bip_197_07 + bip_197_08 +

pgexppt + plb0018 + plh0033 + plh0036 + plh0039 +

plh0040 + plh0174 + plh0176 +

plh0178 +

plh0204_h + plh0335 + pli0043_h + pli0046 + pli0049_h +

pli0051 + (1 | cid),

data = dfwest,

family = binomial(link='logit'),

control = glmerControl(optimizer = "bobyqa"),

nAGQ = 0)

summary(swest)

#stepwise exclusion

#plh0036

#plh0178

#bip_197_08

#plb0018

#plh0174

#bip_197_02

#plh0040

#bip_197_06

#plh0176

#bip_197_01

#plh0033

#pli0046

#plh0039

#pli0051

swest1 <- glmer(sexr ~ bip_197_04 + bip_197_07 +

pgexppt +

plh0204_h + plh0335 + pli0043_h + pli0049_h +

(1 | cid),

data = dfwest,

family = binomial(link='logit'),

control = glmerControl(optimizer = "bobyqa"),

nAGQ = 0)

summary(swest1)

#only below 0.01

#bip_197_04

#plh0335

swest1a <- glmer(sexr ~ + bip_197_07 +

pgexppt +

plh0204_h + pli0043_h + pli0049_h +

(1 | cid),

data = dfwest,

family = binomial(link='logit'),

control = glmerControl(optimizer = "bobyqa"),

nAGQ = 0)

summary(swest1a)

#South west asia

sasia <- glmer(sexr ~ bip_197_01 + bip_197_02 + bip_197_04 +

bip_197_06 + bip_197_07 + bip_197_08 +

pgexppt + plb0018 + plh0033 + plh0036 + plh0039 +

plh0040 + plh0174 + plh0176 +

plh0178 +

plh0204_h + plh0335 + pli0043_h + pli0046 + pli0049_h +

pli0051 + (1 | cid),

data = dfswasia,

family = binomial(link='logit'),

control = glmerControl(optimizer = "bobyqa"),

nAGQ = 0)

summary(sasia)

#stepwise exclusion

#pli0051

#bip_197_06

#plb0018

#plh0036

#plh0039

#plh0335

#bip_197_07

#plh0040

#pli0046

#plh0178

#bip_197_04

#bip_197_08

#plh0033

#bip_197_01

sasia1 <- glmer(sexr ~ bip_197_02 +

pgexppt +

plh0174 + plh0176 +

plh0204_h + pli0043_h + pli0049_h +

(1 | cid),

data = dfswasia,

family = binomial(link='logit'),

control = glmerControl(optimizer = "bobyqa"),

nAGQ = 0)

summary(sasia1)

#middle east

smideast <- glmer(sexr ~ bip_197_01 + bip_197_02 + bip_197_04 +

bip_197_06 + bip_197_07 + bip_197_08 +

pgexppt + plb0018 + plh0033 + plh0036 + plh0039 +

plh0040 + plh0174 + plh0176 +

plh0178 +

plh0204_h + plh0335 + pli0043_h + pli0046 + pli0049_h +

pli0051 + (1 | cid),

data = dfmideast,

family = binomial(link='logit'),

control = glmerControl(optimizer = "bobyqa"),

nAGQ = 0)

summary(smideast)

#stepwise exclusion

#pli0046

#bip_197_04

#bip_197_02

#pli0051

#bip_197_06

#plh0176

#bip_197_07

#plh0040

#plh0039

#plb0018

#plh0174

#plh0178

#plh0335

#plh0033

#plh0204_h

#bip_197_01

smideast1 <- glmer(sexr ~ bip_197_08 +

pgexppt + plh0036 +

pli0043_h + pli0049_h +

(1 | cid),

data = dfmideast,

family = binomial(link='logit'),

control = glmerControl(optimizer = "bobyqa"),

nAGQ = 0)

summary(smideast1)

summary(seast1)

summary(swest1)

summary(sasia1)

summary(smideast1)

#AGE

#age1

sage1 <- glmer(sexr ~ bip_197_01 + bip_197_02 + bip_197_04 +

bip_197_06 + bip_197_07 + bip_197_08 +

pgexppt + plb0018 + plh0033 + plh0036 + plh0039 +

plh0040 + plh0174 + plh0176 +

plh0178 +

plh0204_h + plh0335 + pli0043_h + pli0046 + pli0049_h +

pli0051 + (1 | cid),

data = age1,

family = binomial(link='logit'),

control = glmerControl(optimizer = "bobyqa"),

nAGQ = 0)

summary(sage1)

#stepwise exclusion

#plh0040

#bip_197_06

#bip_197_02

#plh0335

#plh0036

#plh0033

#plh0176

#plh0174

#pli0046

#plh0178

sage11 <- glmer(sexr ~ bip_197_01 + bip_197_04 +

bip_197_07 + bip_197_08 +

pgexppt + plb0018 + plh0039 +

plh0204_h + pli0043_h + pli0049_h +

pli0051 + (1 | cid),

data = age1,

family = binomial(link='logit'),

control = glmerControl(optimizer = "bobyqa"),

nAGQ = 0)

summary(sage11)

#age2

sage2 <- glmer(sexr ~ bip_197_01 + bip_197_02 + bip_197_04 +

bip_197_06 + bip_197_07 + bip_197_08 +

pgexppt + plb0018 + plh0033 + plh0036 + plh0039 +

plh0040 + plh0174 + plh0176 +

plh0178 +

plh0204_h + plh0335 + pli0043_h + pli0046 + pli0049_h +

pli0051 + (1 | cid),

data = age2,

family = binomial(link='logit'),

control = glmerControl(optimizer = "bobyqa"),

nAGQ = 0)

summary(sage2)

#stepwise exclusion

#bip_197_02

#plh0036

#plh0335

#bip_197_06

#plh0176

#plh0033

#pli0046

#plh0178

#bip_197_01

sage21 <- glmer(sexr ~ bip_197_01 + bip_197_04 +

bip_197_07 + bip_197_08 +

pgexppt + plb0018 + plh0039 +

plh0040 + plh0174 + plh0178 +

plh0204_h + pli0043_h + pli0049_h +

pli0051 + (1 | cid),

data = age2,

family = binomial(link='logit'),

control = glmerControl(optimizer = "bobyqa"),

nAGQ = 0)

summary(sage21)

#age3

sage3 <- glmer(sexr ~ bip_197_01 + bip_197_02 + bip_197_04 +

bip_197_06 + bip_197_07 + bip_197_08 +

pgexppt + plb0018 + plh0033 + plh0036 + plh0039 +

plh0040 + plh0174 + plh0176 +

plh0178 +

plh0204_h + plh0335 + pli0043_h + pli0046 + pli0049_h +

pli0051 + (1 | cid),

data = age3,

family = binomial(link='logit'),

control = glmerControl(optimizer = "bobyqa"),

nAGQ = 0)

summary(sage3)

#stepwise exclusion

#plh0178

#plh0335

#bip_197_02

#plh0036

#plb0018

#plh0033

#pli0046

#plh0040

#bip_197_06

#plh0176

sage31 <- glmer(sexr ~ bip_197_01 + bip_197_04 + bip_197_06 +

bip_197_07 + bip_197_08 +

pgexppt + plh0039 + plh0040 + pli0046 +

plh0174 + plh0176 +

plh0204_h + pli0043_h + pli0049_h +

pli0051 + (1 | cid),

data = age3,

family = binomial(link='logit'),

control = glmerControl(optimizer = "bobyqa"),

nAGQ = 0)

summary(sage31)

#age4

sage4 <- glmer(sexr ~ bip_197_01 + bip_197_02 + bip_197_04 +

bip_197_06 + bip_197_07 + bip_197_08 +

pgexppt + plb0018 + plh0033 + plh0036 + plh0039 +

plh0040 + plh0174 + plh0176 +

plh0178 +

plh0204_h + plh0335 + pli0043_h + pli0046 + pli0049_h +

pli0051 + (1 | cid),

data = age4,

family = binomial(link='logit'),

control = glmerControl(optimizer = "bobyqa"),

nAGQ = 0)

summary(sage4)

#stepwise exclusion

#plh0176

#plh0039

#plh0036

#pli0051

#bip_197_02

#bip_197_06

#bip_197_08

#plb0018

#plh0033

#plh0335

#plh0178

#plh0174

#bip_197_01

sage41 <- glmer(sexr ~ bip_197_01 + bip_197_04 +

bip_197_07 +

pgexppt +

plh0040 +

plh0204_h + pli0043_h + pli0046 + pli0049_h +

(1 | cid),

data = age4,

family = binomial(link='logit'),

control = glmerControl(optimizer = "bobyqa"),

nAGQ = 0)

summary(sage41)

summary(sage11)

summary(sage21)

summary(sage31)

summary(sage41)

#COHABITATION STATUS

#living with partner

slp <- glmer(sexr ~ bip_197_01 + bip_197_02 + bip_197_04 +

bip_197_06 + bip_197_07 + bip_197_08 +

pgexppt + plb0018 + plh0033 + plh0036 + plh0039 +

plh0040 + plh0174 + plh0176 +

plh0178 +

plh0204_h + plh0335 + pli0043_h + pli0046 + pli0049_h +

pli0051 + (1 | cid),

data = lp,

family = binomial(link='logit'),

control = glmerControl(optimizer = "bobyqa"),

nAGQ = 0)

summary(slp)

#stepwise exclusion

#bip_197_06

#plh0176

#bip_197_02

#plh0178

#plb0018

#plh0033

#plh0036

#pli0046

slp1 <- glmer(sexr ~ bip_197_01 + bip_197_04 +

bip_197_07 + bip_197_08 +

pgexppt + plh0039 + pli0046 +

plh0040 + plh0174 +

plh0204_h + plh0335 + pli0043_h + pli0049_h +

pli0051 + (1 | cid),

data = lp,

family = binomial(link='logit'),

control = glmerControl(optimizer = "bobyqa"),

nAGQ = 0)

summary(slp1)

#not living with partner

snlp <- glmer(sexr ~ bip_197_01 + bip_197_02 + bip_197_04 +

bip_197_06 + bip_197_07 + bip_197_08 +

pgexppt + plb0018 + plh0033 + plh0036 + plh0039 +

plh0040 + plh0174 + plh0176 +

plh0178 +

plh0204_h + plh0335 + pli0043_h + pli0046 + pli0049_h +

pli0051 + (1 | cid),

data = nlp,

family = binomial(link='logit'),

control = glmerControl(optimizer = "bobyqa"),

nAGQ = 0)

summary(snlp)

#stepwise exclusion

#plh0335

#plh0178

#bip_197_02

#bip_197_01

#plh0036

#bip_197_06

#pli0046

#plh0040

#plh0174

#plh0176

#plh0033

snlp1 <- glmer(sexr ~ bip_197_04 +

bip_197_07 + bip_197_08 +

pgexppt + plb0018 + plh0039 + plh0033 +

plh0204_h + pli0043_h + pli0049_h + plh0176 + plh0174 +

pli0051 + (1 | cid),

data = nlp,

family = binomial(link='logit'),

control = glmerControl(optimizer = "bobyqa"),

nAGQ = 0)

summary(snlp1)

summary(slp1)

summary(snlp1)

#PARENTHOOD

#parent

sparent <- glmer(sexr ~ bip_197_01 + bip_197_02 + bip_197_04 +

bip_197_06 + bip_197_07 + bip_197_08 +

pgexppt + plb0018 + plh0033 + plh0036 + plh0039 +

plh0040 + plh0174 + plh0176 +

plh0178 +

plh0204_h + plh0335 + pli0043_h + pli0046 + pli0049_h +

pli0051 + (1 | cid),

data = dfpar,

family = binomial(link='logit'),

control = glmerControl(optimizer = "bobyqa"),

nAGQ = 0)

summary(sparent)

#stepwise exclusion

#plh0178

#plh0176

#bip_197_02

#pli0046

#plh0033

#bip_197_06

#plh0036

sparent1 <- glmer(sexr ~ bip_197_01 + bip_197_04 +

bip_197_07 + bip_197_08 +

pgexppt + plb0018 + plh0039 +

plh0040 + plh0174 +

plh0204_h + plh0335 + pli0043_h + pli0049_h +

pli0051 + (1 | cid),

data = dfpar,

family = binomial(link='logit'),

control = glmerControl(optimizer = "bobyqa"),

nAGQ = 0)

summary(sparent1)

#not parent

snparent <- glmer(sexr ~ bip_197_01 + bip_197_02 + bip_197_04 +

bip_197_06 + bip_197_07 + bip_197_08 +

pgexppt + plb0018 + plh0033 + plh0036 + plh0039 +

plh0040 + plh0174 + plh0176 +

plh0178 +

plh0204_h + plh0335 + pli0043_h + pli0046 + pli0049_h +

pli0051 + (1 | cid),

data = dfnpar,

family = binomial(link='logit'),

control = glmerControl(optimizer = "bobyqa"),

nAGQ = 0)

summary(snparent)

#stepwise exclusion

#plh0335

#bip_197_06

#plb0018

#plh0033

#plh0178

#bip_197_02

#plh0040

#plh0036

#plh0176

#bip_197_01

#plh0174

snparent1 <- glmer(sexr ~ bip_197_04 +

bip_197_07 + bip_197_08 +

pgexppt + plh0039 + plh0174 +

plh0204_h + pli0043_h + pli0046 + pli0049_h +

pli0051 + (1 | cid),

data = dfnpar,

family = binomial(link='logit'),

control = glmerControl(optimizer = "bobyqa"),

nAGQ = 0)

summary(snparent1)

summary(sparent1)

summary(snparent1)

#SEXUAL ATTRACTION

#probably heterosexual

sosex <- glmer(sexr ~ bip_197_01 + bip_197_02 + bip_197_04 +

bip_197_06 + bip_197_07 + bip_197_08 +

pgexppt + plb0018 + plh0033 + plh0036 + plh0039 +

plh0040 + plh0174 + plh0176 +

plh0178 +

plh0204_h + plh0335 + pli0043_h + pli0046 + pli0049_h +

pli0051 + (1 | cid),

data = sa_osex,

family = binomial(link='logit'),

control = glmerControl(optimizer = "bobyqa"),

nAGQ = 0)

summary(sosex)

#stepwise exclusion

#bip_197_06

#plh0176

#plb0018

#plh0033

#bip_197_02

#pli0046

#plh0036

#plh0178

sosex1 <- glmer(sexr ~ bip_197_01 + bip_197_04 +

bip_197_07 + bip_197_08 +

pgexppt + plh0039 +

plh0040 + plh0174 +

plh0204_h + plh0335 + pli0043_h + pli0049_h +

pli0051 + (1 | cid),

data = sa_osex,

family = binomial(link='logit'),

control = glmerControl(optimizer = "bobyqa"),

nAGQ = 0)

summary(sosex1)

#probably bi/homosexual

sssex <- glmer(sexr ~ bip_197_01 + bip_197_02 + bip_197_04 +

bip_197_06 + bip_197_07 + bip_197_08 +

pgexppt + plb0018 + plh0033 + plh0036 + plh0039 +

plh0040 + plh0174 + plh0176 +

plh0178 +

plh0204_h + plh0335 + pli0043_h + pli0046 + pli0049_h +

pli0051 + (1 | cid),

data = sa_ssex,

family = binomial(link='logit'),

control = glmerControl(optimizer = "bobyqa"),

nAGQ = 0)

summary(sssex)

#stepwise exclusion

#plh0335

#plh0204_h

#plh0040

#bip_197_02

#plh0033

#plh0176

#plh0178

#bip_197_07

#bip_197_08

#pli0046

#plh0036

#plh0174

#pgexppt

#plb0018

#bip_197_01

#bip_197_06

sssex1 <- glmer(sexr ~ bip_197_04 +

plh0039 +

pli0043_h + pli0049_h +

pli0051 + (1 | cid),

data = sa_ssex,

family = binomial(link='logit'),

control = glmerControl(optimizer = "bobyqa"),

nAGQ = 0)

summary(sssex1)

#not sufficient info

ssexi <- glmer(sexr ~ bip_197_01 + bip_197_02 + bip_197_04 +

bip_197_06 + bip_197_07 + bip_197_08 +

pgexppt + plb0018 + plh0033 + plh0036 + plh0039 +

plh0040 + plh0174 + plh0176 +

plh0178 +

plh0204_h + plh0335 + pli0043_h + pli0046 + pli0049_h +

pli0051 + (1 | cid),

data = sa_missi,

family = binomial(link='logit'),

control = glmerControl(optimizer = "bobyqa"),

nAGQ = 0)

summary(ssexi)

#stepwise exclusion

#plh0033

#pli0046

#bip_197_06

#plh0040

#plh0036

#bip_197_02

#plh0176

#plh0174

#plh0335

#plh0178

#bip_197_01

#plb0018

ssexi1 <- glmer(sexr ~ bip_197_04 +

bip_197_07 + bip_197_08 +

pgexppt + plh0039 + plb0018 +

plh0204_h + pli0043_h + pli0049_h +

pli0051 + (1 | cid),

data = sa_missi,

family = binomial(link='logit'),

control = glmerControl(optimizer = "bobyqa"),

nAGQ = 0)

summary(ssexi1)

summary(sosex1)

summary(sssex1)

summary(ssexi1)

#all regmodels from sensitivity analysis

summary(sgermborn1)

summary(sngermborn1)

summary(snmb1)

summary(sdmb1)

summary(simb1)

summary(seast1)

summary(swest1)

summary(sasia1)

summary(smideast1)

summary(sage11)

summary(sage21)

summary(sage31)

summary(sage41)

summary(slp1)

summary(snlp1)

summary(sparent1)

summary(snparent1)

summary(sosex1)

summary(sssex1)

summary(ssexi1)

#------------------------#

#non-response analysis

#generate dataframe with gender variables only

df_sample_missings <- df_sample2018[!complete.cases(df_sample2018$bip_197_01,

df_sample2018$bip_197_04,

df_sample2018$bip_197_07,

df_sample2018$bip_197_08,

df_sample2018$pgexppt,

df_sample2018$plh0039,

df_sample2018$plh0040,

df_sample2018$plh0174,

df_sample2018$plh0204_h,

df_sample2018$plh0335,

df_sample2018$pli0043_h,

df_sample2018$pli0049_h,

df_sample2018$pli0051), ]

glimpse(df_sample_missings)

#dataset with variables included in the analysis

analysis1 <- c("pid", "sexr", "cid",

"age", "agecr", "edur",

"incr",

"germstate", "eastr", "sexor",

"single", "livwpart",

"migbackr", "migbackbin", "germbornr", "resistatr",

"corigin_big6", "regionc", "continentc",

"immiyear", "imyear5", "imyearcatr", "imgrpr",

"mcs",

"pcs",

"bip_197_01", "bip_197_04",

"bip_197_07", "bip_197_08",

"pgexppt", "plh0039",

"plh0040", "plh0174",

"plh0204_h", "plh0335", "pli0043_h", "pli0049_h",

"pli0051", "biopr", "socpr", "parent_allr")

dfs_nonresp <- df_sample_missings[analysis1]

dfs_nonresp$nonresp <- 1

glimpse(dfs_nonresp)

nr <- c("pid", "cid", "nonresp")

dfs_nonresp1 <- dfs_nonresp[nr]

dfs_analysis$nonresp <- 0

glimpse(dfs_analysis)

nr <- c("pid", "cid", "nonresp")

dfs_analysis_nr <- dfs_analysis[nr]

df_nra <- df_sample2018

df_nra1 <- left_join(df_nra, dfs_nonresp1, by=c("pid", "cid"))

df_nra2 <- left_join(df_nra1, dfs_analysis_nr, by=c("pid", "cid"))

glimpse(df_nra2)

summary(df_nra2$nonresp.x) #non-responder

summary(df_nra2$nonresp.y) #valid cases

df_nra2$MY <- paste(df_nra2$nonresp.x, df_nra2$nonresp.y)

glimpse(df_nra2)

df_nra2 <- mutate(df_nra2, nonresp = ifelse(MY == "NA 0", 0,

ifelse(MY == "1 NA", 1, NA)))

glimpse(df_nra2)

frq(df_nra2$nonresp)

df_nra2 <- mutate(df_nra2, imyear10 = ifelse(immiyear %in% 2009:2018, 1,

ifelse(immiyear %in% 1950:2008, 0, NA)))

table(df_nra2$imyear10, exclude = NULL)

df_nra2$imyear10 <- dplyr::recode_factor(df_nra2$imyear10,

'0' = "before 2009", '1' = "2009 to 2018")

frq(df_nra2$imyear10)

#frequencies for demographics

dffrequency <- c("sexr", "agecr", "edur","incr",

"eastr", "sexor",

"parent_allr", "single", "livwpart",

"migbackr", "migbackbin", "germbornr", "resistatr",

"corigin_big6", "regionc", "continentc",

"imyear5", "imyearcatr", "imgrpr",

"nonresp")

df_frqnr <- dfs_nonresp[dffrequency]

frq(df_frqnr)

#test for statistical differences between sample and non-response

library(gmodels)

library(psych)

library(corrplot)

df_nra2$nonresp <- as.factor(df_nra2$nonresp)

frq(df_nra2$nonresp)

chi <- chisq.test(df_nra2$sexr, df_nra2$nonresp)

chi$observed

chi$expected

corrplot(chi$residuals, is.cor = FALSE)

chi <- chisq.test(df_nra2$agecr, df_nra2$nonresp)

chi$observed

chi$expected

corrplot(chi$residuals, is.cor = FALSE)

chi <- chisq.test(df_nra2$edur, df_nra2$nonresp)

chi$observed

chi$expected

corrplot(chi$residuals, is.cor = FALSE)

chi <- chisq.test(df_nra2$incr, df_nra2$nonresp)

chi$observed

chi$expected

corrplot(chi$residuals, is.cor = FALSE)

chi <- chisq.test(df_nra2$eastr, df_nra2$nonresp)

chi$observed

chi$expected

corrplot(chi$residuals, is.cor = FALSE)

chi <- chisq.test(df_nra2$germbornr, df_nra2$nonresp)

chi$observed

chi$expected

corrplot(chi$residuals, is.cor = FALSE)

chi <- chisq.test(df_nra2$migbackr, df_nra2$nonresp)

chi$observed

chi$expected

corrplot(chi$residuals, is.cor = FALSE)

chi <- chisq.test(df_nra2$corigin_big6, df_nra2$nonresp)

chi$observed

chi$expected

corrplot(chi$residuals, is.cor = FALSE)

chi <- chisq.test(df_nra2$regionc, df_nra2$nonresp)

chi$observed

chi$expected

corrplot(chi$residuals, is.cor = FALSE)

chi <- chisq.test(df_nra2$imyearcatr, df_nra2$nonresp)

chi$observed

chi$expected

corrplot(chi$residuals, is.cor = FALSE)

chi <- chisq.test(df_nra2$imgrpr, df_nra2$nonresp)

chi$observed

chi$expected

corrplot(chi$residuals, is.cor = FALSE)

chi <- chisq.test(df_nra2$resistatr, df_nra2$nonresp)

chi$observed

chi$expected

corrplot(chi$residuals, is.cor = FALSE)

chi <- chisq.test(df_nra2$parentr, df_nra2$nonresp)

chi$observed

chi$expected

corrplot(chi$residuals, is.cor = FALSE)

chi <- chisq.test(df_nra2$livwpart, df_nra2$nonresp)

chi$observed

chi$expected

corrplot(chi$residuals, is.cor = FALSE)

chi <- chisq.test(df_nra2$sexor, df_nra2$nonresp)

chi$observed

chi$expected

corrplot(chi$residuals, is.cor = FALSE)

#----------------------------------------------#

#differentiate between not answered/valid/not applicable and not included in survey wave

load("/Users/lisawandschneider/sciebo2/meinGendEpi - Work in Progress/5_SOEP_composite gender measure/Missing cases Analysis/df_missings_c.Rda")

df_nra3 <- left_join(df_nra2, df_missings_c, by=c("pid", "cid"))

glimpse(df_nra3)

frq(df_nra3$nonresp1)

df_nra3$MY <- paste(df_nra3$nonresp.x, df_nra3$nonresp.y, df_nra3$nonresp1)

glimpse(df_nra3)

frq(df_nra3$MY)

df_nra3 <- mutate(df_nra3, nonrespc = ifelse(MY == "1 NA 2", 1,

ifelse(MY == "NA 0 NA", 0,

ifelse(MY == "1 NA NA", 2, NA))))

frq(df_nra3$nonrespc)

df_nra3 <- mutate(df_nra3, missings_d = ifelse(MY == "1 NA 2", 1,

ifelse(MY == "NA 0 NA", 0, NA)))

frq(df_nra3$missings_d)

#dataset with valid cases or missings, excluding cases where question not part of the survey

df_missings_f <- df_nra3[complete.cases(df_nra3$missings_d), ]

df_valid <- filter(df_nra3, nonrespc == 0)

df_missings <- filter(df_nra3, nonrespc == 1)

df_notinsurvey <- filter(df_nra3, nonrespc == 2)

#frequencies for demographics

dffrequency <- c("sexr", "agecr", "edur","incr",

"eastr", "sexor",

"parent_allr", "single", "livwpart",

"migbackr", "migbackbin", "germbornr", "resistatr",

"corigin_big6", "regionc", "continentc",

"imyear10", "imyearcatr", "imgrpr",

"nonresp", "missings_d")

df_frqnr <- df_missings[dffrequency]

frq(df_frqnr)

#test for statistical differences

library(gmodels)

library(psych)

library(corrplot)

df_missings_f$missings_d <- as.factor(df_missings_f$missings_d)

frq(df_missings_f$missings_d)

chisq.test(df_missings_f$sexr, df_missings_f$missings_d)

chi <- chisq.test(df_missings_f$sexr, df_missings_f$missings_d)

chi$observed

chi$expected

corrplot(chi$residuals, is.cor = FALSE)

chisq.test(df_missings_f$agecr, df_missings_f$missings_d)

chi <- chisq.test(df_missings_f$agecr, df_missings_f$missings_d)

chi$observed

chi$expected

corrplot(chi$residuals, is.cor = FALSE)

chisq.test(df_missings_f$edur, df_missings_f$missings_d)

chi <- chisq.test(df_missings_f$edur, df_missings_f$missings_d)

chi$observed

chi$expected

corrplot(chi$residuals, is.cor = FALSE)

chisq.test(df_missings_f$incr, df_missings_f$missings_d)

chi <- chisq.test(df_missings_f$incr, df_missings_f$missings_d)

chi$observed

chi$expected

corrplot(chi$residuals, is.cor = FALSE)

chisq.test(df_missings_f$eastr, df_missings_f$missings_d)

chi <- chisq.test(df_missings_f$eastr, df_missings_f$missings_d)

chi$observed

chi$expected

corrplot(chi$residuals, is.cor = FALSE)

chisq.test(df_missings_f$germbornr, df_missings_f$missings_d)

chi <- chisq.test(df_missings_f$germbornr, df_missings_f$missings_d)

chi$observed

chi$expected

corrplot(chi$residuals, is.cor = FALSE)

chisq.test(df_missings_f$migbackr, df_missings_f$missings_d)

chi <- chisq.test(df_missings_f$migbackr, df_missings_f$missings_d)

chi$observed

chi$expected

corrplot(chi$residuals, is.cor = FALSE)

chisq.test(df_missings_f$corigin_big6, df_missings_f$missings_d)

chi <- chisq.test(df_missings_f$corigin_big6, df_missings_f$missings_d)

chi$observed

chi$expected

corrplot(chi$residuals, is.cor = FALSE)

chisq.test(df_missings_f$regionc, df_missings_f$missings_d)

chi <- chisq.test(df_missings_f$regionc, df_missings_f$missings_d)

chi$observed

chi$expected

corrplot(chi$residuals, is.cor = FALSE)

chisq.test(df_missings_f$imyear10, df_missings_f$missings_d)

chi <- chisq.test(df_missings_f$imyear10, df_missings_f$missings_d)

chi$observed

chi$expected

corrplot(chi$residuals, is.cor = FALSE)

chisq.test(df_missings_f$imgrpr, df_missings_f$missings_d)

chi <- chisq.test(df_missings_f$imgrpr, df_missings_f$missings_d)

chi$observed

chi$expected

corrplot(chi$residuals, is.cor = FALSE)

chisq.test(df_missings_f$resistatr, df_missings_f$missings_d)

chi <- chisq.test(df_missings_f$resistatr, df_missings_f$missings_d)

chi$observed

chi$expected

corrplot(chi$residuals, is.cor = FALSE)

chisq.test(df_missings_f$parentr, df_missings_f$missings_d)

chi <- chisq.test(df_missings_f$parentr, df_missings_f$missings_d)

chi$observed

chi$expected

corrplot(chi$residuals, is.cor = FALSE)

chisq.test(df_missings_f$livwpart, df_missings_f$missings_d)

chi <- chisq.test(df_missings_f$livwpart, df_missings_f$missings_d)

chi$observed

chi$expected

corrplot(chi$residuals, is.cor = FALSE)

chisq.test(df_missings_f$sexor, df_missings_f$missings_d)

chi <- chisq.test(df_missings_f$sexor, df_missings_f$missings_d)

chi$observed

chi$expected

corrplot(chi$residuals, is.cor = FALSE)

########################################

#BOOTSTRAPPING

library(ggplot2)

library(reshape2)

library(lme4)

library(compiler)

library(parallel)

library(boot)

library(lattice)

library(GGally)

# to assess the predictive ability of the model

# to estimate the standard error of a parameter estimate

#fit model to the original data

# estimate the model and store results in m

m0 <- glmer(sexr ~ bip_197_01_Reversed + bip_197_04_Reversed +

bip_197_07 + bip_197_08 +

pgexppt + plh0039 +

plh0040 + plh0174 +

plh0204_h + plh0335 + pli0043_h + pli0049_h +

pli0051 + (1 | cid),

data = dfs_analysis,

family = binomial(link='logit'),

control = glmerControl(optimizer = "bobyqa"),

nAGQ = 0)

summary(cgs)

m <- glmer(remission ~ IL6 + CRP + CancerStage + LengthofStay + Experience +

(1 | DID), data = hdp, family = binomial,

control = glmerControl(optimizer = "bobyqa"),

nAGQ = 10)

# print the mod results without correlations among fixed effects

print(m, corr = FALSE)

#get confidence intervals (CIs) with the help of the standard error

se <- sqrt(diag(vcov(m)))

# table of estimates with 95% CI

(tab <- cbind(Est = fixef(m), LL = fixef(m) - 1.96 * se, UL = fixef(m) + 1.96 *

se))

#odds ratios instead of coefficients on the logit scale, we could exponentiate the estimates and CIs.

exp(tab)

#MULITLEVEL BOOTSTRAPPING

#https://www.rdocumentation.org/packages/lme4/versions/1.1-29/topics/bootMer

if (interactive()) {

fm01ML <- glmer(sexr ~ bip_197_01_Reversed + bip_197_04_Reversed +

bip_197_07 + bip_197_08 +

pgexppt + plh0039 +

plh0040 + plh0174 +

plh0204_h + plh0335 + pli0043_h + pli0049_h +

pli0051 + (1 | cid),

data = dfs_analysis,

family = binomial(link='logit'),

control = glmerControl(optimizer = "bobyqa"),

nAGQ = 0)}

## see ?"profile-methods"

mySumm <- function(.) { s <- sigma(.)

c(beta =getME(., "beta"), sigma = s, sig01 = unname(s * getME(., "theta"))) }

(t0 <- mySumm(fm01ML)) # just three parameters

## alternatively:

mySumm2 <- function(.) {

c(beta=fixef(.),sigma=sigma(.), sig01=sqrt(unlist(VarCorr(.))))

}

set.seed(101)

## 3.8s (on a 5600 MIPS 64bit fast(year 2009) desktop "AMD Phenom(tm) II X4 925"):

system.time( boo01 <- bootMer(fm01ML, mySumm, nsim = 1000) )

## to "look" at it

if (requireNamespace("boot")) {

boo01}

## note large estimated bias for sig01

## (~30% low, decreases _slightly_ for nsim = 1000)

## extract the bootstrapped values as a data frame ...

head(as.data.frame(boo01))
