# Supplementary material for: Development of a gender score in a representative German population sample and its association with diverse social positions
Source: Front Epidemiol. 2022 Aug 24;2:914819. doi: 10.3389/fepid.2022.914819 (PMC10910995; doi:10.3389/fepid.2022.914819)
Supplement: Supplementary file 4 [file Table_4.DOCX]

Supplementary Material 4: Findings of the principal component analysis

Table 1 Kaiser-Meyer-Olkin Measure of Sampling Adequacy (MSA) per item in ascending order, SOEP, 2018 (n=19426)

| **Item** | **MSAi** |
| --- | --- |
| Working experience part-time employment | 0.52 |
| Children below the age of 3 suffer if their mother works | 0.58 |
| Hours weekdays leisure. hobbies | 0.59 |
| Children below the age of 6 suffer if their mother works | 0.59 |
| Paid work in last 7 days | 0.59 |
| Hours weekdays care for persons | 0.61 |
| Working experience full-time employment | 0.62 |
| Hours weekdays housework | 0.62 |
| Hours weekdays repairs | 0.63 |
| Willingness to take risks | 0.64 |
| Worried about consequences from climate change | 0.67 |
| Worried about environment | 0.68 |
| Satisfaction with amount of leisure time | 0.74 |
| Best if man and woman work the same amount so they can share the responsibility | 0.77 |
| Satisfaction with standard of living | 0.78 |
| Worried about own retirement pension | 0.78 |
| Worried about crime in Germany | 0.78 |
| Worried about global terrorism | 0.78 |
| Satisfaction with housework | 0.79 |
| A same-sex couple can raise a child just as well as a man and woman | 0.79 |
| Satisfaction with personal income | 0.79 |
| Worried about finances | 0.80 |
| I think it is good that marriages between two women or two men are legally recognised | 0.80 |
| Nowadays can't trust anyone | 0.81 |
| Most people are exploitative vs. fair | 0.81 |
| It would be good for society if transgender people were recognized as normal | 0.83 |
| A single parent can raise a child just as well as two parents together | 0.84 |
| Worried about peace | 0.86 |
| A person who is living with their partner for the long term should get married | 0.90 |
| **Overall** | 0.74 |

*Table 2 Correlation matrix of the gender related variables included in the principal component analysis, SOEP, 2018 (n=19426)*

|  | **I1** | **I2** | **I3** | **I4** | **I5** | **I6** | **I7** | **I8** | **I9** | **I10** | **I11** | **I12** | **I13** | **I14** | **I15** | **I16** | **I17** | **I18** | **I19** | **I20** | **I21** | **I22** | **I23** | **I24** | **I25** | **I26** | **I27** | **I28** | **I29** |
| --- | --- | --- | --- | --- | --- | --- | --- | --- | --- | --- | --- | --- | --- | --- | --- | --- | --- | --- | --- | --- | --- | --- | --- | --- | --- | --- | --- | --- | --- |
| **I1** | 1 | -0.23 | -0.13 | 0.17 | 0.15 | 0.01 | -0.24 | -0.21 | 0.10 | -0.10 | 0.14 | 0.02 | 0.06 | 0.05 | -0.01 | -0.09 | -0.09 | 0.05 | 0.09 | 0.01 | 0.07 | -0.04 | -0.01 | -0.02 | 0.06 | 0.04 | 0.03 | 0.06 | 0.01 |
| **I2** |  | 1 | 0.34 | -0.20 | -0.18 | 0.13 | 0.68 | 0.65 | -0.18 | 0.07 | -0.12 | 0.05 | -0.12 | -0.13 | -0.03 | 0.12 | 0.18 | 0.09 | 0.01 | 0.07 | 0.02 | 0.15 | 0.09 | 0.04 | 0.00 | -0.04 | -0.02 | -0.10 | 0.01 |
| **I3** |  |  | 1 | -0.16 | -0.15 | 0.16 | 0.46 | 0.31 | -0.14 | 0.06 | 0.00 | -0.07 | -0.01 | -0.02 | -0.01 | -0.01 | 0.00 | -0.02 | -0.01 | -0.05 | 0.02 | -0.03 | -0.02 | 0.01 | -0.08 | 0.06 | 0.00 | -0.09 | 0.03 |
| **I4** |  |  |  | 1 | 0.78 | -0.09 | -0.22 | -0.17 | 0.06 | -0.06 | 0.10 | -0.07 | -0.01 | 0.00 | -0.02 | -0.11 | -0.13 | -0.07 | -0.01 | -0.06 | 0.02 | -0.12 | -0.10 | 0.01 | -0.03 | 0.02 | 0.01 | 0.02 | 0.02 |
| **I5** |  |  |  |  | 1 | -0.07 | -0.20 | -0.14 | 0.01 | -0.04 | 0.07 | -0.07 | -0.02 | -0.01 | -0.03 | -0.10 | -0.12 | -0.05 | 0.01 | -0.07 | 0.01 | -0.10 | -0.11 | 0.01 | -0.04 | 0.02 | 0.01 | 0.01 | 0.01 |
| **I6** |  |  |  |  |  | 1 | 0.23 | 0.19 | 0.08 | -0.01 | 0.07 | -0.02 | -0.07 | -0.07 | -0.07 | -0.05 | -0.04 | -0.01 | 0.02 | -0.02 | 0.05 | -0.01 | -0.02 | 0.01 | -0.01 | 0.03 | 0.01 | -0.01 | 0.06 |
| **I7** |  |  |  |  |  |  | 1 | 0.64 | -0.16 | 0.07 | -0.08 | 0.01 | -0.10 | -0.11 | -0.03 | 0.09 | 0.13 | 0.04 | -0.01 | 0.02 | 0.02 | 0.09 | 0.05 | 0.03 | -0.03 | -0.01 | -0.01 | -0.09 | 0.03 |
| **I8** |  |  |  |  |  |  |  | 1 | -0.09 | 0.10 | -0.05 | 0.06 | -0.14 | -0.15 | -0.08 | 0.06 | 0.13 | 0.08 | 0.00 | 0.06 | 0.04 | 0.15 | 0.09 | 0.01 | 0.02 | 0.00 | 0.00 | -0.06 | 0.04 |
| **I9** |  |  |  |  |  |  |  |  | 1 | -0.26 | 0.12 | 0.15 | -0.01 | -0.01 | -0.04 | -0.11 | -0.12 | 0.01 | 0.03 | 0.16 | 0.08 | -0.03 | 0.04 | -0.04 | 0.16 | -0.12 | 0.05 | 0.29 | 0.03 |
| **I10** |  |  |  |  |  |  |  |  |  | 1 | -0.10 | -0.02 | -0.08 | -0.08 | -0.07 | -0.02 | 0.00 | 0.00 | -0.07 | -0.07 | -0.01 | 0.05 | 0.06 | -0.10 | -0.05 | 0.29 | 0.05 | -0.01 | -0.03 |
| **I11** |  |  |  |  |  |  |  |  |  |  | 1 | 0.00 | -0.04 | -0.03 | -0.06 | -0.10 | -0.09 | -0.06 | -0.03 | -0.15 | 0.19 | -0.07 | -0.01 | -0.09 | 0.15 | 0.30 | 0.06 | 0.07 | 0.23 |
| **I12** |  |  |  |  |  |  |  |  |  |  |  | 1 | 0.08 | 0.06 | 0.12 | 0.16 | 0.22 | 0.42 | 0.15 | 0.45 | 0.22 | 0.22 | 0.25 | 0.03 | 0.58 | -0.08 | -0.01 | 0.08 | 0.04 |
| **I13** |  |  |  |  |  |  |  |  |  |  |  |  | 1 | 0.78 | 0.48 | 0.28 | 0.21 | -0.01 | 0.04 | 0.02 | 0.00 | -0.02 | -0.01 | 0.01 | 0.14 | -0.06 | -0.01 | -0.03 | -0.03 |
| **I14** |  |  |  |  |  |  |  |  |  |  |  |  |  | 1 | 0.51 | 0.29 | 0.20 | -0.03 | 0.04 | -0.01 | 0.00 | -0.03 | -0.02 | 0.01 | 0.13 | -0.05 | -0.01 | -0.02 | -0.04 |
| **I15** |  |  |  |  |  |  |  |  |  |  |  |  |  |  | 1 | 0.45 | 0.40 | 0.00 | 0.04 | 0.04 | 0.01 | 0.04 | 0.03 | 0.04 | 0.16 | -0.09 | -0.02 | -0.04 | -0.03 |
| **I16** |  |  |  |  |  |  |  |  |  |  |  |  |  |  |  | 1 | 0.60 | 0.05 | 0.02 | 0.08 | 0.00 | 0.18 | 0.14 | 0.06 | 0.19 | -0.11 | -0.04 | -0.06 | -0.02 |
| **I17** |  |  |  |  |  |  |  |  |  |  |  |  |  |  |  |  | 1 | 0.13 | 0.04 | 0.12 | 0.03 | 0.27 | 0.22 | 0.06 | 0.23 | -0.12 | -0.03 | -0.07 | -0.02 |
| **I18** |  |  |  |  |  |  |  |  |  |  |  |  |  |  |  |  |  | 1 | 0.34 | 0.56 | 0.40 | 0.21 | 0.20 | 0.04 | 0.31 | -0.05 | -0.02 | 0.06 | 0.01 |
| **I19** |  |  |  |  |  |  |  |  |  |  |  |  |  |  |  |  |  |  | 1 | 0.29 | 0.35 | 0.09 | 0.09 | 0.06 | 0.13 | -0.03 | -0.02 | 0.03 | 0.02 |
| **I20** |  |  |  |  |  |  |  |  |  |  |  |  |  |  |  |  |  |  |  | 1 | 0.26 | 0.19 | 0.20 | 0.04 | 0.35 | -0.15 | -0.01 | 0.05 | 0.00 |
| **I21** |  |  |  |  |  |  |  |  |  |  |  |  |  |  |  |  |  |  |  |  | 1 | 0.11 | 0.15 | 0.02 | 0.22 | 0.02 | -0.05 | 0.08 | 0.20 |
| **I22** |  |  |  |  |  |  |  |  |  |  |  |  |  |  |  |  |  |  |  |  |  | 1 | 0.38 | 0.04 | 0.19 | -0.05 | -0.03 | -0.02 | 0.04 |
| **I23** |  |  |  |  |  |  |  |  |  |  |  |  |  |  |  |  |  |  |  |  |  |  | 1 | 0.02 | 0.21 | -0.01 | -0.03 | 0.02 | 0.02 |
| **I24** |  |  |  |  |  |  |  |  |  |  |  |  |  |  |  |  |  |  |  |  |  |  |  | 1 | 0.02 | -0.15 | -0.02 | 0.01 | 0.00 |
| **I25** |  |  |  |  |  |  |  |  |  |  |  |  |  |  |  |  |  |  |  |  |  |  |  |  | 1 | -0.04 | 0.00 | 0.07 | 0.09 |
| **I26** |  |  |  |  |  |  |  |  |  |  |  |  |  |  |  |  |  |  |  |  |  |  |  |  |  | 1 | 0.09 | 0.07 | 0.04 |
| **I27** |  |  |  |  |  |  |  |  |  |  |  |  |  |  |  |  |  |  |  |  |  |  |  |  |  |  | 1 | 0.06 | -0.04 |
| **I28** |  |  |  |  |  |  |  |  |  |  |  |  |  |  |  |  |  |  |  |  |  |  |  |  |  |  |  | 1 | -0.02 |
| **I29** |  |  |  |  |  |  |  |  |  |  |  |  |  |  |  |  |  |  |  |  |  |  |  |  |  |  |  |  | 1 |
| Legend for items included in the correlation matrix can be found in Table 3 (p. 3) | | | | | | | | | | | | | | | | | | | | | | | | | | | | | |

*Table 3 Legend for the items included in the correlation matrix on gender-related variables, SOEP, 2018*

| I1 | A person who is living with their partner for the long term should get married |
| --- | --- |
| I2 | I think it is good that marriages between two women or two men are legally recog |
| I3 | A single parent can raise a child just as well as two parents together |
| I4 | Children below the age of 6 suffer if their mother works |
| I5 | Children below the age of 3 suffer if their mother works |
| I6 | Best if man and woman work the same amount so they can share the responsibility |
| I7 | A same-sex couple can raise a child just as well as a man and woman |
| I8 | It would be good for society if transgender people were recognized as normal |
| I9 | Working experience full-time employment |
| I10 | Working experience part-time employment |
| I11 | Paid work in last 7 days |
| I12 | Worried about finances |
| I13 | Worried about environment |
| I14 | Worried about consequences from climate change |
| I15 | Worried about peace |
| I16 | Worried about global terrorism |
| I17 | Worried about crime in Germany |
| I18 | Satisfaction with standard of living |
| I19 | Satisfaction with housework |
| I20 | Satisfaction with personal income |
| I21 | Satisfaction with amount of leisure time |
| I22 | Nowadays can't trust anyone |
| I23 | Most people are exploitative vs. fair |
| I24 | Willingness to take risks |
| I25 | Worried about own retirement pension |
| I26 | Hours weekdays housework |
| I27 | Hours weekdays care for persons |
| I28 | Hours weekdays repairs |
| I29 | Hours weekdays leisure. hobbies |

*Table 4 Findings of the Principal Component Analysis - importance of components by standard deviation, proportion of variance and cumulative proportion, SOEP, 2018 (n=19426)*

| **Component** | **Standard deviation** | **Proportion of Variance** | **Cumulative Proportion** |
| --- | --- | --- | --- |
| **Comp.1** | 1.90 | 0.12 | 0.12 |
| **Comp.2** | 1.77 | 0.11 | 0.23 |
| **Comp.3** | 1.60 | 0.09 | 0.32 |
| **Comp.4** | 1.29 | 0.06 | 0.38 |
| **Comp.5** | 1.24 | 0.05 | 0.43 |
| **Comp.6** | 1.21 | 0.05 | 0.48 |
| **Comp.7** | 1.15 | 0.05 | 0.53 |
| **Comp.8** | 1.07 | 0.04 | 0.57 |
| **Comp.9** | 1.01 | 0.04 | 0.60 |
| **Comp.10** | 0.99 | 0.03 | 0.63 |
| **Comp.11** | 0.98 | 0.03 | 0.67 |
| **Comp.12** | 0.95 | 0.03 | 0.70 |
| **Comp.13** | 0.94 | 0.03 | 0.73 |
| **Comp.14** | 0.92 | 0.03 | 0.76 |
| **Comp.15** | 0.88 | 0.03 | 0.79 |
| **Comp.16** | 0.84 | 0.02 | 0.81 |
| **Comp.17** | 0.80 | 0.02 | 0.83 |
| **Comp.18** | 0.78 | 0.02 | 0.85 |
| **Comp.19** | 0.77 | 0.02 | 0.87 |
| **Comp.20** | 0.74 | 0.02 | 0.89 |
| **Comp.21** | 0.69 | 0.02 | 0.91 |
| **Comp.22** | 0.67 | 0.02 | 0.92 |
| **Comp.23** | 0.63 | 0.01 | 0.94 |
| **Comp.24** | 0.62 | 0.01 | 0.95 |
| **Comp.25** | 0.61 | 0.01 | 0.96 |
| **Comp.26** | 0.59 | 0.01 | 0.98 |
| **Comp.27** | 0.54 | 0.01 | 0.98 |
| **Comp.28** | 0.47 | 0.01 | 0.99 |
| **Comp.29** | 0.46 | 0.01 | 1.00 |

*Figure 1 Screeplot of the Principal Component Analysis to construct the gender score, SOEP, 2018*

*Table 5 Factor loadings of the components identified in the Principal Component Analysis, SOEP, 2018*

|  | **Comp.1** | **Comp.2** | **Comp.3** | **Comp.4** | **Comp.5** | **Comp.6** | **Comp.7** | **Comp.8** | **Comp.9** |
| --- | --- | --- | --- | --- | --- | --- | --- | --- | --- |
| **Symbolic relations (attitudes and norms)** |  |  |  |  |  |  |  |  |  |
| A person who is living with their partner for the long term should get married |  |  |  |  |  |  |  |  | 0.27 |
| I think it is good that marriages between two women or two men are legally recognised | -0.25 | -0.36 |  |  |  |  |  |  |  |
| A single parent can raise a child just as well as two parents together |  | -0.27 |  |  |  |  |  |  |  |
| Children below the age of 6 suffer if their mother works |  |  |  |  | 0.61 |  |  |  |  |
| Children below the age of 3 suffer if their mother works |  |  |  |  | 0.63 |  |  |  |  |
| Best if man and woman work the same amount so they can share the responsibility |  |  |  |  |  |  |  |  |  |
| A same-sex couple can raise a child just as well as a man and woman |  | -0.38 |  |  |  |  |  |  |  |
| It would be good for society if transgender people were recognized as normal |  | -0.35 |  |  |  |  |  |  |  |
| **Economic and power relations (access to resources and participation)** |  |  |  |  |  |  |  |  |  |
| Working experience full-time employment |  |  |  |  |  | 0.42 | -0.27 |  |  |
| Working experience part-time employment |  |  |  |  |  | -0.53 |  |  |  |
| Paid work in last 7 days |  |  |  | 0.57 |  |  |  |  |  |
| Hours weekdays housework |  |  |  | 0.36 |  | -0.42 |  |  |  |
| Hours weekdays care for persons |  |  |  |  |  |  |  | -0.46 | 0.36 |
| Hours weekdays repairs |  |  |  |  |  |  |  | -0.32 | 0.26 |
| Hours weekdays leisure. hobbies |  |  |  | 0.38 |  |  |  | 0.47 | -0.27 |
| **Affective relations (emotional resources)** |  |  |  |  |  |  |  |  |  |
| Worried about finances | -0.30 |  |  |  |  |  |  |  | -0.25 |
| Worried about environment |  | 0.28 | -0.35 |  |  |  |  |  |  |
| Worried about consequences from climate change |  | 0.28 | -0.37 |  |  |  |  |  |  |
| Worried about peace |  |  | -0.35 |  |  |  |  |  |  |
| Worried about global terrorism | -0.26 |  | -0.30 |  |  |  |  |  |  |
| Worried about crime in Germany | -0.31 |  |  |  |  |  | -0.27 |  |  |
| Worried about own retirement pension | -0.26 |  |  |  |  |  |  |  | -0.25 |
| Satisfaction with standard of living | -0.28 |  | 0.29 |  |  |  | 0.27 |  |  |
| Satisfaction with housework |  |  |  |  |  |  | 0.41 |  | 0.33 |
| Satisfaction with personal income | -0.28 |  | 0.26 |  |  |  |  |  |  |
| Satisfaction with amount of leisure time |  |  | 0.27 |  |  |  |  | 0.32 |  |
| Nowadays can't trust anyone |  |  |  |  |  |  | -0.29 |  |  |
| Most people are exploitative vs. fair |  |  |  |  |  |  | -0.26 |  |  |
| Willingness to take risks |  |  |  |  |  |  |  |  | 0.41 |
| Only factor loadings >0.25 in components with eigenvalues above 1 displayed | | | | | | | | | |
