# Supplementary material for: Development of a gender score in a representative German population sample and its association with diverse social positions
Source: Front Epidemiol. 2022 Aug 24;2:914819. doi: 10.3389/fepid.2022.914819 (PMC10910995; doi:10.3389/fepid.2022.914819)
Supplement: Supplementary file 5 [file Table_5.DOCX]

Supplementary Material 5: Gendered social practices by social positions

# Sociodemographics


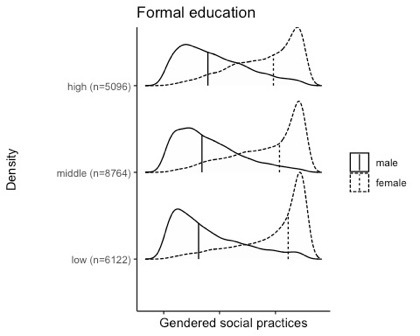


Figure 2 Gendered social practices by formal educational attainment, SOEP, 2018 (n=19,982)


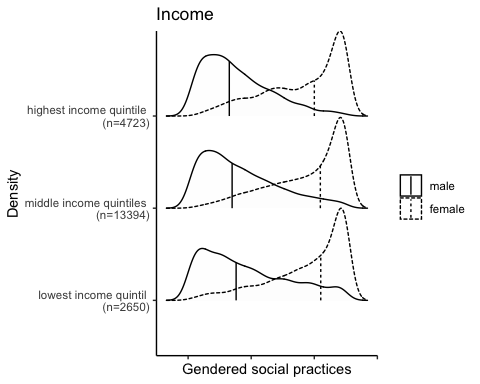


Figure 3 Gendered social practices by pre-government household income, SOEP, 2018 (n=20,767)


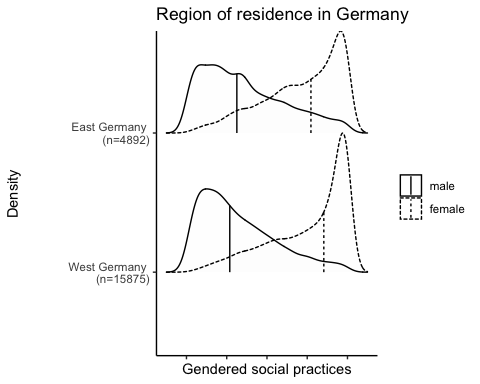


Figure 4 Gendered social practices by region of residence in Germany, SOEP, 2018 (n=20,767)

# Migration status


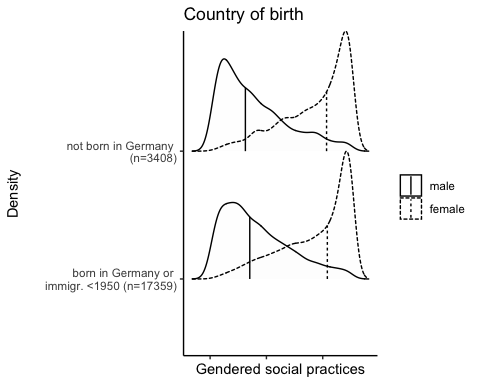


Figure 5 Gendered social practices by country of birth (dichotomised Germany vs. Non-Germany), SOEP, 2018 (n=20,767)


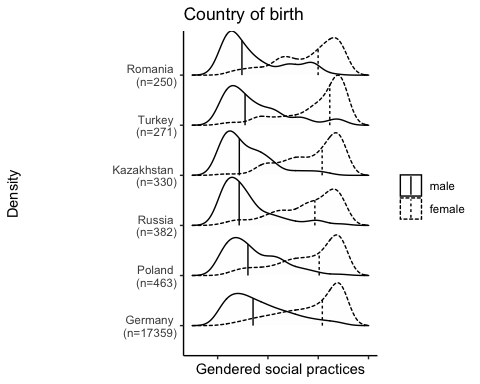


Figure 6 Gendered social practices by country of birth (6 most prevalent countries of origin in Germany), SOEP, 2018 (n=19,055)


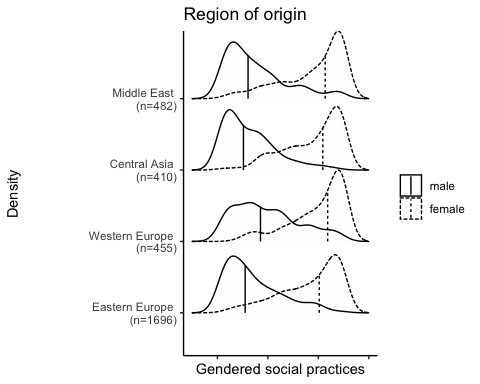


Figure 7 Gendered social practices by region of origin, SOEP, 2018 (n=20,402)


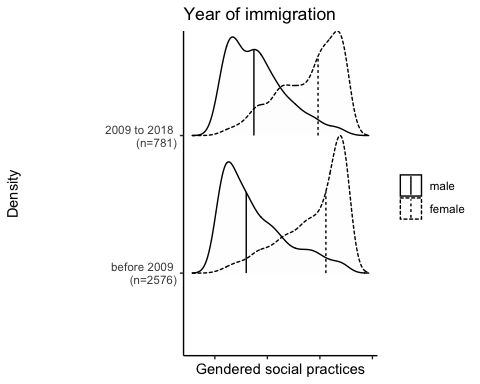


Figure 8 Gendered social practices by year of immigration, SOEP, 2018 (n=3,357)


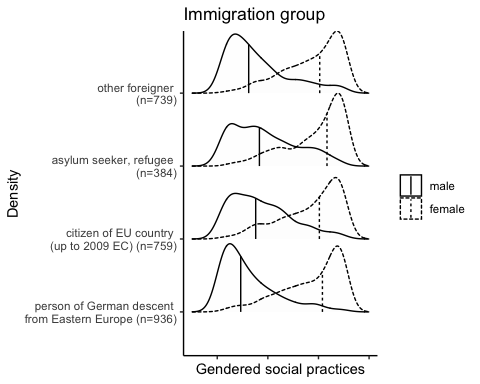


Figure 9 Gendered social practices by immigration group, SOEP, 2018 (n=2,898)


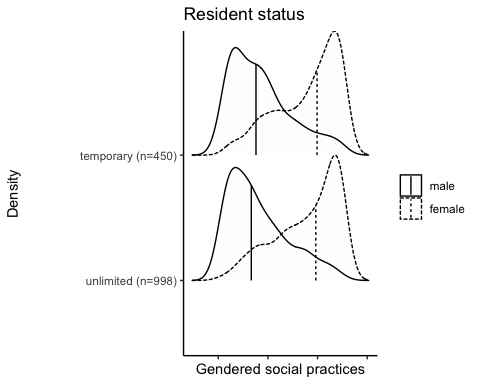


Figure 10 Gendered social practices by residence status, SOEP, 2018 (n=1,448)

# Partnership and parenthood


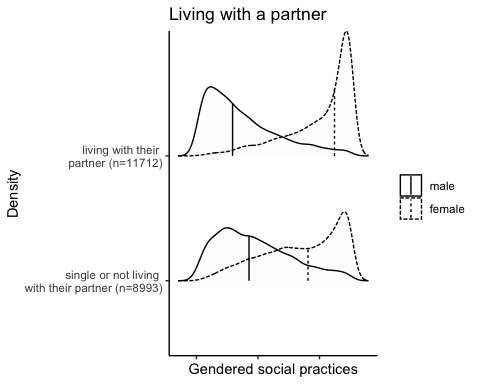


Figure 11 Gendered social practices by cohabitation with a partner, SOEP, 2018 (n=20,705)


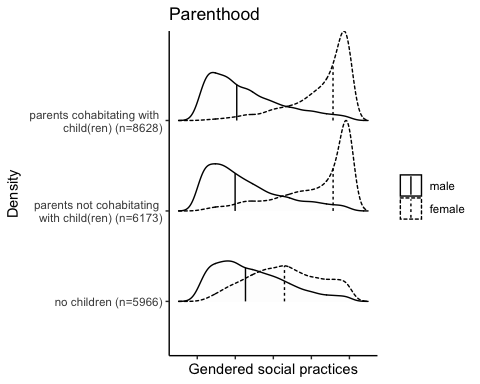


Figure 13 Gendered social practices by parenthood, SOEP, 2018 (n=20,767)
