# Supplementary material for: Development of a gender score in a representative German population sample and its association with diverse social positions
Source: Front Epidemiol. 2022 Aug 24;2:914819. doi: 10.3389/fepid.2022.914819 (PMC10910995; doi:10.3389/fepid.2022.914819)
Supplement: Supplementary file 6 [file Table_6.DOCX]

Supplementary Material 6: Gendered social practices by social positions - cross-classifications

1. **Migration status stratified by age, parenthood and cohabitation status with a partner**


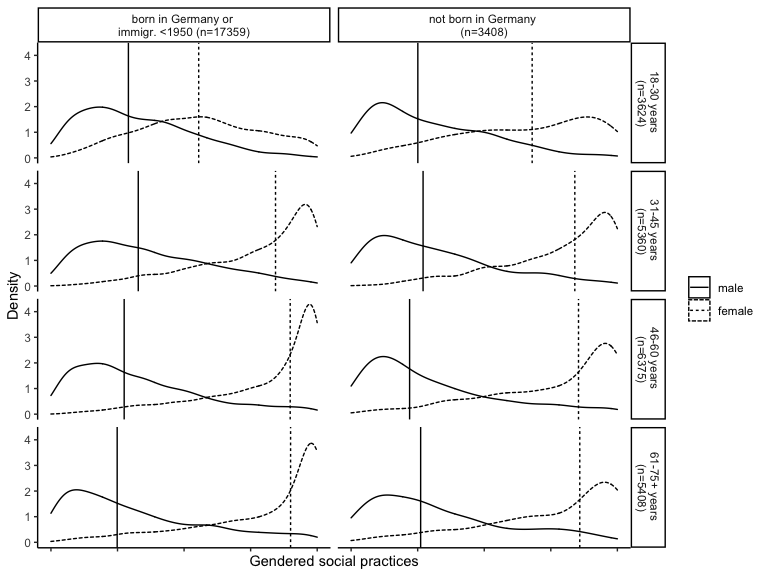


*Figure 1 Gendered social practices by country of birth and age, SOEP, 2018 (n=20,767)*


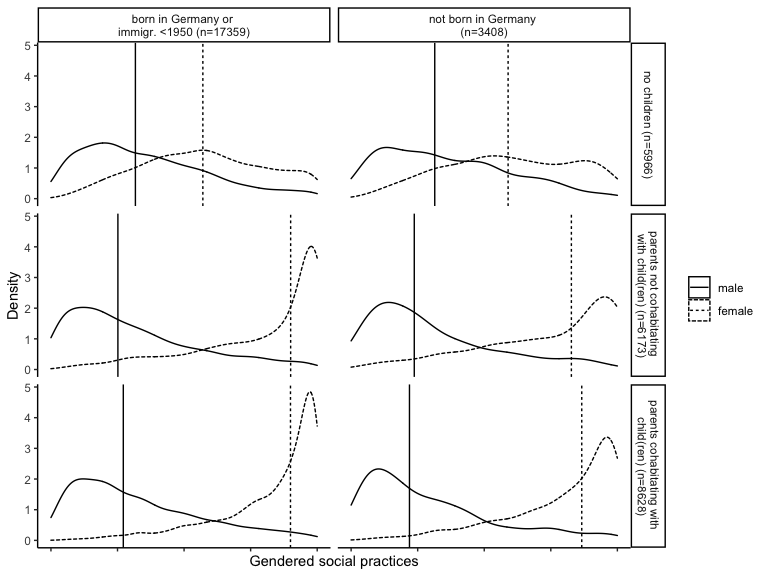


*Figure 2 Gendered social practices by country of birth and parenthood, SOEP, 2018 (n=20,767)*


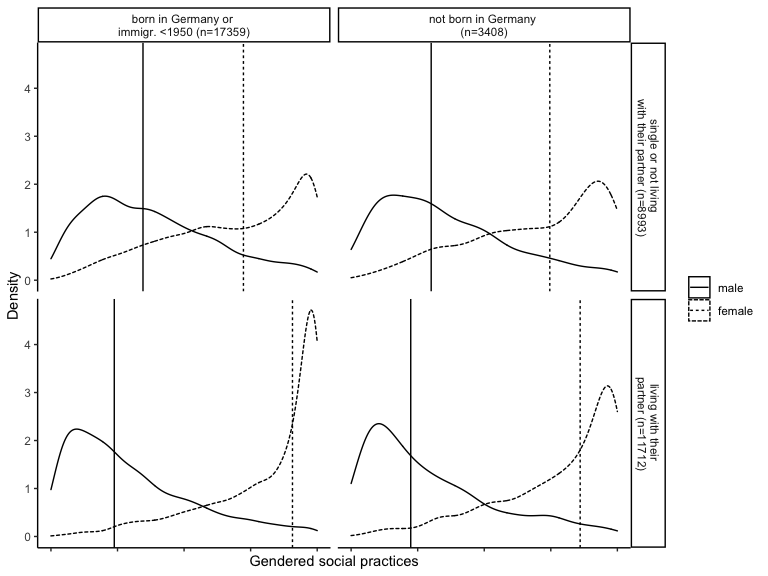


*Figure 3 Gendered social practices by country of birth and cohabitation status with a partner, SOEP, 2018 (n=20,705)*

1. **Age stratified by income, region of residence in Germany, parenthood and cohabitation status with a partner**

*
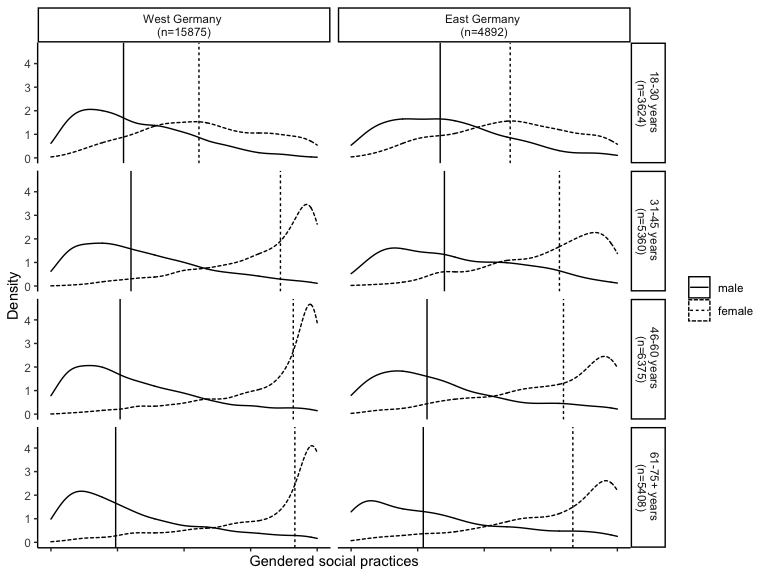
*

*Figure 4 Gendered social practices by age and region of residence in Germany, SOEP, 2018 (n=20,767)*


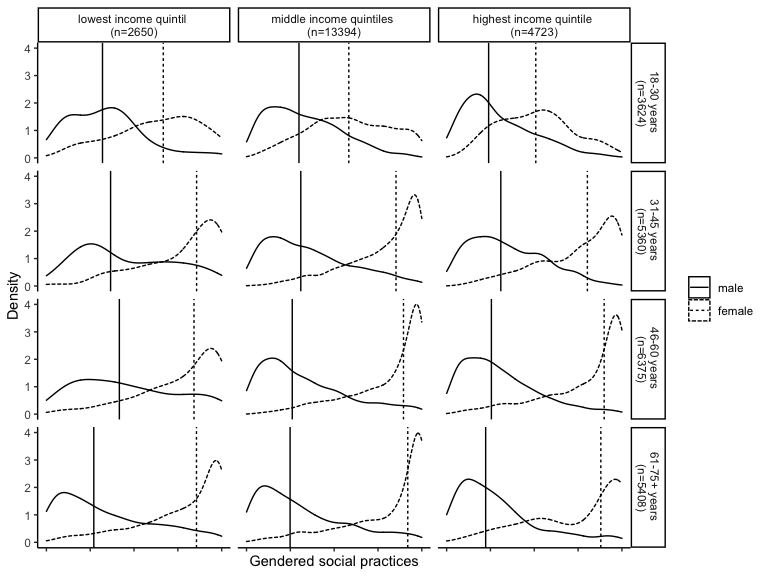


*Figure 5 Gendered social practices by age and pre-government household income, SOEP, 2018 (n=20,767)*


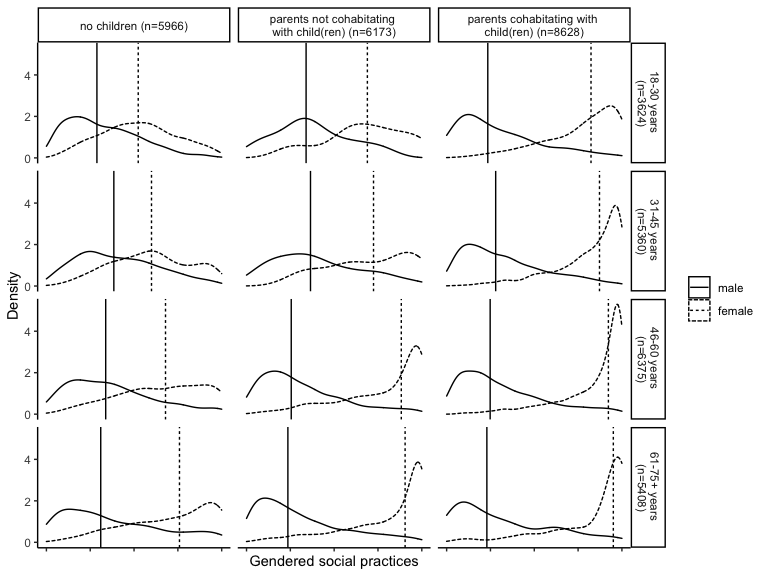


*Figure 6 Gendered social practices by age and parenthood, SOEP, 2018 (n=20,767)*


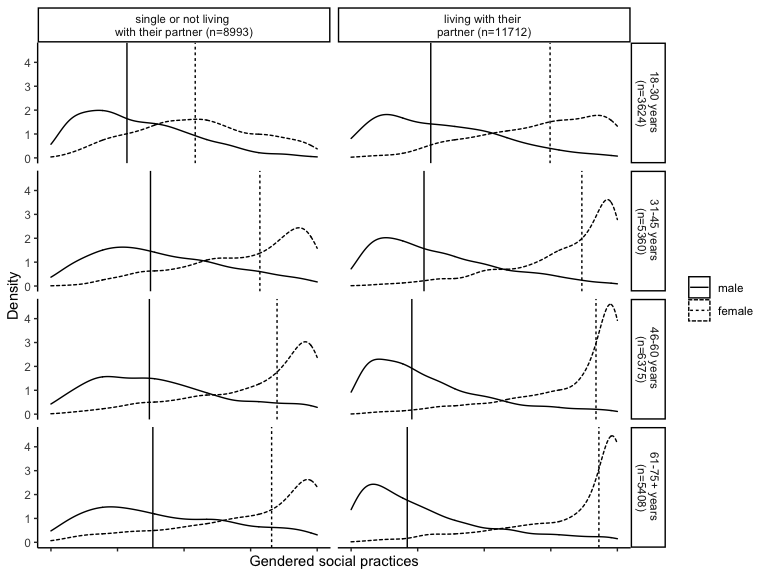


*Figure 8 Gendered social practices by age and cohabitation status with a partner, SOEP, 2018 (n=20,705)*
