# Supplementary material for: Development of a gender score in a representative German population sample and its association with diverse social positions
Source: Front Epidemiol. 2022 Aug 24;2:914819. doi: 10.3389/fepid.2022.914819 (PMC10910995; doi:10.3389/fepid.2022.914819)
Supplement: Supplementary file 7 [file Table_7.DOCX]

Supplementary Material 7: Sensitivity analysis - comparison of different gender scores

*Table 1 Comparison of different regression models to estimate the composite gender score, SOEP, 2018 (n=19,426)*

|  | M0 |  | M1 |  | M2 |  | **M3** |  | M4 |  |
| --- | --- | --- | --- | --- | --- | --- | --- | --- | --- | --- |
| **Symbolic relations (attitudes and norms)** |  |  |  |  |  |  |  |  |  |  |
| A person who is living with their partner for the long term should get married | -0.04 | *** | -0.04 | *** | -0.04 | *** | **-0.04** | ******* | -0.04 | *** |
| I think it is good that marriages between two women or two men are legally recognized | -0.03 | *** | -0.04 | ** |  |  |  |  |  |  |
| A single parent can raise a child just as well as two parents together |  |  | 0.18 | *** | 0.19 | *** |  |  |  |  |
| Children below the age of 6 suffer if their mother works | -0.07 | *** | -0.11 | *** | -0.11 | *** | **-0.13** | ******* | -0.13 | *** |
| Children below the age of 3 suffer if their mother works | -0.05 | *** |  |  |  |  |  |  |  |  |
| Best if man and woman work the same amount so they can share the responsibility | 0.03 | ** |  |  |  |  |  |  |  |  |
| A same-sex couple can raise a child just as well as a man and woman |  |  | 0.05 | *** | 0.06 | *** | **0.13** | ******* | 0.13 | *** |
| It would be good for society if transgender people were recognized as normal | 0.12 | ** | 0.12 | *** | 0.09 | *** | **0.10** | ******* | 0.10 | *** |
| **Economic and power relations (access to resources and participation)** |  |  |  |  |  |  |  |  |  |  |
| Working experience full-time employment | 0.16 | *** | 0.16 | *** |  |  |  |  |  |  |
| Working experience part-time employment | -0.04 | *** | -0.04 | *** | 0.18 | *** | **0.18** | ******* | 0.18 | *** |
| Paid work in last 7 days | 0.28 | *** | 0.28 | *** |  |  |  |  | 0.12 | ** |
| Hours weekdays housework | 1.18 | *** | 1.18 | *** | 1.20 | *** | **1.20** | ******* | 1.19 | *** |
| Hours weekdays care for persons |  |  |  |  |  |  |  |  |  |  |
| Hours weekdays repairs | -0.64 | *** | -0.64 | *** | -0.79 | *** | **-0.80** | ******* | -0.79 | *** |
| Hours weekdays leisure, hobbies | -0.10 | *** | -0.10 | *** | -0.09 | *** | **-0.08** | ******* | -0.09 | *** |
| **Affective relations (emotional resources)** |  |  |  |  |  |  |  |  |  |  |
| Worried about finances |  |  |  |  | -0.13 | *** |  |  |  |  |
| Worried about environment |  |  |  |  | 0.09 | ** |  |  |  |  |
| Worried about consequences from climate change |  |  |  |  |  |  |  |  |  |  |
| Worried about peace | -0.19 | *** | -0.15 | *** | -0.20 | *** |  |  |  |  |
| Worried about global terrorism | -0.32 | *** | -0.31 | *** | -0.29 | *** | **-0.30** | ******* |  |  |
| Worried about crime in Germany | -0.15 | *** | -0.16 | *** |  |  | **-0.10** | ****** | -0.26 | *** |
| Worried about own retirement pension |  |  |  |  |  |  | **-0.08** | ****** | -0.11 | *** |
| Satisfaction with standard of living | 0.13 | *** | 0.12 | *** | 0.16 | *** |  |  |  |  |
| Satisfaction with housework | -0.10 | *** | -0.10 | *** | -0.09 | *** | **-0.06** | ******* | -0.06 | *** |
| Satisfaction with personal income |  |  |  |  | -0.05 | *** |  |  |  |  |
| Satisfaction with amount of leisure time |  |  |  |  |  |  |  |  |  |  |
| Nowadays can't trust anyone |  |  |  |  |  |  |  |  |  |  |
| Most people are exploitative vs. fair |  |  |  |  |  |  |  |  |  |  |
| Willingness to take risks | -0.16 | *** | -0.16 | *** | -0.13 | *** | **-0.13** | ******* | -0.13 | *** |
|  |  |  |  |  |  |  |  |  |  |  |
| Model fit AIC | 15929.2 | | 15945.4 | | 16613.2 | | **17042.2** | | 17110.9 | |
| BIC | 16110.3 | | 16102.9 | | 16762.8 | | **17160.3** | | 17229.0 | |
| M0: statistical selection in backwards regression (p<0.01), M1: a priori exclusion of moderately correlated items ('Children below the age of 3 suffer if their mother works', 'Worried about consequences from climate change'), M2 (final model): as in M1 plus content-based exclusion of 'Working experience full-time employment' (higher AIC than exclusion of 'Working experience part-time employment'), M3: as in M2 plus content-based exclusion of 'A single parent can raise a child just as well as two parents together', 'Worried about peace' (both indefinite meaning) and 'Nowadays can't trust anyone', 'Most people are exploitative vs. fair' (excluded in all previous models), M4: as in M3 plus exclusion of variables not relevant in previous models ('Worried about environment', 'Worried about global terrorism') | | | | | | | | | | |
| significant at the level of <0.001 (***), 0.01 (**), 0.05 (*) |  |  |  |  |  |  |  |  |  |  |

*Figure 1 Distributions of the composite gender scores, SOEP, 2018 (n=19,426)*

*Figure 2 Comparison of different distributions of composite gender scores by sex assigned at birth, SOEP, 2018 (n=19,426)*
