# Supplementary material for: Development of a gender score in a representative German population sample and its association with diverse social positions
Source: Front Epidemiol. 2022 Aug 24;2:914819. doi: 10.3389/fepid.2022.914819 (PMC10910995; doi:10.3389/fepid.2022.914819)
Supplement: Supplementary file 8 [file Table_8.DOCX]

Supplementary Material 8: Bootstrapping

Table 1 Bootstrap statistics for logistic regression coefficients of gendered social practices associated with sex assigned at birth used to construct the gender score, German SOEP, 2018 (n=20,767)

|  | Original coefficient | Bias | Standard error |
| --- | --- | --- | --- |
| **Intercept** | -1.39 | - 4.51E+03 | 0.1230 |
| **Symbolic relations (attitudes and norms)** |  |  |  |
| A person who is living with their partner for the long term should get married | 0.04 | -1.88E+02 | 0.0090 |
| Children below the age of 6 suffer if their mother works | 0.12 | 1.61E+02 | 0.0095 |
| A same-sex couple can raise a child just as well as a man and woman | 0.13 | 3.63E+02 | 0.0118 |
| It would be good for society if transgender people were recognised as normal | 0.10 | 2.88E+02 | 0.0122 |
| **Economic and power relations (access to resources and participation)** |  |  |  |
| Working experience part-time employment | 0.18 | 1.49E+02 | 0.0049 |
| Hours/weekday housework | 1.21 | 1.09E+03 | 0.0333 |
| Hours/weekday repairs | -0.80 | -7.43E+02 | 0.0317 |
| Hours/weekday leisure, hobbies | -0.08 | -1.28E+02 | 0.0099 |
| **Affective relations (emotional resources)** |  |  |  |
| Worried about global terrorism | -0.31 | 2.97E+01 | 0.0077 |
| Worried about crime in Germany | -0.10 | -5.36E+02 | 0.0270 |
| Satisfaction with housework | -0.06 | 2.01E+03 | 0.0247 |
| Willingness to take risks | -0.13 | -9.31E+02 | 0.0256 |
| Worried about own retirement pension | -0.08 | 8.12E+01 | 0.0099 |
| Categorisation in symbolic, economic and power as well as affective relations is based on (22) | | | |

*Source: Authors’ own elaboration. Data source: Socioeconomic panel, wave v35.*
